# Radionuclides in Milk: A Global Systematic Review and Meta‐Analysis and Probabilistic Human Risk Assessment

**DOI:** 10.1002/fsn3.71770

**Published:** 2026-05-20

**Authors:** Yadolah Fakhri, Zeinab Gholami, Olga A. Malanova, Farshid Soleimani, Seid Kamal Ghadiri, Mohammadreza Gharepour, Moayed Adiban, Mansour Sarafraz

**Affiliations:** ^1^ Food Health Research Center Hormozgan University of Medical Sciences Bandar Abbas Iran; ^2^ Health and Environment Research Center Ilam University of Medical Sciences Ilam Iran; ^3^ Sechenov First Moscow State Medical University (Sechenov University) Moscow Russia; ^4^ Tobacco and Health Research Center Hormozgan University of Medical Sciences Bandar Abbas Iran; ^5^ Environmental and Occupational Health Research Center Shahroud University of Medical Sciences Shahroud Iran; ^6^ Student Research Committee, School of Public Health Shahroud University of Medical Sciences Shahroud Iran; ^7^ Department of Environmental Health Engineering, School of Public Health Ilam University of Medical Science Ilam Iran

**Keywords:** cancer risk, food safety, meta‐analysis, milk contamination, radioactive isotopes, radionuclides

## Abstract

This study conducted a systematic review and meta‐analysis of radionuclide concentrations of Cesium‐137 (Cs‐137), Cesium‐134 (Cs‐134), Iodine‐131 (I‐131), Lead‐210 (Pb‐210), and Potassium‐40 (K‐40) in milk worldwide, along with an assessment of human health risks, in accordance with the PRISMA guidelines. Pooled radionuclide concentrations were estimated using a random‐effects model, while excess lifetime cancer risk (ELCR) was calculated to evaluate carcinogenic effects using the Monte Carlo Simulation (MCS) model. A comprehensive search of Scopus, PubMed, Embase, and Web of Science identified 66 papers (200 data reports, 2000–2025) that cover milk samples from 32 countries. The global pooled mean concentrations (Bq/L) followed the order: K‐40: 57.494 > Cs‐137: 0.904 > I‐131: 0.301 > Pb‐210: 0.136 > Cs‐134: 0.032. Extreme concentrations were observed in the United Kingdom (Cs‐137: 288.000), Syria (I‐131: 76.000; K‐40: 243.500), and India (Pb‐210: 1.080), whereas Mali (Cs‐137: 0.003) and Algeria (K‐40: 2.349) showed the lowest levels. Probabilistic human health risk assessments revealed that the mean cancer risk (CR) for adults from Cs‐137 ingestion exceeded the acceptable limit (1E‐4) in the United Kingdom, Austria, South Korea, Serbia, the Czech Republic, Germany, Kazakhstan, and Iceland. For Cs‐134, the CR in Finland and France remained below the limit. The CR from K‐40 exceeded the limit in Spain, Syria, Bangladesh, Mali, Iraq, Singapore, Thailand, Japan, Italy, and Turkey. For I‐131, the limit was exceeded in Syria, Romania, Greece, and Spain. The CR of Pb‐210 exceeded the limit in Tunisia, Slovenia, India, the Czech Republic, Italy, and Syria. These disparities reflect variations in environmental contamination, nuclear legacy, and regulatory standards. The results highlight the necessity for strict monitoring, especially in high‐risk areas, and promote enhanced agricultural practices and food safety policies to reduce radionuclide exposure through milk consumption.

## Graphical abstract

Meta‐analysis and human risk assessment of radionuclides in milk.

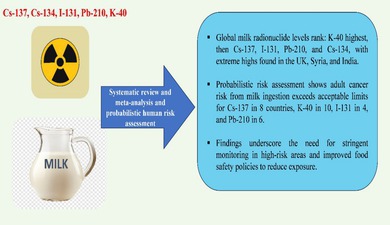

## 1 Introduction

Today, radiation from naturally occurring radioactive materials on Earth, as well as cosmic radiation, is a part of human life (Thabayneh and Hreaz 2025). Radioactive materials such as Uranium‐238 (U‐238), Thorium‐232 (Th‐232), their decay products, and Potassium‐40 (K‐40) are the main sources of natural radiation in human life (Khandaker et al. 2017). Cesium‐137 (Cs‐137), a material, enters the environment through nuclear tests and accidents. The primary route of human exposure to this hazardous radiation is through the consumption of food and water (El Afifi et al. 2023). Once inside the body, these materials penetrate tissues and can cause serious and irreversible damage to cells, increase the risk of cancer, and lead to cell death (Pervin et al. 2024; Ajayi 2009; Hilal et al. 2022). The most important long‐term effects of radiation for people exposed to more than 100 rem are carcinogenesis and tumor induction. Also, people who have been exposed to more than 10 rem of radiation have an increased risk of death (from cancer) of 0.8% over their lifetime (Desimoni et al. 2009; Ali et al. 2020). Other effects of radiation on humans include genetic damage in subsequent generations, cataracts, and reduced reproductive capacity (Sahar et al. 2016). Milk and milk‐based products contain carbohydrates, proteins, vitamins, fats, and mineral elements; thus, they are an important part of the human diet. In addition, milk is an ingredient in the composition and preparation of various foods, such as ice cream and cakes, and is the primary source of nutrition for infants and children (Pashkova et al. 2018; Thorning et al. 2016). Because milk and its products are consumed by most people worldwide due to their beneficial properties (Durusoy and Yildirim 2017; Gallagher et al. 1997; Silva, Shen, et al. 2010), these foods can be an important pathway for radionuclides to enter the human body from the environment (Biçer and Cetinkaya 2023; Duong et al. 2021). Milk and other food products can be contaminated with various substances, such as heavy metals (Kahn et al. 1965; Howard 2021), mycotoxins (Vreman et al. 1989; Barescut et al. 2005), and antibiotics (Rinky et al. 2025), through multiple pathways. On the other hand, given that large amounts of natural and artificial radioactive materials in the environment readily enter milk and its products, determining the concentrations of radioactive materials in these products is crucial for protecting against radiation and ensuring consumer safety (Gradaščević et al. 2023). The European Union has established maximum limits for radionuclides in milk and dairy products. According to this law, the maximum permitted level of radioactive Cs‐137 in milk and its products is 370 becquerels per kilogram, and for other products, it is 600 becquerels (Caridi et al. 2024). Considering the above, to manage the production, processing, and distribution of these food products (milk and dairy products) under conditions of radioactive contamination, serious measures must be implemented to reduce contamination in all sectors (Joint 2020). Numerous papers have been published worldwide on radionuclides (Cs‐137, Cs‐134, I‐131, Pb‐210, K‐40) in milk (Appendix S1). However, no comprehensive systematic review study with meta‐analysis and risk assessment has been published. Therefore, the primary objectives of this study were to conduct a meta‐analysis of radionuclide concentrations (Cs‐137, Cs‐134, I‐131, Pb‐210, K‐40) in milk across countries and to assess the associated human health risks.

## 2 Materials and Methods

### 2.1 Search Strategy

This systematic review was conducted following the PRISMA (Preferred reporting items for systematic reviews and meta‐analyses) guideline (Moher et al. 2015). We performed comprehensive searches in Web of Science, Scopus, PubMed, and Embase for studies published between January 2000 and February 2025. In addition, a search was conducted in Google Scholar to identify gray literature. Our search strategy combined terms related to radionuclides (“Radionuclides” **OR** “Radioisotopes” **OR** “Cesium‐137” **OR** “Cesium‐134” **OR** “Iodine‐131” **OR** “Lead‐210” **OR** “Potassium‐40”) **AND** (“Milk” **OR** “Dairy” **OR** “Cow milk” **OR** “Goat milk” **OR** “Breast milk”). In the first step, titles and abstracts were systematically reviewed, and pertinent full‐text articles were identified. Two independent authors assessed the selected papers, and any disagreements were resolved through the corresponding author. To ensure comprehensive inclusion, the reference sections of the incorporated papers were reviewed to identify any additional relevant papers.

### 2.2 Study Selection and Data Extraction

Eligible authors were required to meet the following criteria: descriptive study‐based studies reporting concentration of radionuclides in milk, availability of quantitative data (mean, standard deviation, and/or concentration range), and full‐text access in English. Exclusion criteria encompassed review papers, conference abstracts, book chapters, letters to the editor, and theses. Extracted data included country of origin, sample number, type of milk, type of radionuclides, mean concentration, standard deviation, minimum and maximum concentration of radionuclides, and analytical methodology. The radionuclide concentrations in milk, initially reported as mBg/L or Bq/kg, were converted to Bq/L for consistency.

### 2.3 Meta‐Analysis of Data

The meta‐analysis aimed to calculate combined radionuclide concentrations in milk using mean values and standard errors. The analysis was conducted by categorizing the data into country‐based subgroups. Heterogeneity was evaluated with the *I* ^2^ index, where values above 50% signified significant heterogeneity (Zhou et al. 2021), prompting the use of a random effects model (REM) for pooled estimates. All statistical analyses were conducted using Stata 17.0 (StataCorp, College Station, TX, USA).

### 2.4 Human Risk Assessment

Excess lifetime cancer risk (ELCR) from radionuclides in milk was calculated, as (Rahimi et al. 2023; Shoaei et al. 2023):

(1) ELCR=C×CR×RF×ED

where ELCR is the excess lifetime cancer risk; C, the concentration of radionuclides in milk (Bq/L); consumption rate of milk (kg/y) (Appendix S2); RF, Radionuclide‐specific cancer risk factors per Bq ingested (risk/Bq) (Appendix S3); and ED, exposure duration (years). An ELCR exceeding 1E‐4 indicates a substantial risk to human health.

### 2.5 Monte Carlo Simulation (MCS) Model

A health risk assessment was conducted using MCS model to account for variability and uncertainty in exposure parameters. The simulation was performed with 5000 iterations, with input parameters such as radionuclide concentrations and milk consumption rates modeled as probability distributions. Radionuclide concentrations were represented using a log‐normal distribution, while milk consumption rates were modeled using a normal distribution. In the MCS model, the mean CR was used as the criterion for presenting results (Appendix S4–S8).

## 3 Results and Discussion

### 3.1 Concentration of Radionuclides in Milk

**FIGURE 1:**
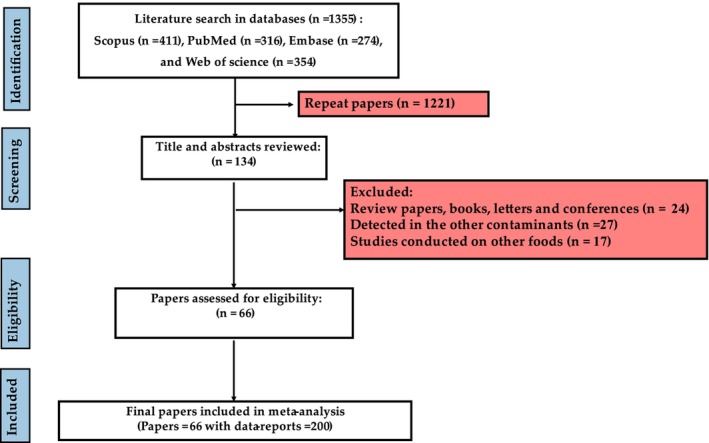
Process of selection of papers based on PRISMA.

Initially, 1355 records were identified from four databases: Scopus (*n* = 411), PubMed (*n* = 316), Embase (*n* = 274), and Web of Science (*n* = 354). After removing 1221 duplicate papers, 134 titles and abstracts were reviewed. During this stage, 68 records were excluded for various reasons, including being review papers, books, letters, or conference materials (*n* = 24), focusing on other contaminants (*n* = 27), or examining unrelated food types (*n* = 17). Ultimately, 66 papers with 200 data reports were included in the study (Figure 1). The order of elements in milk based on their average concentrations was as follows: K‐40 (57.49 Bq/L) > Cs‐137 (0.90 Bq/L) > I‐131 (0.30 Bq/L) > Pb‐210 (0.14 Bq/L) > Cs‐134 (0.03 Bq/L) (Tables 1, 2, 3, 4, 5).

**TABLE 1:** Meta‐analysis concentration of Cs‐137 in milk (Bq/L).

Country	NS^a^	ES^b^	Lower	Upper	Weight (%)	Heterogeneity statistic	Degrees of freedom	*p*	*I* ^2^ (%)
Austrian	9	52.466	34.696	70.236	0.29	2.10 × 10³	8	**< 0.001** ^**c**^	99.60
Kazakhstan	5	3.348	0.000	7.310	6.8	3.50 × 10^5^	4	**< 0.001**	100.00
Singapore	1	0.010	0.006	0.015	1.59	0.00	0	.	.
Vietnam	1	0.130	0.093	0.167	1.58	0.00	0	.	.
Thailand	1	0.637	0.574	0.700	1.56	0.00	0	.	.
India	1	0.240	0.010	0.470	1.24	0.00	0	.	.
Egypt	1	0.055	0.048	0.062	1.59	0.00	0	.	.
Mali	1	0.003	0.003	0.004	1.59	0.00	0	.	.
Tunisia	2	0.022	0.008	0.037	3.19	3.13 × 10¹	1	**< 0.001**	96.80
Russia	4	0.831	0.523	1.139	5.64	2.40 × 10^4^	3	**< 0.001**	100.00
UK	1	288.000	280.225	295.775	0	0.00	0	.	.
Bosnia and Herzegovina	1	0.070	0.064	0.076	1.59	0.00	0	.	.
Czech	1	6.010	5.930	6.090	1.54	0.00	0	.	.
Italy	4	0.349	0.124	0.574	6.26	2.40 × 10²	3	**< 0.001**	98.80
South Korea	1	23.000	22.434	23.566	0.58	0.00	0	.	.
Japan	5	0.211	0.127	0.296	7.9	1.26 × 10³	4	**< 0.001**	99.70
Romania	11	0.054	0.000	0.142	17.52	1.72 × 10^4^	10	**< 0.001**	99.90
Spain	5	0.019	0.012	0.026	7.93	2.67 × 10¹	4	**< 0.001**	85.00
Switzerland	1	0.405	0.216	0.594	1.34	0.00	0	.	.
Iceland	1	1.270	1.267	1.273	1.59	0.00	0	.	.
Malaysia	2	0.036	0.031	0.041	3.18	6.00 × 10−^2^	1	0.812	0.00
Saudi Arabia	2	0.084	0.015	0.153	3.18	7.15 × 10¹	1	**< 0.001**	98.60
Serbia	4	6.798	0.000	13.629	3.24	4.49 × 10³	3	**< 0.001**	99.90
New Zealand	1	0.075	0.071	0.079	1.59	0.00	0	.	.
Jordan	1	0.090	0.062	0.118	1.59	0.00	0	.	.
Lebanon	1	0.137	0.136	0.137	1.59	0.00	0	.	.
Greece	2	0.345	0.000	0.815	2.81	1.56 × 10¹	1	**< 0.001**	93.60
Bangladesh	1	0.050	0.037	0.063	1.59	0.00	0	.	.
USA	4	0.459	0.345	0.573	5.47	5.74 × 10˚	3	0.125	47.80
Algeria	1	0.023	0.019	0.028	1.59	0.00	0	.	.
Syria	1	0.400	0.322	0.478	1.54	0.00	0	.	.
Germany	1	2.100	1.890	2.310	1.29	0.00	0	.	.
Overall	78	0.904	0.850	0.959	100	1.30 × 10^6^	77	**< 0.001**	100.00

^a^ Number study.

^b^ Effect Size: pooled concentration.

^c^ Bold *p* values indicate statistically significant heterogeneity (*p* < 0.05).

Although milk is a beneficial food (Alia et al. 2024; Alshawi 2025), its contamination poses a significant risk to human health (Bali et al. 2024). The higher concentrations of potassium (specifically radioactive K‐40) and cesium (notably radioactive Cs‐137) in milk can be explained by their environmental mobility, biological roles, and transfer mechanisms from feed to milk. Potassium is an essential nutrient for plants, animals, and humans, and is naturally abundant in the environment and in biological systems. K‐40, a naturally occurring radionuclide, is part of this potassium pool (Durusoy and Yildirim 2017). K‐40 accumulates primarily in muscles in humans (Gallagher et al. 1997; Silva, Shen, et al. 2010) and is a natural component of milk due to its biological importance. Cs‐137 is also an artificial radionuclide introduced into the environment mainly through nuclear fallout (Pietrzak‐Fiećko and Smoczyński 2009; Tsugo and Omomo 1963). The concentration of Cs‐137 in milk is directly proportional to the intake of contaminated feed by cows. Studies indicate that approximately 13% of ingested cesium is transferred to milk (Kahn et al. 1965). Grazing on contaminated grass and drinking contaminated water are primary routes by which radionuclides, such as cesium, enter the animal and subsequently the milk (Howard 2021). Cs‐137 levels in milk depend on environmental contamination and cow feed intake, with direct transfer from contaminated grass and water to milk (Howard 2021; Vreman et al. 1989). Cesium concentration in milk can vary regionally and due to metabolic and environmental factors. These factors collectively explain why potassium and cesium concentrations in milk are relatively higher than those of other radionuclides (Pietrzak‐Fiećko and Smoczyński 2009; Kahn et al. 1965).

The meta‐analysis results for Cs‐137 in milk revealed significant variability across countries. The highest concentration was observed in the United Kingdom (288.000 Bq/L; 95% CI: 280.225–295.775), whereas the lowest was in Mali (0.003 Bq/L; 95% CI: 0.003–0.004). Other notable concentrations included Austria (52.466 Bq/L, 95% CI: 34.696–70.236) and South Korea (23.000 Bq/L, 95% CI: 22.434–23.566). The overall pooled concentration was 0.904 Bq/L (95% CI: 0.850–0.959) (Table 1). The United Kingdom exhibited the highest Cs‐137 concentration in milk, which is notably elevated compared to other countries. This high level likely reflects historical nuclear fallout that influences cesium transfer into the food chain. Austria and South Korea showed intermediate concentrations. For Austria, previous studies have documented significant Cs‐137 contamination in milk powder shortly after the Chernobyl accident, with levels decreasing over time but still measurable decades later (Pietrzak‐Fiećko and Smoczyński 2009). South Korea's levels may be influenced by regional fallout and soil‐to‐plant transfer factors. Mali had the lowest concentration, environmental conditions that limit cesium uptake into milk. These disparities are consistent with known patterns of Cs‐137 contamination and other contaminants, which depend heavily on the extent of nuclear fallout deposition, soil characteristics (Pervin et al. 2024; Saeidi et al. 2024), and agricultural practices (Pervin et al. 2024; Saeidi et al. 2024), as well as factors such as feed type and cow grazing behavior (Pietrzak‐Fiećko and Smoczyński 2009). For example, areas with peat soils tend to retain more cesium, greater transfer to milk, as observed in Finland (Barescut et al. 2005). The meta‐analysis determines the significance of these factors in influencing Cs‐137 levels in milk. Continuous monitoring remains crucial, especially in regions with known contamination, to ensure food safety and public health.

The highest concentration of Cs‐134 was reported in the USA (0.400 Bq/L, 95% CI: 0.181–0.618), while Malaysia had the lowest (0.007 Bq/L, 95% CI: 0.006–0.007). Finland also showed a relatively high concentration (0.240 Bq/L, 95% CI: 0.155–0.325). The overall concentration was 0.032 Bq/L (95% CI: 0.025–0.038) (Table 2). The reported data on Cs‐134 concentrations reveal significant geographic variation, with the highest levels observed in the USA and the lowest in Malaysia. The elevated Cs‐134 levels in the USA and Finland could be attributed to their proximity to nuclear testing sites or nuclear power plants, as well as atmospheric fallout patterns. Cs‐134 is a radioactive isotope commonly associated with nuclear fission events, and its presence can indicate recent or historical nuclear activity (Kameník et al. 2013).

**TABLE 2:** Meta‐analysis concentration of Cs‐134 in milk (Bq/L).

Country	NS^a^	ES^b^	Lower	Upper	Weight (%)	Heterogeneity statistic	Degrees of freedom	*p*	*I* ^2^(%)
France	2	0.050	0.044	0.056	10.65	0.00	1	1	0.00
Italy	2	0.040	0.028	0.052	10.54	3.80 × 10˚	1	**0.051** ^c^	73.70
Japan	3	0.033	0.018	0.047	14.88	1.19 × 10¹	2	**0.003**	83.30
Romania	10	0.017	0.015	0.019	54.34	1.27 × 10¹	9	0.177	29.10
Malaysia	1	0.007	0.006	0.007	5.73	0.00	0	.	.
USA	5	0.400	0.181	0.618	3.28	6.94 × 10¹	4	**< 0.001**	94.20
Finland	1	0.240	0.155	0.325	0.57	0.00	0	.	.
Overall	24	0.032	0.025	0.038	100	8.37 × 10²	**23**	**< 0.001**	97.30

^a^ Number study.

^b^ Effect Size: pooled concentration.

^c^ Bold *p* values indicate statistically significant heterogeneity (*p* < 0.05).

I‐131 exhibited extreme variability, with Syria recording the highest concentration (76.000 Bq/L; 95% CI: 68.552–83.448), whereas the United Kingdom had one of the lowest concentrations (0.050 Bq/L; 95% CI: 0.042–0.058). Romania also showed elevated levels (3.229 Bq/L, 95% CI: 2.273–4.185). The overall concentration was 0.301 Bq/L (95% CI: 0.255–0.347) (Table 3). I‐131 concentrations in milk substantial variability across countries following nuclear contamination events. This variability reflects differences in fallout deposition, local environmental conditions, and timing of sampling relative to the radioactive decay of I‐131, which has a half‐life of about 8 days. The high levels in Syria and Romania contrast sharply with the low levels in the United Kingdom, illustrating the heterogeneous impact of radioactive contamination on milk safety across regions (Mărgineanu et al. 2011; Wolff 1959; Köhler et al. 1991; Zvonova et al. 2004; Barescut et al. 2009).

**TABLE 3:** Meta‐analysis concentration of I‐131 in milk (Bq/L).

Country	NS^a^	ES^b^	Lower	Upper	Weight (%)	Heterogeneity statistic	Degrees of freedom	*p*	*I* ^2^(%)
USA	13	0.149	0.106	0.191	48.42	2.12 × 10²	12	**< 0.001** ^c^	94.30
Italy	4	0.365	0.173	0.556	13.86	1.54 × 10²	3	**< 0.001**	98.10
France	1	0.270	0.246	0.294	4.73	0.00	0	.	.
Romania	11	3.229	2.273	4.185	8.14	3.99 × 10²	10	**< 0.001**	97.50
Spain	2	0.441	0.000	0.973	4.86	8.18 × 10˚	1	**0.004**	87.80
UK	1	0.050	0.042	0.058	4.79	0.00	0	.	.
Jordan	1	0.075	0.052	0.098	4.74	0.00	0	.	.
Greece	2	0.786	0.666	0.905	4.54	1.00 × 10−^1^	1	0.752	0.00
Japan	2	0.767	0.000	1.599	5.91	3.72 × 10¹	1	**< 0.001**	97.30
Syria	1	76.000	68.552	83.448	0	0.00	0	.	.
Overall	38	0.301	0.255	0.347	100	1.82 × 10³	37	**< 0.001**	98.00

^a^ Number study.

^b^ Effect Size: pooled concentration.

^c^ Bold *p* values indicate statistically significant heterogeneity (*p* < 0.05).

Pb‐210 concentrations were highest in India (1.080 Bq/L, 95% CI: 0.924–1.236) and lowest in New Zealand (0.004 Bq/L, 95% CI: 0.003–0.006). Tunisia showed a wide range (0.508 Bq/L, 95% CI: 0.000–1.464). The overall concentration was 0.136 Bq/L (95% CI: 0.109–0.163) (Table 4). Pb‐210 concentrations in milk samples vary significantly by country. This variation reflects differences in environmental contamination, agricultural practices, and possibly industrial pollution affecting dairy production in these regions. Pb‐210 is a radioactive isotope that can pose health risks if ingested in significant amounts through food such as milk (Silva, Amaral, et al. 2010; Zhou et al. 2023). Pb‐210 concentrations in milk samples have been studied using various radiometric techniques, primarily alpha spectrometry and gamma spectrometry. The measured concentrations of Pb‐210 in milk are typically low but detectable, reflecting the presence of natural radionuclides transferred from the environment via forage to the milk. Pb‐210 and Po‐210 concentrations in cow's milk have been assessed using alpha spectrometry and calculation methods, showing measurable but generally low activity levels (Boryło et al. 2021). Detection limits for Pb‐210 in food samples, including milk, can be as low as around 28.6 mBq/kg, enabling sensitive quantification of this radionuclide in milk products (Zheng et al. 2024). In milk from areas near former uranium mines, Pb‐210 exhibited some of the highest activity concentrations among the natural radionuclides detected, indicating environmental influence on milk radioactivity (Štrok and Smodiš 2011). Studies on powdered milk samples also confirm the presence of natural radionuclides, including Pb‐210, with activity levels generally below regulatory limits and considered safe for human consumption (Melquiades and Appoloni 2004). Transfer studies indicate that Pb‐210 concentrations in forage are higher than those of 226Ra, whereas milk samples may show relatively higher 226Ra than Pb‐210, suggesting complex transfer dynamics from the environment to milk (Amaral et al. 1988).

**TABLE 4:** Meta‐analysis concentration of Pb‐210 in milk (Bq/L).

Country	NS^a^	ES^b^	Lower	Upper	Weight (%)	Heterogeneity statistic	Degrees of freedom	*p*	*I* ^2^(%)
Czech	1	0.110	0.091	0.129	11.96	0.00	0	.	.
Italy	1	0.013	0.010	0.016	12.74	0.00	0	.	.
Tunisia	2	0.508	0.000	1.464	14.71	1.19 × 10²	1	**< 0.001** ^c^	99.20
French	1	0.120	0.113	0.127	12.64	0.00	0	.	.
New Zealand	1	0.004	0.003	0.006	12.75	0.00	0	.	.
Syria	4	0.690	0.434	0.947	20.27	1.04 × 10²	3	**< 0.001**	97.10
India	1	1.080	0.924	1.236	2.41	0.00	0	.	.
Slovenia	1	0.039	0.029	0.049	12.52	0.00	0	.	.
**Overall**	**12**	**0.136**	**0.109**	**0.163**	**100**	**1.75 × 10³**	**11**	**< 0.001**	**99.40**

^a^ Number study.

^b^ Effect Size: pooled concentration.

^c^ Bold *p* values indicate statistically significant heterogeneity (*p* < 0.05).

K‐40 had the highest concentration in Syria (243.500 Bq/L; 95% CI: 211.984–275.016), whereas Algeria had the lowest (2.349 Bq/L; 95% CI: 2.098–2.601). Nigeria also showed notably high levels (188.114 Bq/L, 95% CI: 0.000–440.751). The overall concentration was 57.494 Bq/L (95% CI: 52.063–62.925) (Table 5). In general, K‐40 is a naturally occurring radionuclide found in milk worldwide, with concentrations influenced by environmental factors such as soil composition and fertilizer use. These variations are important for monitoring natural radioactivity exposure through diet; however, the effective radiation doses from K‐40 in milk are generally low and are not considered a significant health hazard (Sarayegord and Ghiassi 2009). Potassium's mobility in soil and its role as a nutrient for plants drive its uptake into dairy products (Sarayegord and Ghiassi 2009). K‐40 in milk serves as a reliable indicator of environmental potassium distribution, with concentrations reflecting natural geological and agricultural processes rather than anthropogenic contamination. The collected data can be highly valuable for monitoring efforts in particular scenarios, such as the unregulated application of fertilizers and other human‐induced environmental changes. This is because potassium, together with nitrogen and phosphorus, is a key soil nutrient, and the concentration of the radioactive isotope K‐40 in soils is significantly influenced by fertilizer use.

**TABLE 5:** Meta‐analysis concentration of K‐40 in milk (Bq/L).

Country	NS^a^	ES^b^	Lower	Upper	Weight (%)	Heterogeneity statistic	Degrees of freedom	*p*	*I* ^2^(%)
Argentina	1	60	57.712	62.288	2.63	0.00	0	.	.
Singapore	1	25.61	23.635	27.585	2.63	0.00	0	.	.
Vietnam	1	371	360.954	381.046	2.42	0.00	0	.	.
Thailand	1	27.716	25.55	29.882	2.63	0.00	0	.	.
India	1	8.78	2.73	14.83	2.55	0.00	0	.	.
Egypt	3	32.382	0	68.797	7.9	4.03 × 10³	2	**< 0.001** ^**c**^	100.00
Malaysia	3	26.411	17.125	35.696	7.88	2.23 × 10²	2	**< 0.001**	99.10
Mali	1	31.564	30.564	32.564	2.64	0.00	0	.	.
Tunisia	2	48.604	29.985	67.222	5.24	8.67 × 10¹	1	**< 0.001**	98.80
Nigeria	2	188.114	0	440.751	3.83	2.08 × 10²	1	**< 0.001**	99.50
Bosnia	1	42.5	38.752	46.248	2.6	0.00	0	.	.
South Korea	1	43	38.474	47.526	2.59	0.00	0	.	.
Saudi Arabia	3	50.09	39.254	60.927	7.9	5.15 × 10²	2	**< 0.001**	99.60
Serbia	4	76.2	60.303	92.098	10.53	2.59 × 10³	3	**< 0.001**	99.90
Italy	1	70.5	49.055	91.945	1.87	0.00	0	.	.
Syria	1	243.5	211.984	275.016	1.4	0.00	0	.	.
Jordan	1	48.5	47.105	49.895	2.63	0.00	0	.	.
Lebanon	1	45.5	45.41	45.59	2.64	0.00	0	.	.
Bangladesh	1	49.4	45.236	53.564	2.6	0.00	0	.	.
Iraq	2	33.35	9.026	57.674	4.86	1.16 × 10¹	1	**0.001**	91.30
Algeria	1	2.349	2.098	2.601	2.64	0.00	0	.	.
Turkey	1	47	46.43	47.57	2.64	0.00	0	.	.
Japan	2	48.518	48.147	48.889	5.27	2.40 × 10^−1^	1	0.624	0.00
Spain	4	36.113	30.266	41.961	9.5	5.42 × 10˚	3	0.144	44.60
Overall	40	57.494	52.063	62.925	100	1.90 × 10^5^	39	**< 0.001**	100.00

^a^ Number study.

^b^ Effect Size: pooled concentration.

^c^ Bold *p* values indicate statistically significant heterogeneity (*p* < 0.05).

### 3.2 Human Risk Assessment

**FIGURE 2:**
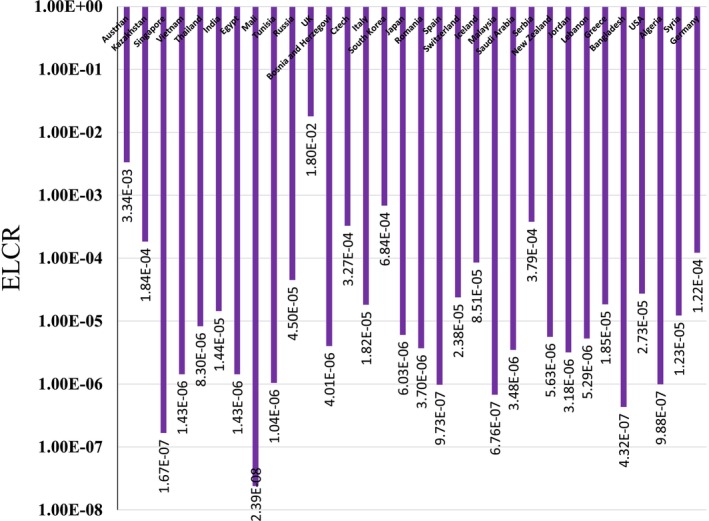
Excess lifetime cancer risk due to Cs‐137 in milk for adults.

The mean CR for adults due to Cs‐137 in the United Kingdom (1.80E‐02), Austria (3.34E‐03), South Korea (6.84E‐04), Serbia (3.79E‐04), the Czech Republic (3.27E‐04), Germany (1.22E‐04), Kazakhstan (1.84E‐04), and Iceland (8.51E‐05) was higher than the acceptable limit (1E‐4) (Figure 2). Cs‐137 contamination in milk is a known consequence of nuclear accidents, with levels varying by region depending on soil type, deposition, and local agricultural practices. For example, studies have shown that characteristics. Soil characteristics can influence Cs‐137 levels in milk. Soil characteristics can influence Cs‐137 levels in milk and can persist for years after contamination events (Pietrzak‐Fiećko and Smoczyński 2009). The United Kingdom and Austria have experienced relatively high intake rates of contaminated milk, contributing to the higher radiation doses observed (Nuclear Energy Agency 1987). The United Kingdom has the most severe Cs‐137 contamination among the studied countries, with doses that far exceed safety thresholds, followed by Austria, which has moderate contamination. The high Cs‐137 levels in the United Kingdom are due to higher environmental concentrations of this radionuclide in these regions (Pietrzak‐Fiećko and Smoczyński 2009).

**FIGURE 3:**
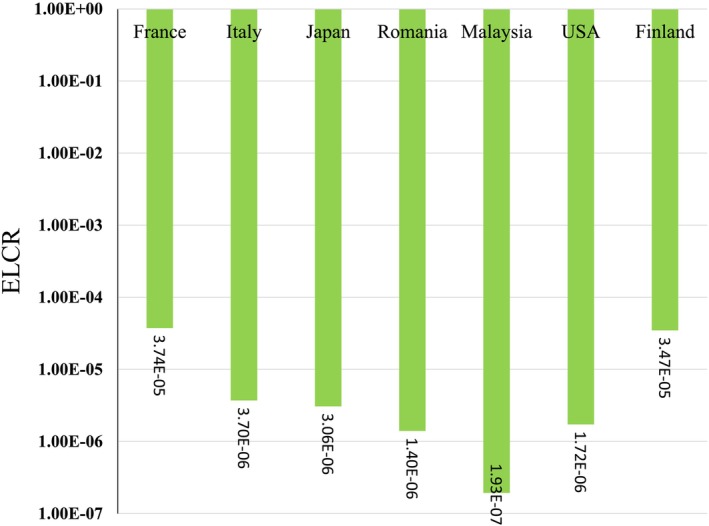
Excess lifetime cancer risk due to ^134^Cs in milk for adults.

The mean CR for adults due to Cs‐134 in Finland (3.47E‐05) and France (3.74E‐05) remained below the 1.00E‐04 criterion. Therefore, none of the countries listed exceeded the specified risk limit (Figure 3). The assessment of Cs‐134 concentrations in milk from Northern Europe and North America reveals a consistent regional pattern of low but detectable radiation exposure, with Finland and the United States showing the highest values. These findings indicate marginally elevated radiation levels in these northern latitudes but remain well within safe exposure limits for human health. Overall, although the highest Cs‐134 concentrations in milk are found in these northern regions, adult exposure levels remain marginal and safe. Continuous monitoring remains essential to track any future changes; however, current data suggest that radiation exposure through milk consumption in these areas does not pose a health threat. This underscores the effectiveness of food safety regulations and environmental surveillance in managing radioactive contamination in the food chain.

**FIGURE 4:**
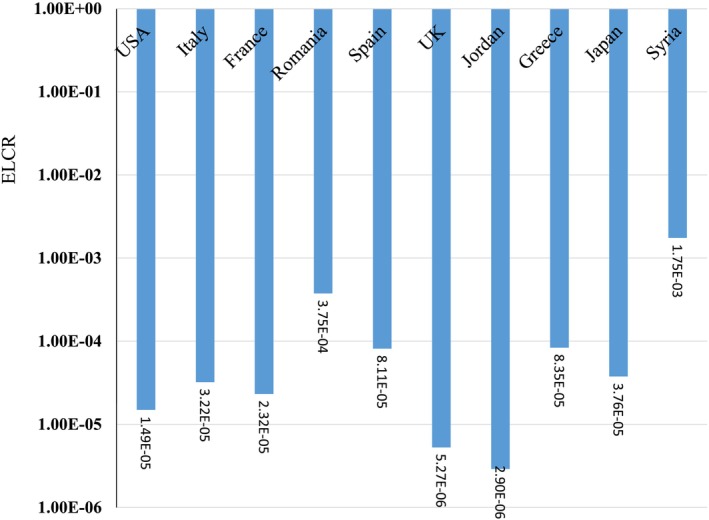
Excess lifetime cancer risk due to I‐131 in milk for adults.

The mean CR for adults due to I‐131 in Syria (1.75E‐03), Romania (3.75E‐04), Greece (8.35E‐05), and Spain (8.11E‐05) was higher than the acceptable limit (1E‐4) (Figure 4). The reported I‐131 exposure level exceeds the typical safety threshold. This indicates a significant health risk, especially for Syrian people. Children's thyroid glands are more sensitive to radiation than those of adults, and their metabolic and physiological differences result in higher committed effective doses per unit intake of I‐131. For example, the committed effective dose coefficients for I‐131 are substantially larger for infants and young children than for adults, reflecting their heightened radio sensitivity and increased risk of thyroid cancer (Saad et al. 2006). I‐131 accumulates in milk because dairy cows ingest contaminated pasture, transferring the radioactive iodine into their milk. This “milk pathway” is a well‐known route of exposure, historically linked to increased thyroid doses in children after nuclear fallout events (Ortmeyer and Makhijani 1997). Children consume more milk relative to their body size, and their smaller, developing thyroids absorb more I‐131, compounding the risk (Ortmeyer and Makhijani 1997). The quadrupling of the safety threshold for children in Syria is alarming because it suggests a high likelihood of increased thyroid cancer risk and other radiation‐induced health effects in the young population. Preventative measures, such as administering potassium iodide, can block radioactive iodine uptake by the thyroid and reduce health risks; however, such interventions must be timely and adequately implemented. The elevated I‐131 doses from milk in Syria represent a serious public health concern, especially for children, due to their increased radio sensitivity and the critical role of milk as a contamination vector. This situation requires urgent monitoring, protective measures, and public health interventions to mitigate the heightened risk of radiation exposure to vulnerable populations.

**FIGURE 5:**
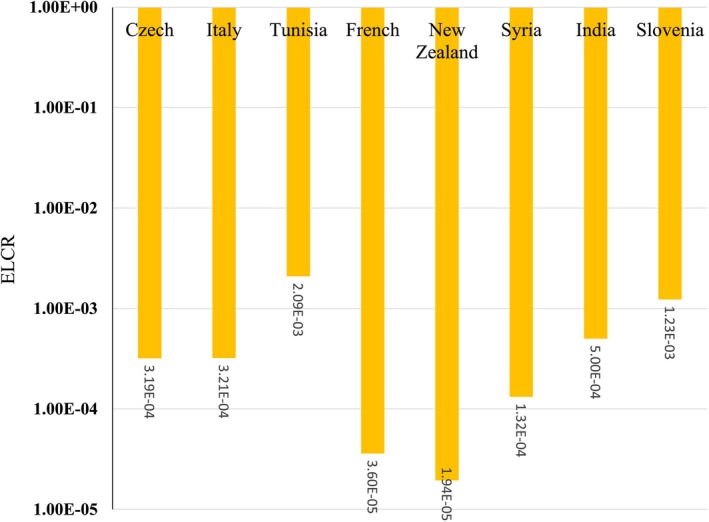
Excess lifetime cancer risk due to Pb‐210 in milk for adults.

The mean CR for adults due to Pb‐210 in Tunisia (2.09E‐03), Slovenia (1.23E‐03), India (5.00E‐04), the Czech Republic (3.19E‐04), Italy (3.21E‐04), and Syria (1.32E‐04) was higher than the acceptable limit (1E‐4) (Figure 5). In Tunisia, although the adult dose is lower than in India, it still exceeds, underscoring the need for regulatory measures and ongoing monitoring to protect public health. Natural radioactivity in milk generally comes from radionuclides like Ra‐226, Th‐232, and K‐40 (Biçer and Cetinkaya 2023). Still, typical milk ingestion doses are typically below worldwide averages reported by UNSCEAR (United Nations Scientific Committee on the Effects of Atomic Radiation) and other studies (Biçer and Cetinkaya 2023). For example, one study in Turkey found milk ingestion doses below global averages (Duong et al. 2021), indicating that contamination levels in India and Tunisia are unusually high. The Pb‐210 contamination levels in milk in India and Tunisia that exceed safety limits pose a radiological health risk, particularly to children in India, and require immediate regulatory and public health responses to reduce exposure and protect vulnerable populations. Health authorities in India and Tunisia should prioritize establishing and enforcing maximum permissible limits for Pb‐210 and other radionuclides in milk, in accordance with international guidelines, such as those from Codex Alimentarius and UNSCEAR (Alimentarius 2011).

**FIGURE 6:**
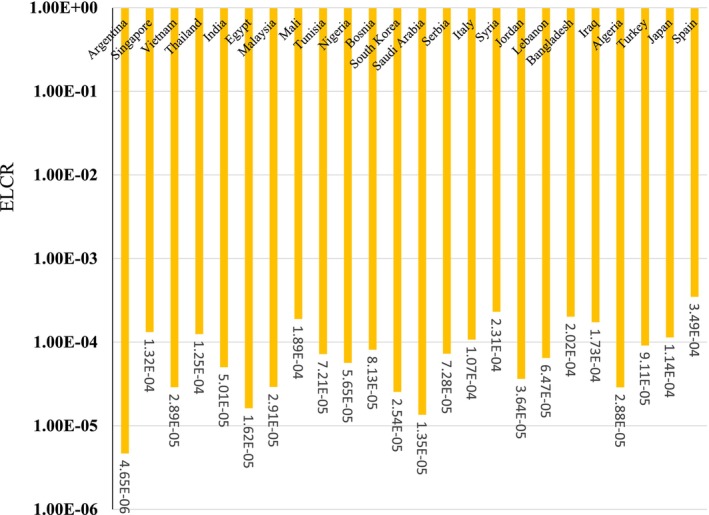
Excess lifetime cancer risk due to K‐40 in milk for adults.

The mean CR for adults due to K‐40 in Spain (3.49E‐04), Syria (2.31E‐04), Bangladesh (2.02E‐04), Mali (1.89E‐04), Iraq (1.73E‐04), Singapore (1.32E‐04), Thailand (1.25E‐04), Japan (1.14E‐04), Italy (1.07E‐04), and Turkey (9.11E‐05) was higher than the acceptable limit (1E‐4) (Figure 6). The International Atomic Energy Agency (IAEA) and Codex Alimentarius have established radiological criteria to ensure that doses from radionuclides in foodstuffs remain within safe limits, typically aiming to keep individual effective doses below 1 mSv/year to minimize health risks (International Atomic Energy Agency 2005). The exceptionally high reading in Vietnam exceeds these recommended limits, indicating the need for further investigation and verification through additional testing. Such elevated levels could stem from environmental factors, contamination during milk production, or measurement errors. Verification is crucial for ruling out anomalies and accurately assessing potential sources of contamination. In comparison, the other countries listed also exceed the 1 mSv/year guideline, but to a lesser extent. This widespread occurrence suggests a potential regional or global issue related to the presence of natural or anthropogenic radionuclides in dairy products, which may necessitate coordinated monitoring and regulatory responses. Given the importance of milk as a dietary staple, especially for vulnerable populations such as children, ensuring its safety from radioactive contamination is crucial. Regular screening using sensitive diagnostic technologies and adherence to international safety standards are essential to maintain consumer confidence.

### 3.3 Method of Detection Statistics

**FIGURE 7:**
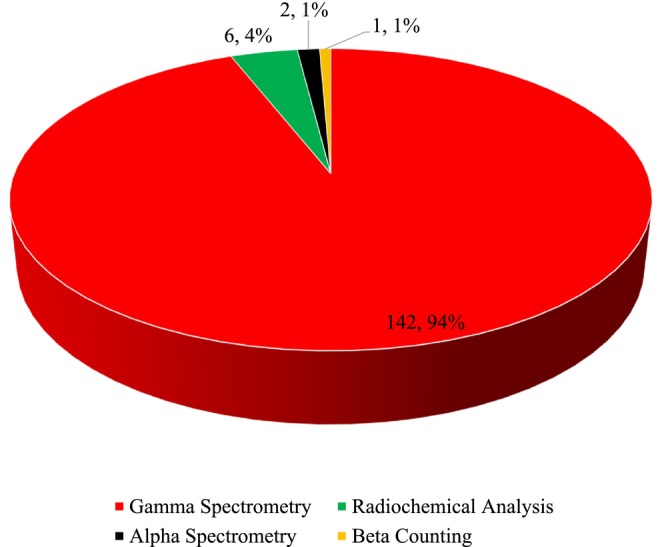
The number and percentage of method of detection radionuclides in milk.

The analysis of 151 methodological entries reveals an overwhelming predominance of Gamma Spectrometry, which was employed in 142 instances (94%), establishing it as the cornerstone technique for radionuclide assessment in milk (Figure 7). This reliance is fundamentally due to the method's direct, nondestructive nature, which allows rapid screening of samples with minimal preparation, and its superior multinuclide capability, enabling the simultaneous quantification of key gamma‐emitting fission products, such as Cs‐137 and I‐131. In contrast, the other techniques are applied selectively for specific analytical challenges; Radiochemical Analysis, used 6 times (4%), is indispensable for isolating pure beta or alpha emitters such as St‐90, which are undetectable by gamma spectrometry, while the infrequent use of Alpha Spectrometry (1.3%) and Beta Counting (0.7%) typically represents the final measurement step following such complex chemical separations. Consequently, this methodological distribution demonstrates a highly efficient monitoring strategy: Gamma Spectrometry serves as the primary tool for broad surveillance against the most prevalent environmental contaminants, whereas the more labor‐intensive radiochemical methods are reserved for a comprehensive risk assessment of specific radionuclides that pose a targeted analytical challenge to foodstuffs (Melquiades and Appoloni 2004; Abbasi 2013; Pervin et al. 2024). In general, the widespread use of gamma spectrometry in milk radioactivity analysis reflects its superior resolution, sensitivity, and reliability, which are essential for ensuring food safety and public health. Other methods serve as complementary or alternative approaches, depending on the specific analytical requirements and resource availability (Saeidi et al. 2024).

## 4 Conclusion

This comprehensive analysis of radionuclide concentrations in milk across various countries highlights the complex interplay among environmental, agricultural, and historical factors that influence food safety. Among radionuclides, Cs‐137 and I‐131 exhibited the most significant variability, with the highest levels detected in the United Kingdom and Syria, respectively. These elevated concentrations are closely linked to past nuclear fallout events and local environmental conditions, underscoring the long‐term impact of such incidents on the food chain. The mean CR, as Cs‐137 exceeded the threshold in the United Kingdom, Austria, South Korea, Serbia, the Czech Republic, Germany, Kazakhstan, and Iceland. Similarly, elevated risks were identified for K‐40 in Spain, Syria, Bangladesh, Mali, Iraq, Singapore, Thailand, Japan, Italy, and Turkey; for I‐131 in Syria, Romania, Greece, and Spain; and for Pb‐210 in Tunisia, Slovenia, India, the Czech Republic, Italy, and Syria. In contrast, the mean CR for Cs‐134 in Finland and France remained below the criterion. Overall, the results emphasize the necessity for ongoing monitoring of radionuclide levels in dairy products, particularly in regions with a history of nuclear activity or environmental contamination. Maintaining rigorous food safety standards and implementing timely protective measures are essential to safeguard public health, particularly for vulnerable groups.

## Author Contributions

Study design and search were conducted by Yadolah Fakhri and Mansour Sarafraz. Data extraction by Yadolah Fakhri, Zeinab Gholami, Olga A. Malanova, and Mansour Sarafraz. Manuscript preparation was conducted by Yadolah Fakhri, Zeinab Gholami, Olga A. Malanova, Farshid Soleimani, Seid Kamal Ghadiri, Mohammadreza Gharepour, Moayed Adiban, and Mansour Sarafraz.

## Funding

This research was financially supported by Shahroud University of Medical Sciences under grant number 14030041 and conducted under Ethics Code (IR.SHMU.REC.1403.095).

## Conflicts of Interest

The authors declare no conflicts of interest.

## Supporting information


**Appendix S1:** Main characteristic included in our study (Bq/L).
**Appendix S2:**. Consumption rate of milk based on country [67].
**Appendix S3:** Radionuclide‐specific cancer risk factors per Bq ingested [68‐70].
**Appendix S4:** The MCS model for determine CR of Cs‐137 in milk.
**Appendix S5:** The MCS model for determine CR of Cs‐134 in milk.
**Appendix S6:** The MCS model for determine CR of K‐40 in milk.
**Appendix S7:** The MCS model for determine CR of I‐131 in milk.
**Appendix S8:** The MCS model for determine CR of Pb‐210 in milk.

## Data Availability

The data that supports the findings of this study are available in the Supporting Information of this article.
