# Supplementary material for: Radionuclides in Milk: A Global Systematic Review and Meta‐Analysis and Probabilistic Human Risk Assessment
Source: Food Sci Nutr. 2026 May 20;14(5):e71770. doi: 10.1002/fsn3.71770 (PMC13239174; doi:10.1002/fsn3.71770)
Supplement: Supplementary file 1 — Appendix S1: Main characteristic included in our study (Bq/L). Appendix S2:. Consumption rate of milk based on country [67]. Appendix S3: Radionuclide‐specific cancer risk factors per Bq ingested [68‐70]. Appendix S4: The MCS model for determine CR of Cs‐137 in milk. Appendix S5: The MCS model for determine CR of Cs‐134 in milk. Appendix S6: The MCS model for determine CR of K‐40 in milk. Appendix S7: The MCS model for determine CR of I‐131 in milk. Appendix S8: The MCS model for determine CR of Pb‐210 in milk. [file FSN3-14-e71770-s001.docx]

**Supplementary appendix 1.** Main characteristic included in our study (Bq/L)

| **Country** | **Type of milk** | **Sample number** | **Type of radioactive element** | **Mean** | **SD^[[1]](#footnote-1)^** | **Method of detection** | **Reference** |
| --- | --- | --- | --- | --- | --- | --- | --- |
| Austria | Cow milk | 7 | Cs-137 | 95.300 | 6.900 | Gamma Spectrometry | [1] |
| Austria | Cow milk | 9 | Cs-137 | 58.800 | 12.800 | Gamma Spectrometry | [1] |
| Austria | Cow milk | 8 | Cs-137 | 20.100 | 6.200 | Gamma Spectrometry | [1] |
| Austria | Cow milk | 10 | Cs-137 | 139.300 | 15.300 | Gamma Spectrometry | [1] |
| Austria | Cow milk | 9 | Cs-137 | 70.800 | 10.400 | Gamma Spectrometry | [1] |
| Austria | Cow milk | 9 | Cs-137 | 6.800 | 2.300 | Gamma Spectrometry | [1] |
| Austria | Cow milk | 9 | Cs-137 | 16.000 | 2.800 | Gamma Spectrometry | [1] |
| Austria | Cow milk | 8 | Cs-137 | 26.600 | 3.700 | Gamma Spectrometry | [1] |
| Austria | Cow milk | 11 | Cs-137 | 41.400 | 14.600 | Gamma Spectrometry | [1] |
| Kazakhstan | Milk | 4 | Cs-137 | 0.050 | 0.009 | Gamma Spectrometry | [2] |
| Argentina | Milk | 31 | K-40 | 60.000 | 6.500 | Gamma Spectrometry | [3] |
| Czech | Milk | 4 | Pb-210 | 0.110 | 0.020 | Radiochemical Analysis | [4] |
| USA | Milk | 6 | I-131 | 0.100 | 0.018 | Gamma Spectrometry | [5] |
| Singapore | Powder milk | 16 | K-40 | 25.610 | 4.030 | Gamma Spectrometry | [6] |
| Singapore | Powder milk | 16 | Cs-137 | 0.010 | 0.008 | Gamma Spectrometry | [6] |
| Italy | Milk | 10 | Pb-210 | 0.013 | 0.005 | Alpha Spectrometry | [7] |
| Vietnam | Milk | 10 | Cs-137 | 0.130 | 0.060 | Gamma Spectrometry | [8] |
| Vietnam | Milk | 11 | K-40 | 371.000 | 17.000 | Gamma Spectrometry | [8] |
| Thailand | Powder milk | 9 | K-40 | 27.716 | 3.315 | Gamma Spectrometry | [9] |
| Thailand | Powder milk | 9 | Cs-137 | 0.637 | 0.096 | Gamma Spectrometry | [9] |
| India | Milk | 14 | K-40 | 8.780 | 11.550 | Gamma Spectrometry | [10] |
| India | Milk | 14 | Cs-137 | 0.240 | 0.440 | Gamma Spectrometry | [10] |
| Egypt | Powder milk | 16 | Cs-137 | 0.055 | 0.014 | Gamma Spectrometry | [11] |
| Egypt | Powder milk | 17 | K-40 | 62.140 | 3.250 | Gamma Spectrometry | [11] |
| Malaysia | Powder milk | 26 | K-40 | 27.690 | 0.272 | Gamma Spectrometry | [12] |
| Mali | Powder milk | 3 | Cs-137 | 0.003 | 0.001 | Gamma Spectrometry | [13] |
| Mali | Powder milk | 3 | K-40 | 31.564 | 0.884 | Gamma Spectrometry | [13] |
| Tunisia | Milk | 5 | K-40 | 58.000 | 0.750 | Gamma Spectrometry | [14] |
| Tunisia | Milk | 5 | Pb-210 | 1.000 | 0.200 | Gamma Spectrometry | [14] |
| Tunisia | Milk | 5 | Cs-137 | 0.030 | 0.005 | Gamma Spectrometry | [14] |
| Tunisia | Milk | 5 | K-40 | 39.000 | 4.500 | Gamma Spectrometry | [14] |
| Tunisia | Milk | 5 | Pb-210 | 0.025 | 0.005 | Gamma Spectrometry | [14] |
| Tunisia | Milk | 5 | Cs-137 | 0.015 | 0.003 | Gamma Spectrometry | [14] |
| Russia | Whole milk | 98 | Cs-137 | 2.700 | 2.000 | Gamma Spectrometry | [15] |
| Russia | Whole milk | 98 | Cs-137 | 0.240 | 0.030 | Gamma Spectrometry | [15] |
| Russia | Powder milk | 98 | Cs-137 | 0.936 | 0.065 | Gamma Spectrometry | [15] |
| Russia | Powder milk | 98 | Cs-137 | 0.026 | 0.004 | Gamma Spectrometry | [15] |
| Nigeria | Milk | 11 | K-40 | 317.600 | 58.500 | Gamma Spectrometry | [16] |
| Nigeria | Powder milk | 10 | K-40 | 59.800 | 9.451 | Gamma Spectrometry | [16] |
| Kazakhstan | Cow milk | 50 | Cs-137 | 5.800 | 0.130 | Gamma Spectrometry | [17] |
| UK | Milk | 977 | Cs-137 | 288.000 | 124.000 | Gamma Spectrometry | [18] |
| Kazakhstan | Milk | 3 | Cs-137 | 1.800 | 0.600 | Gamma Spectrometry | [19] |
| Bosnia and Herzegovina | Milk | 16 | Cs-137 | 0.070 | 0.013 | Gamma Spectrometry | [20] |
| Bosnia and Herzegovina | Milk | 16 | K-40 | 42.500 | 7.650 | Gamma Spectrometry | [20] |
| Czech | Milk | 5350 | Cs-137 | 6.010 | 2.995 | Gamma Spectrometry | [21] |
| Italy | Sheep milk | 7 | I-131 | 2.450 | 1.225 | Gamma Spectrometry | [22] |
| Italy | Sheep milk | 7 | Cs-137 | 0.330 | 0.070 | Gamma Spectrometry | [22] |
| South Korea | Milk | 12 | K-40 | 43.000 | 8.000 | Gamma Spectrometry | [23] |
| South Korea | Milk | 12 | Cs-137 | 23.000 | 1.000 | Gamma Spectrometry | [23] |
| France | Cow milk | 5 | I-131 | 0.270 | 0.028 | Gamma Spectrometry | [24] |
| France | Cow milk | 5 | Cs-134 | 0.050 | 0.009 | Gamma Spectrometry | [24] |
| France | Cow milk | 5 | Cs-134 | 0.050 | 0.009 | Gamma Spectrometry | [24] |
| Italy | Cow milk | 11 | I-131 | 0.135 | 0.056 | Gamma Spectrometry | [25] |
| Italy | Cow milk | 11 | Cs-137 | 0.547 | 0.125 | Gamma Spectrometry | [25] |
| Italy | Cow milk | 11 | Cs-134 | 0.046 | 0.012 | Gamma Spectrometry | [25] |
| Italy | Goat milk | 11 | I-131 | 0.118 | 0.045 | Gamma Spectrometry | [25] |
| Italy | Goat milk | 11 | Cs-137 | 0.441 | 0.122 | Gamma Spectrometry | [25] |
| Italy | Goat milk | 11 | Cs-134 | 0.034 | 0.017 | Gamma Spectrometry | [25] |
| Japan | Milk | 7 | Cs-134 | 0.022 | 0.003 | Gamma Spectrometry | [26] |
| Japan | Milk | 7 | Cs-137 | 0.073 | 0.010 | Gamma Spectrometry | [26] |
| Romania | Sheep milk | 1 | I-131 | 9.220 | 0.950 | Gamma Spectrometry | [27] |
| Romania | Sheep milk | 1 | Cs-134 | 0.016 | 0.003 | Gamma Spectrometry | [27] |
| Romania | Sheep milk | 1 | Cs-137 | 0.020 | 0.004 | Gamma Spectrometry | [27] |
| Romania | Sheep milk | 1 | I-131 | 4.010 | 0.700 | Gamma Spectrometry | [27] |
| Romania | Sheep milk | 1 | Cs-134 | 0.016 | 0.003 | Gamma Spectrometry | [27] |
| Romania | Sheep milk | 1 | Cs-137 | 0.039 | 0.007 | Gamma Spectrometry | [27] |
| Romania | Sheep milk | 1 | I-131 | 3.800 | 0.690 | Gamma Spectrometry | [27] |
| Romania | Sheep milk | 1 | Cs-134 | 0.026 | 0.005 | Gamma Spectrometry | [27] |
| Romania | Sheep milk | 1 | Cs-137 | 0.030 | 0.005 | Gamma Spectrometry | [27] |
| Romania | Sheep milk | 1 | I-131 | 3.700 | 0.660 | Gamma Spectrometry | [27] |
| Romania | Sheep milk | 1 | Cs-134 | 0.026 | 0.005 | Gamma Spectrometry | [27] |
| Romania | Sheep milk | 1 | Cs-137 | 0.030 | 0.005 | Gamma Spectrometry | [27] |
| Romania | Sheep milk | 1 | I-131 | 3.800 | 0.640 | Gamma Spectrometry | [27] |
| Romania | Sheep milk | 1 | Cs-134 | 0.026 | 0.005 | Gamma Spectrometry | [27] |
| Romania | Sheep milk | 1 | Cs-137 | 0.030 | 0.005 | Gamma Spectrometry | [27] |
| Romania | Sheep milk | 1 | I-131 | 3.210 | 0.710 | Gamma Spectrometry | [27] |
| Romania | Sheep milk | 1 | Cs-134 | 0.016 | 0.003 | Gamma Spectrometry | [27] |
| Romania | Sheep milk | 1 | Cs-137 | 0.020 | 0.004 | Gamma Spectrometry | [27] |
| Romania | Sheep milk | 1 | I-131 | 2.350 | 0.470 | Radiochemical Analysis | [27] |
| Romania | Sheep milk | 1 | Cs-134 | 0.016 | 0.003 | Gamma Spectrometry | [27] |
| Romania | Sheep milk | 1 | Cs-137 | 0.020 | 0.004 | Gamma Spectrometry | [27] |
| Romania | Sheep milk | 1 | I-131 | 1.920 | 0.420 | Gamma Spectrometry | [27] |
| Romania | Sheep milk | 1 | Cs-134 | 0.016 | 0.003 | Gamma Spectrometry | [27] |
| Romania | Sheep milk | 1 | Cs-137 | 0.020 | 0.004 | Gamma Spectrometry | [27] |
| Romania | Cow milk | 1 | I-131 | 0.031 | 0.006 | Gamma Spectrometry | [27] |
| Romania | Cow milk | 1 | Cs-134 | 0.015 | 0.003 | Gamma Spectrometry | [27] |
| Romania | Cow milk | 1 | Cs-137 | 0.017 | 0.003 | Gamma Spectrometry | [27] |
| Romania | Cow milk | 1 | I-131 | 0.370 | 0.130 | Gamma Spectrometry | [27] |
| Romania | Sheep milk | 1 | I-131 | 0.015 | 0.003 | Gamma Spectrometry | [27] |
| Romania | Sheep milk | 1 | Cs-137 | 0.017 | 0.003 | Gamma Spectrometry | [27] |
| Spain | Cow milk | 1 | Cs-137 | 0.195 | 0.063 | Gamma Spectrometry | [28] |
| Spain | Goat milk | 1 | Cs-137 | 0.740 | 0.180 | Gamma Spectrometry | [28] |
| Spain | Goat milk | 1 | K-40 | 0.090 | 0.030 | Gamma Spectrometry | [28] |
| Switzerland | Milk | 4 | Cs-137 | 0.405 | 0.193 | Gamma Spectrometry | [29] |
| Iceland | Cow milk | 216 | Cs-134 | 1.270 | 0.020 | Gamma Spectrometry | [30] |
| Malaysia | Powder milk | 120 | Pb-210 | 38.220 | 9.490 | Radiochemical Analysis | [31] |
| Malaysia | Powder milk | 120 | K-40 | 0.036 | 0.031 | Gamma Spectrometry | [31] |
| Malaysia | Powder milk | 120 | Cs-137 | 0.007 | 0.003 | Radiochemical Analysis | [31] |
| French | Milk | 700 | K-40 | 0.120 | 0.099 | Radiochemical Analysis | [32] |
| Malaysia | Powder milk | 28 | K-40 | 12.883 | 9.035 | Gamma Spectrometry | [33] |
| Malaysia | Powder milk | 28 | Cs-137 | 0.035 | 0.025 | Gamma Spectrometry | [33] |
| Saudi Arabia | Powder milk | 5 | K-40 | 48.357 | 0.478 | Gamma Spectrometry | [34] |
| Saudi Arabia | Milk | 13 | Cs-137 | 65.000 | 4.000 | Gamma Spectrometry | [35] |
| Saudi Arabia | Milk | 13 | K-40 | 0.120 | 0.030 | Gamma Spectrometry | [35] |
| Saudi Arabia | Powder milk | 33 | Cs-137 | 37.050 | 3.770 | Gamma Spectrometry | [35] |
| Saudi Arabia | Powder milk | 33 | K-40 | 0.049 | 0.005 | Gamma Spectrometry | [35] |
| Serbia | Cow milk | 40 | Cs-137 | 70.370 | 2.200 | Gamma Spectrometry | [36] |
| Serbia | Cow milk | 40 | K-40 | 3.500 | 1.700 | Gamma Spectrometry | [36] |
| Serbia | Sheep milk | 30 | Cs-137 | 73.760 | 2.430 | Gamma Spectrometry | [36] |
| Serbia | Sheep milk | 30 | K-40 | 11.630 | 0.960 | Gamma Spectrometry | [36] |
| Serbia | Goat milk | 20 | Cs-137 | 108.900 | 3.500 | Gamma Spectrometry | [36] |
| Serbia | Goat milk | 20 | Cs-137 | 12.100 | 6.000 | Gamma Spectrometry | [36] |
| Serbia | Cow milk | 4 | Pb-210 | 51.750 | 2.000 | Gamma Spectrometry | [37] |
| Serbia | Cow milk | 4 | K-40 | 0.150 | 0.027 | Gamma Spectrometry | [37] |
| New Zealand | Milk | 2 | Pb-210 | 0.075 | 0.003 | Gamma Spectrometry | [38] |
| New Zealand | Milk | 1 | Pb-210 | 0.005 | 0.001 | Gamma Spectrometry | [38] |
| Italy | Milk | 10 | K-40 | 70.500 | 34.600 | Gamma Spectrometry | [39] |
| Syria | Milk | 9 | Cs-137 | 3.400 | 1.200 | Gamma Spectrometry | [40] |
| Syria | Milk | 9 | I-131 | 3.050 | 1.375 | Gamma Spectrometry | [40] |
| Syria | Milk | 55 | K-40 | 243.500 | 119.250 | Gamma Spectrometry | [40] |
| Romania | Milk | 20 | I-131 | 0.350 | 0.010 | Gamma Spectrometry | [41] |
| UK | Cow milk | 5 | Cs-137 | 0.050 | 0.009 | Gamma Spectrometry | [42] |
| Jordan | Milk | 10 | Cs-137 | 48.500 | 2.250 | Gamma Spectrometry | [43] |
| Jordan | Milk | 10 | K-40 | 0.075 | 0.038 | Gamma Spectrometry | [43] |
| Jordan | Milk | 10 | I-131 | 0.090 | 0.045 | Gamma Spectrometry | [43] |
| Lebanon | Powder milk | 977 | Cs-137 | 0.137 | 0.009 | Gamma Spectrometry | [44] |
| Lebanon | Powder milk | 977 | K-40 | 45.500 | 1.430 | Gamma Spectrometry | [44] |
| Greece | Cow milk | 2 | Cs-137 | 0.780 | 0.090 | Gamma Spectrometry | [45] |
| Greece | Cow milk | 2 | I-131 | 0.120 | 0.028 | Gamma Spectrometry | [45] |
| Bangladesh | Cow milk | 20 | I-131 | 49.400 | 9.500 | Gamma Spectrometry | [46] |
| Bangladesh | Cow milk | 20 | I-131 | 0.050 | 0.030 | Gamma Spectrometry | [46] |
| USA | Powder milk | 1 | I-131 | 0.330 | 0.059 | Gamma Spectrometry | [47] |
| USA | Powder milk | 1 | I-131 | 0.120 | 0.022 | Gamma Spectrometry | [47] |
| USA | Powder milk | 1 | Cs-134 | 0.150 | 0.027 | Gamma Spectrometry | [47] |
| USA | Powder milk | 1 | I-131 | 0.110 | 0.020 | Gamma Spectrometry | [47] |
| USA | Powder milk | 1 | Cs-134 | 0.140 | 0.025 | Gamma Spectrometry | [47] |
| USA | Powder milk | 1 | Cs-137 | 0.090 | 0.016 | Gamma Spectrometry | [47] |
| USA | Powder milk | 1 | I-131 | 2.900 | 0.522 | Gamma Spectrometry | [47] |
| USA | Powder milk | 1 | Cs-134 | 0.400 | 0.072 | Gamma Spectrometry | [47] |
| USA | Powder milk | 1 | Cs-137 | 0.410 | 0.074 | Gamma Spectrometry | [47] |
| USA | Powder milk | 1 | Cs-134 | 1.490 | 0.268 | Gamma Spectrometry | [47] |
| USA | Powder milk | 1 | Cs-137 | 0.360 | 0.065 | Gamma Spectrometry | [47] |
| USA | Powder milk | 1 | I-131 | 0.480 | 0.086 | Gamma Spectrometry | [47] |
| USA | Powder milk | 1 | Cs-134 | 0.410 | 0.074 | Gamma Spectrometry | [47] |
| USA | Powder milk | 1 | Cs-137 | 0.370 | 0.067 | Gamma Spectrometry | [47] |
| USA | Powder milk | 1 | I-131 | 0.670 | 0.121 | Radiochemical Analysis | [47] |
| USA | Powder milk | 1 | I-131 | 0.890 | 0.160 | Gamma Spectrometry | [47] |
| USA | Powder milk | 1 | I-131 | 0.700 | 0.126 | Gamma Spectrometry | [47] |
| USA | Powder milk | 1 | I-131 | 0.110 | 0.020 | Gamma Spectrometry | [47] |
| USA | Powder milk | 1 | Cs-137 | 0.150 | 0.027 | Gamma Spectrometry | [47] |
| USA | Powder milk | 1 | I-131 | 0.030 | 0.005 | Gamma Spectrometry | [47] |
| USA | Powder milk | 1 | Cs-137 | 0.080 | 0.014 | Gamma Spectrometry | [47] |
| Greece | Sheep milk | 1 | Cs-134 | 0.600 | 0.120 | Gamma Spectrometry | [48] |
| Greece | Sheep milk | 4 | I-131 | 0.850 | 0.425 | Gamma Spectrometry | [48] |
| Japan | Milk | 1 | Cs-137 | 0.092 | 0.026 | Gamma Spectrometry | [49] |
| Japan | Milk | 1 | Cs-134 | 0.040 | 0.007 | Beta Counting | [49] |
| Japan | Milk | 1 | I-131 | 1.200 | 0.130 | Gamma Spectrometry | [49] |
| Japan | Milk | 1 | K-40 | 0.150 | 0.030 | Gamma Spectrometry | [49] |
| Japan | Milk | 1 | K-40 | 0.040 | 0.007 | Gamma Spectrometry | [49] |
| Japan | Milk | 1 | K-40 | 0.350 | 0.050 | Gamma Spectrometry | [49] |
| Iraq | Powder milk | 63 | K-40 | 21.956 | 5.114 | Gamma Spectrometry | [50] |
| Egypt | Powder milk | 1 | Cs-137 | 28.874 | 1.032 | Gamma Spectrometry | [51] |
| Egypt | Powder milk | 1 | Cs-137 | 6.143 | 0.419 | Gamma Spectrometry | [51] |
| Algeria | Powder milk | 12 | I-131 | 2.349 | 0.445 | Gamma Spectrometry | [52] |
| Algeria | Powder milk | 12 | Cs-137 | 0.023 | 0.008 | Gamma Spectrometry | [52] |
| Kazakhstan | Milk | 20 | Pb-210 | 0.480 | 0.088 | Alpha Spectrometry | [53] |
| Syria | Camel milk | 25 | Pb-210 | 76.000 | 19.000 | Gamma Spectrometry | [54] |
| Syria | Camel milk | 25 | I-131 | 0.400 | 0.200 | Gamma Spectrometry | [54] |
| Syria | Camel milk | 25 | Pb-210 | 0.250 | 0.100 | Gamma Spectrometry | [54] |
| Syria | Camel milk | 25 | K-40 | 0.200 | 0.100 | Gamma Spectrometry | [54] |
| Italy | Fresh goat and cow milk | 55 | Cs-137 | 0.600 | 0.300 | Gamma Spectrometry | [55] |
| India | Cow milk | 60 | K-40 | 1.080 | 0.615 | Gamma Spectrometry | [56] |
| Turkey | Milk | 20 | Cs-137 | 47.000 | 1.300 | Gamma Spectrometry | [57] |
| Germany | Milk | 14 | Cs-137 | 2.100 | 0.400 | Gamma Spectrometry | [58] |
| Iraq | Powder milk | 4 | K-40 | 46.867 | 14.605 | Gamma Spectrometry | [59] |
| Kazakhstan | Milk | 105 | Cs-137 | 8.600 | 0.160 | Gamma Spectrometry | [60] |
| Japan | Milk and produce | 224 | K-40 | 0.022 | 0.012 | Gamma Spectrometry | [61] |
| Japan | Milk and produce | 224 | I-131 | 49.000 | 15.000 | Radiochemical Analysis | [61] |
| Japan | Milk and produce | 42 | Cs-137 | 0.730 | 0.140 | Gamma Spectrometry | [61] |
| Japan | Milk and produce | 42 | Cs-134 | 48.500 | 1.250 | Gamma Spectrometry | [61] |
| Romania | Sheep milk | 1 | Cs-137 | 5.200 | 0.500 | Gamma Spectrometry | [62] |
| Italy | Milk | 27 | K-40 | 0.085 | 0.063 | Alpha Spectrometry | [63] |
| Finland | Milk | 1 | Cs-137 | 0.240 | 0.043 | Gamma Spectrometry | [64] |
| Spain | Sheep milk | 6 | K-40 | 0.025 | 0.007 | Gamma Spectrometry | [65] |
| Spain | Sheep milk | 6 | Cs-137 | 31.000 | 10.000 | Gamma Spectrometry | [65] |
| Spain | Goat milk | 2 | K-40 | 0.013 | 0.002 | Gamma Spectrometry | [65] |
| Spain | Goat milk | 2 | Cs-137 | 38.000 | 3.000 | Gamma Spectrometry | [65] |
| Spain | Cow milk | 1 | K-40 | 0.012 | 0.002 | Gamma Spectrometry | [65] |
| Spain | Cow milk | 1 | Pb-210 | 32.000 | 5.760 | Gamma Spectrometry | [65] |
| Spain | Yogurt | 1 | Cs-137 | 0.026 | 0.005 | Gamma Spectrometry | [65] |
| Spain | Yogurt | 1 | Cs-137 | 51.000 | 9.180 | Gamma Spectrometry | [65] |
| Slovenia | Powder milk | 6 | Cs-137 | 0.039 | 0.013 | Gamma Spectrometry | [66] |

**Supplementary appendix 2**. Consumption rate of milk based on country [67]

| **Country** | **IR (l/year)** |
| --- | --- |
| Austria | 70.1 |
| France | 55.7 |
| USA | 65.1 |
| Czech Republic | 60 |
| Kazakhstan | 60.2 |
| Italy | 57.3 |
| Singapore | 18.2 |
| Vietnam | 12.1 |
| Thailand | 14.3 |
| India | 66.3 |
| Egypt | 28.5 |
| Mali | 8.7 |
| Tunisia | 51.9 |
| Russia | 59.4 |
| UK | 68.5 |
| Bosnia and Herzegovina | 62.8 |
| South Korea | 32.7 |
| Japan | 31.4 |
| Romania | 75.6 |
| Spain | 56.3 |
| Switzerland | 64.5 |
| Iceland | 73.2 |
| Malaysia | 20.8 |
| Saudi Arabia | 45.6 |
| Serbia | 61.2 |
| New Zealand | 82.3 |
| Jordan | 38.9 |
| Lebanon | 42.5 |
| Greece | 58.7 |
| Bangladesh | 9.50 |
| Algeria | 47.2 |
| Syria | 33.8 |
| Germany | 63.8 |

**Supplementary appendix 3.** Radionuclide-specific cancer risk factors per Bq ingested [68-70]

| Radionuclide | Radionuclide-specific cancer risk factors |
| --- | --- |
| Cs-137 | 1.30 × 10⁻⁸ |
| Cs-134 | 1.90 × 10⁻⁸ |
| I-131 | 2.20 × 10⁻⁸ |
| Pb-210 | 6.90 × 10⁻⁷ |
| K-40 | 6.68 × 10⁻^10^ |

**Supplementary appendix 4.** The MCS model for determine CR of Cs-137 in milk


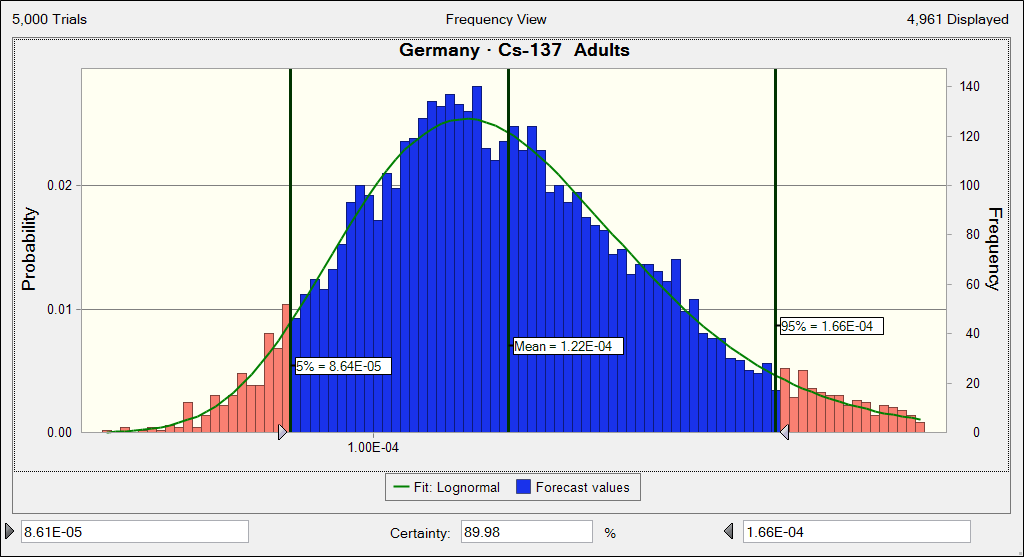


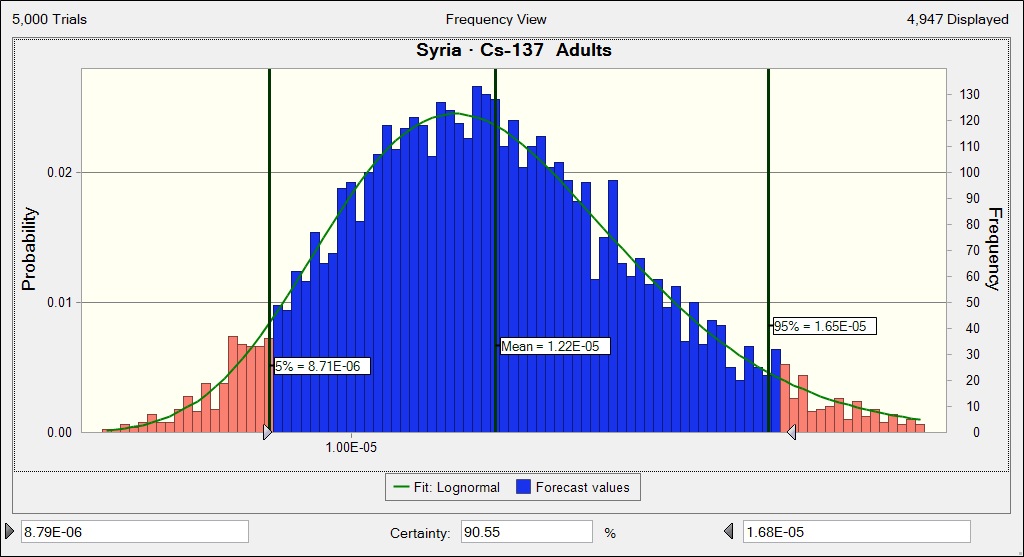


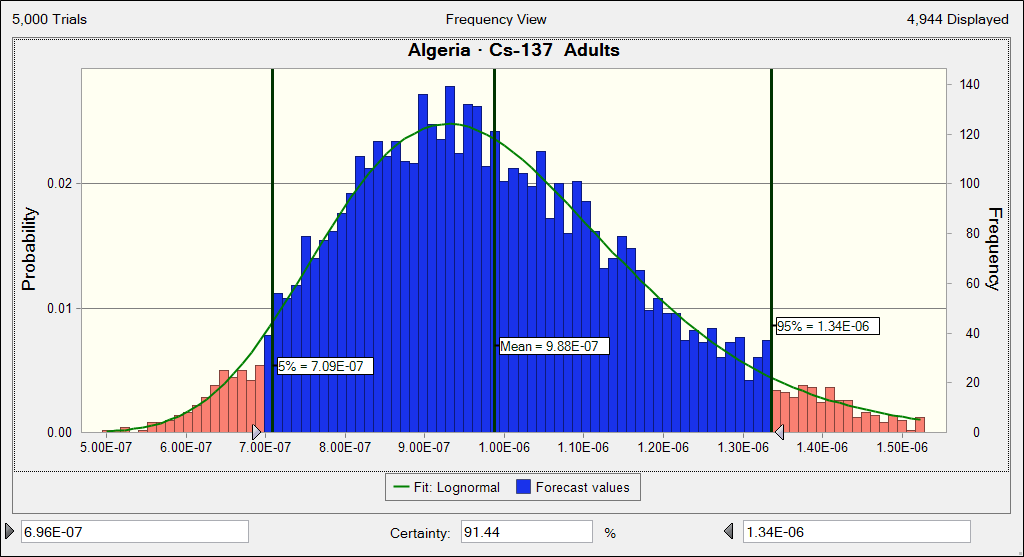


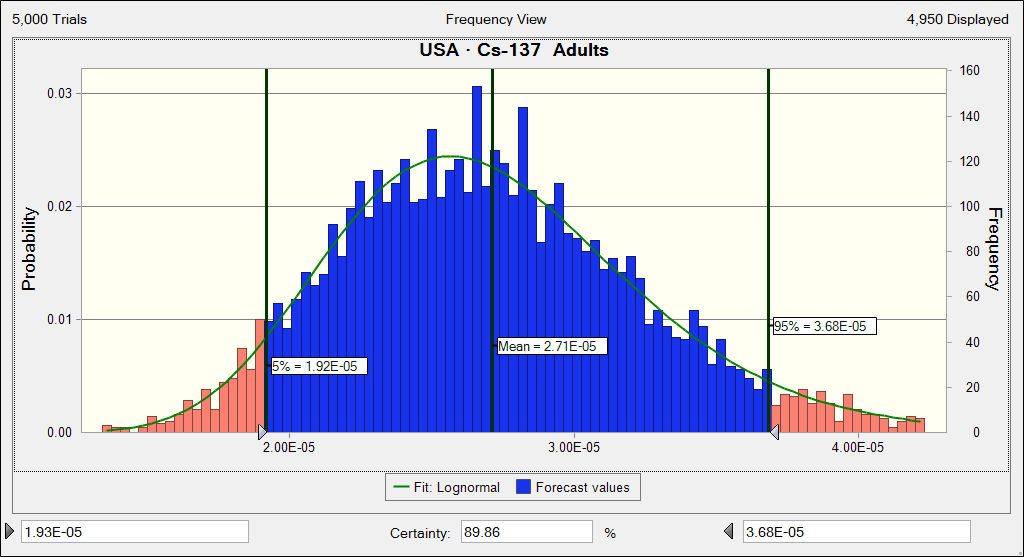


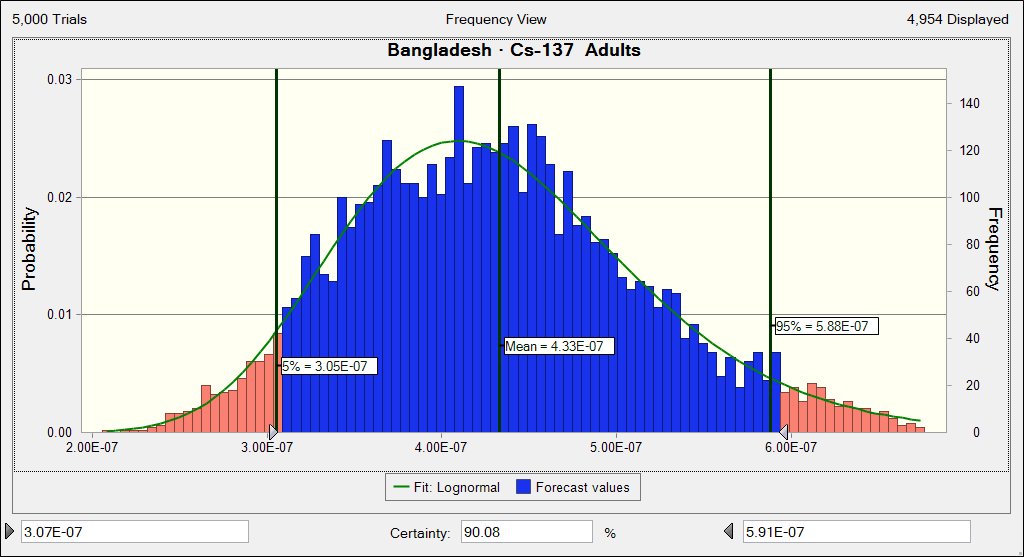


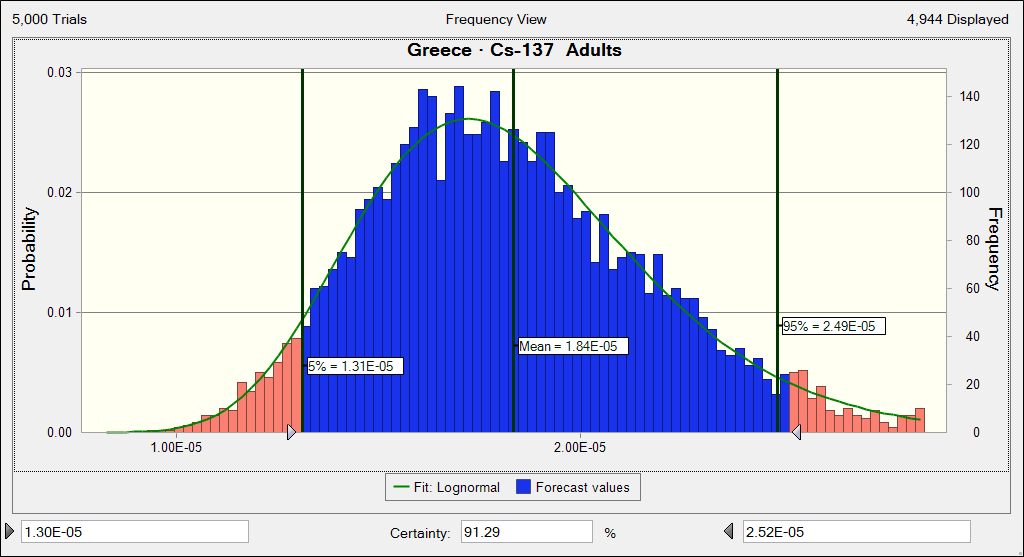


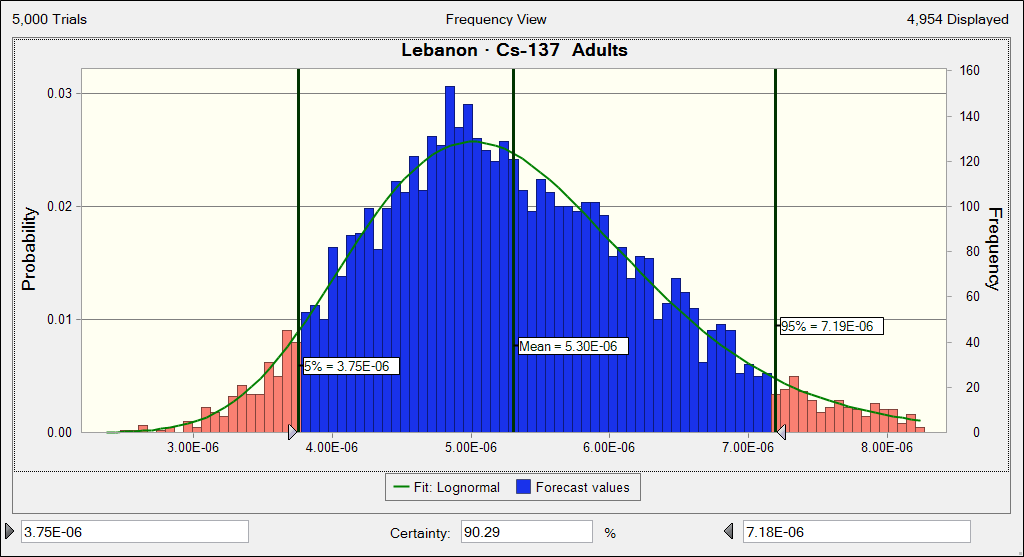


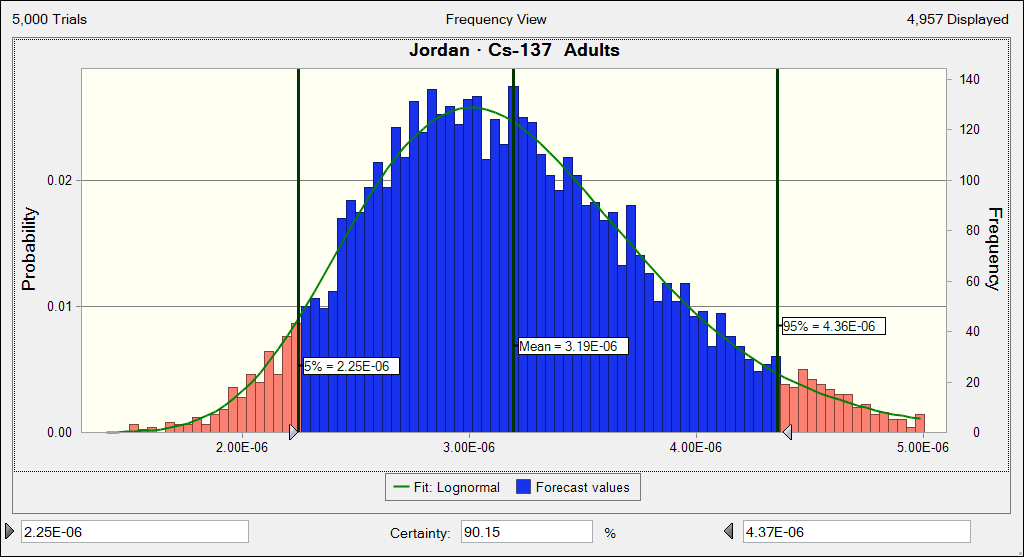


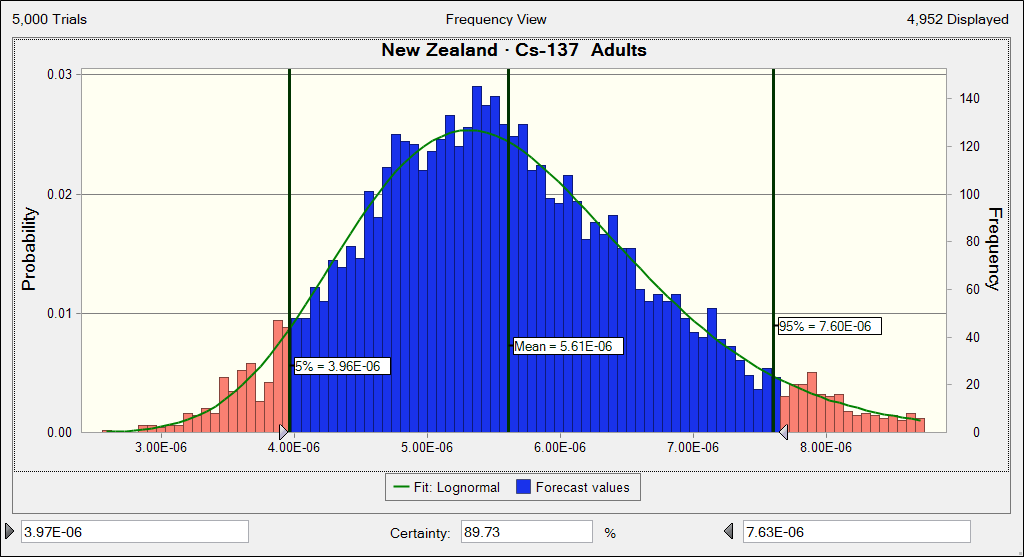


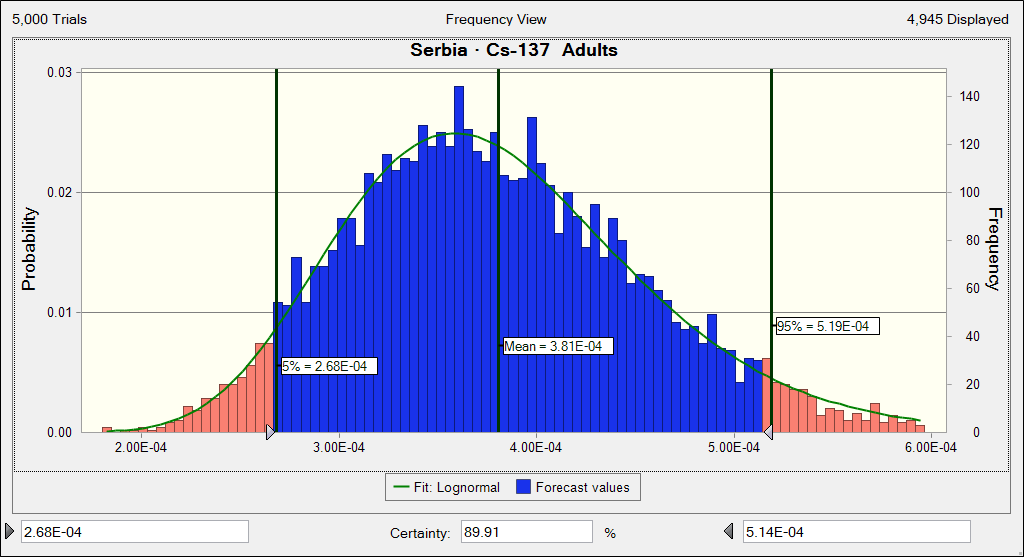


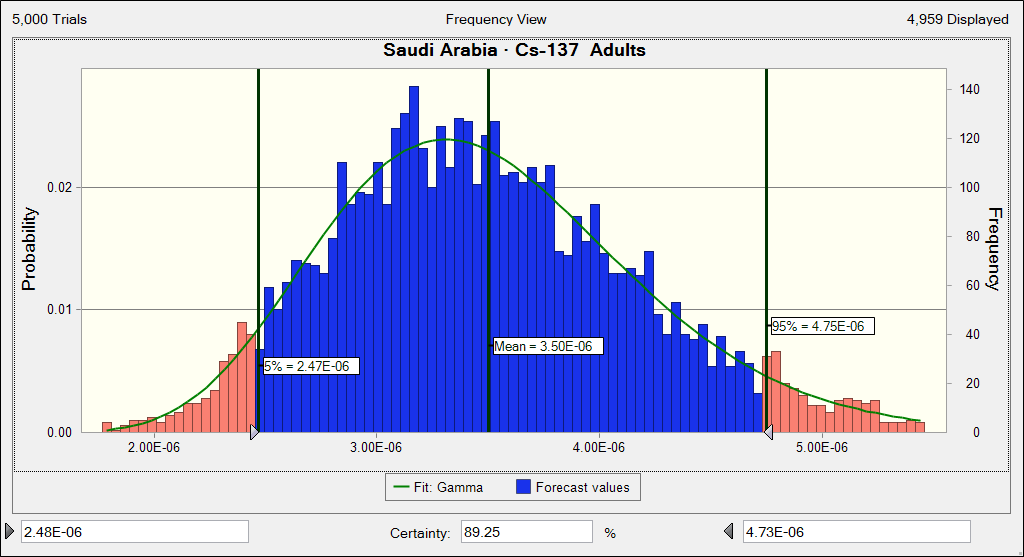


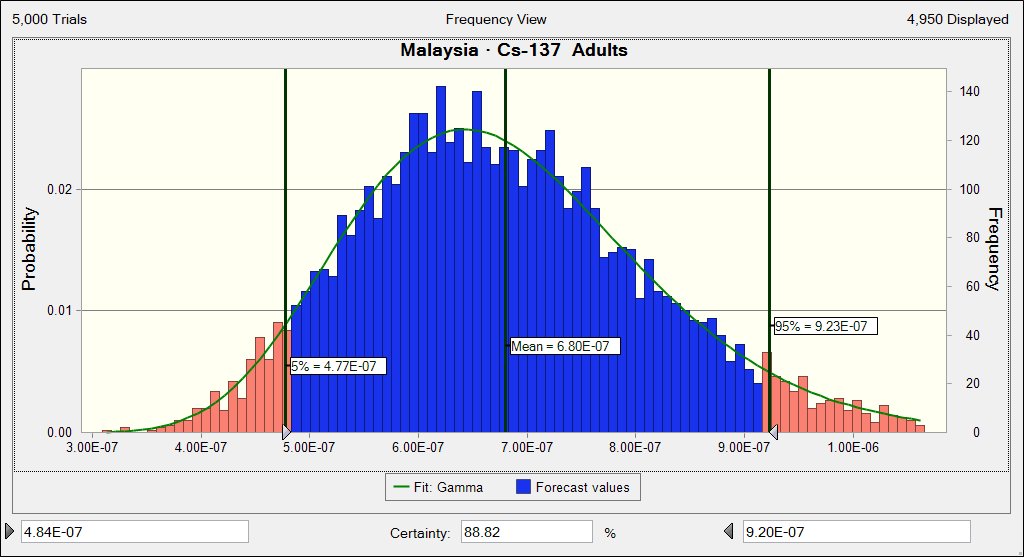


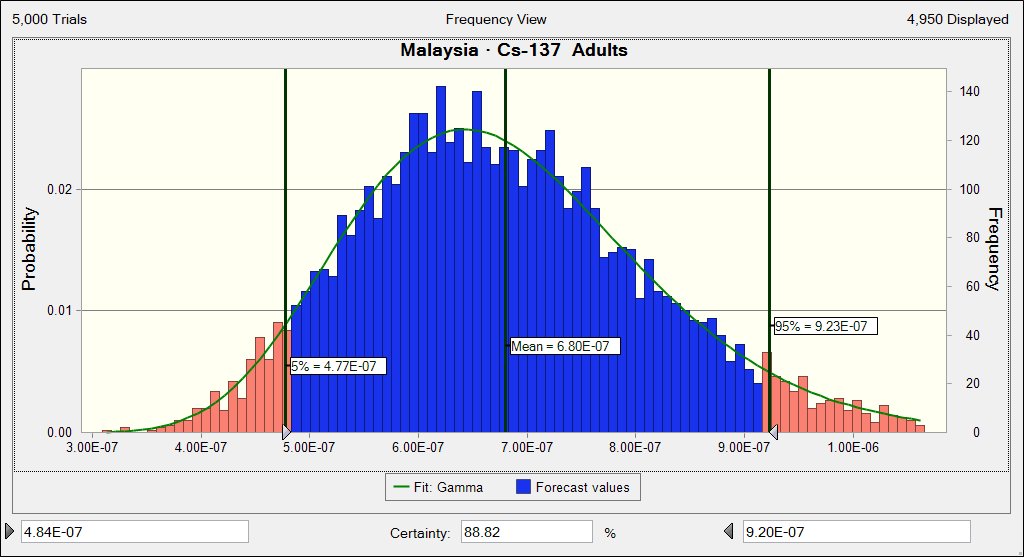


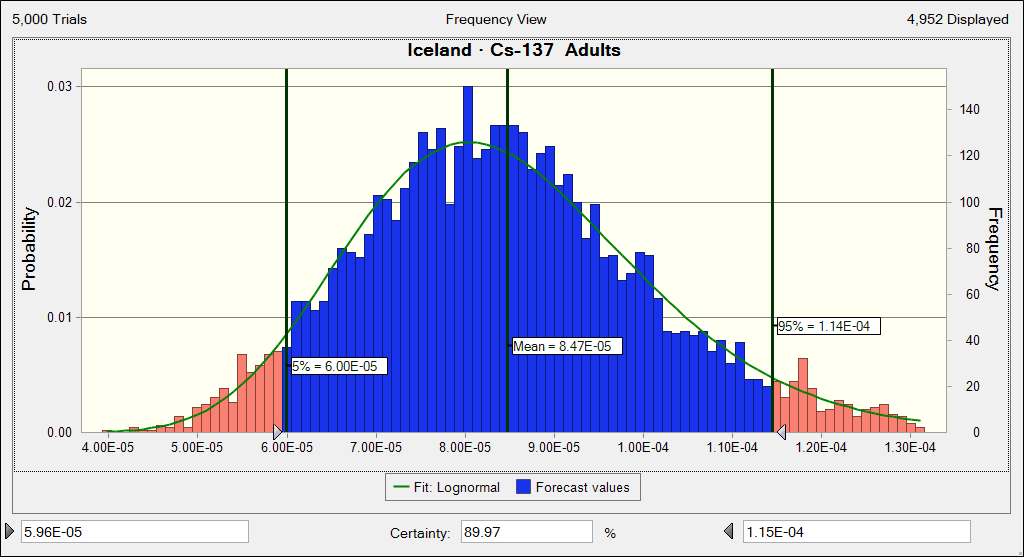


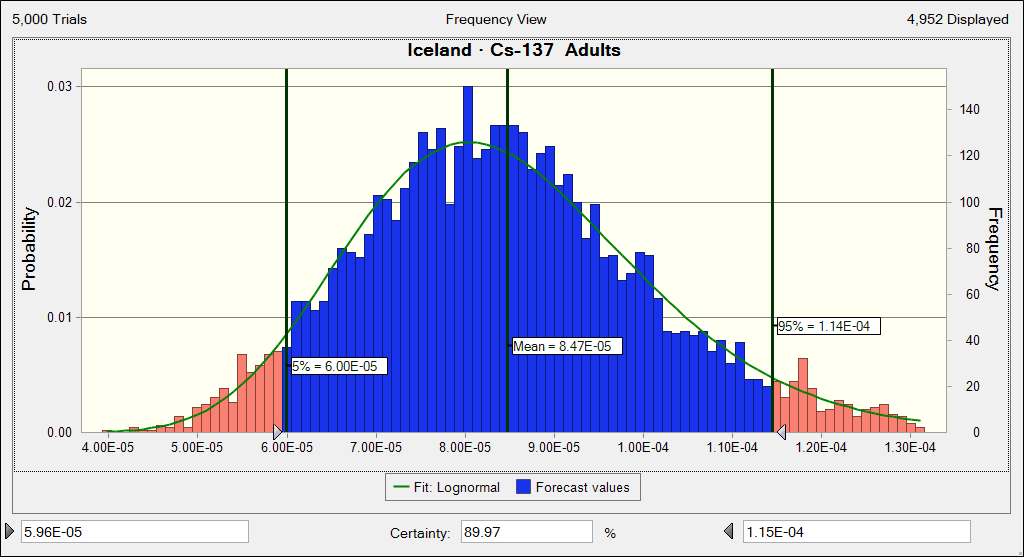


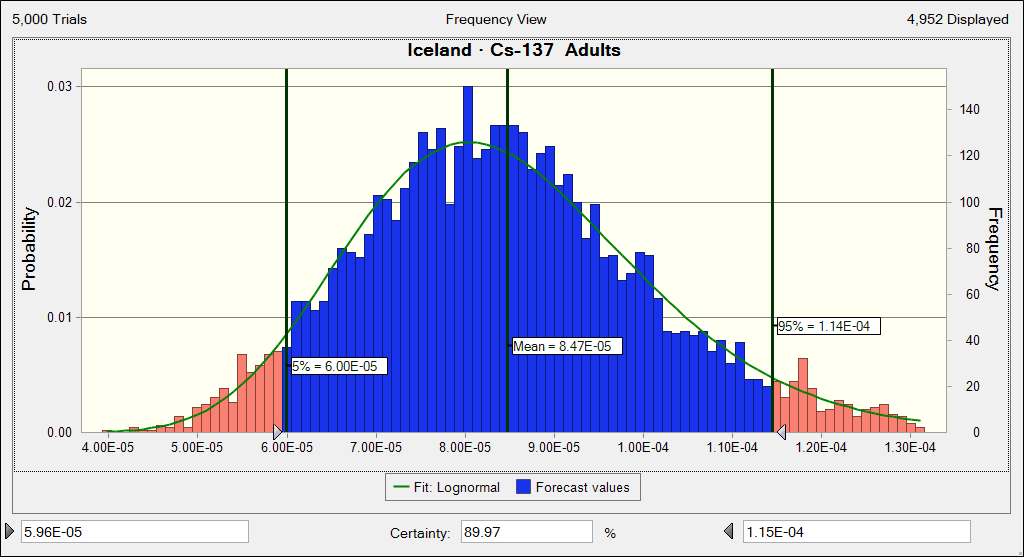


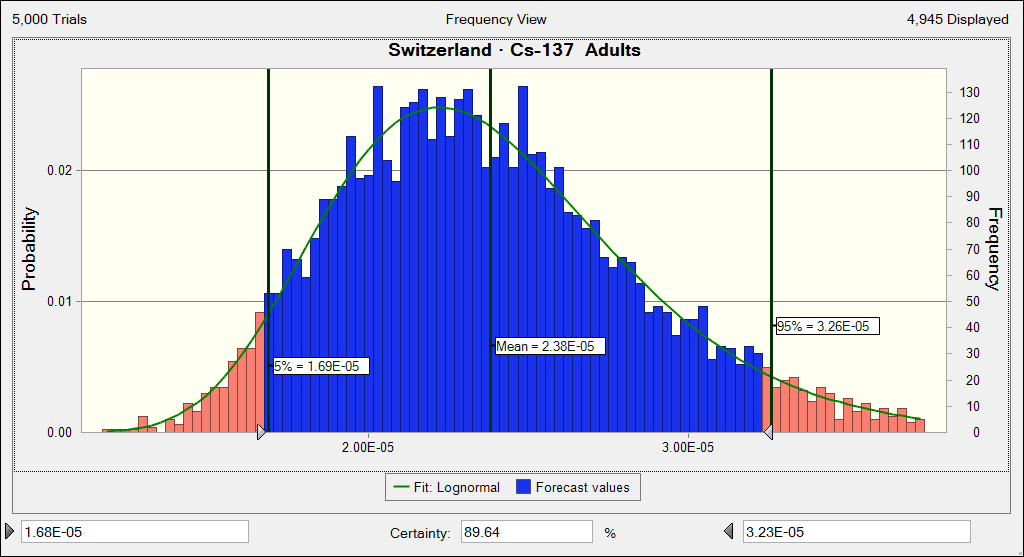


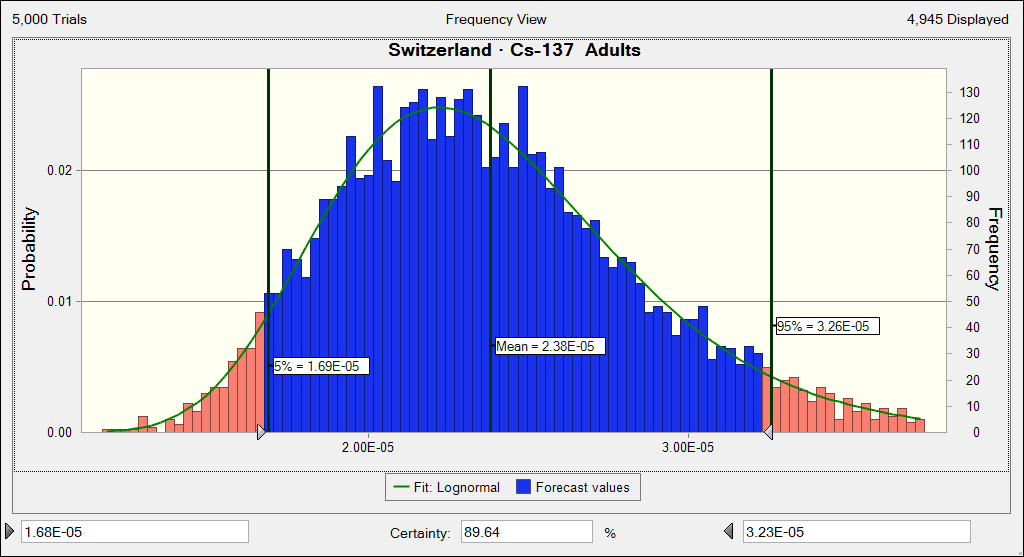


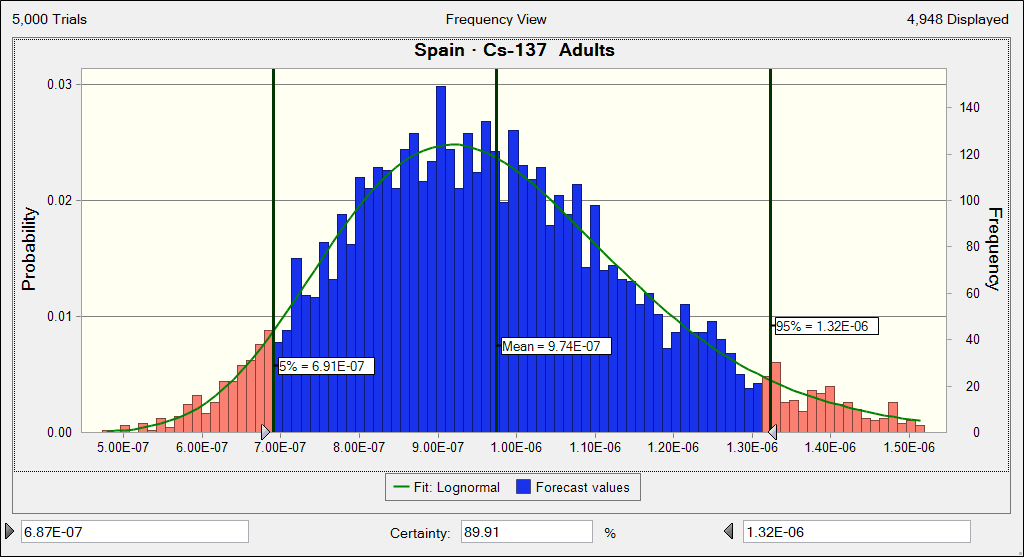


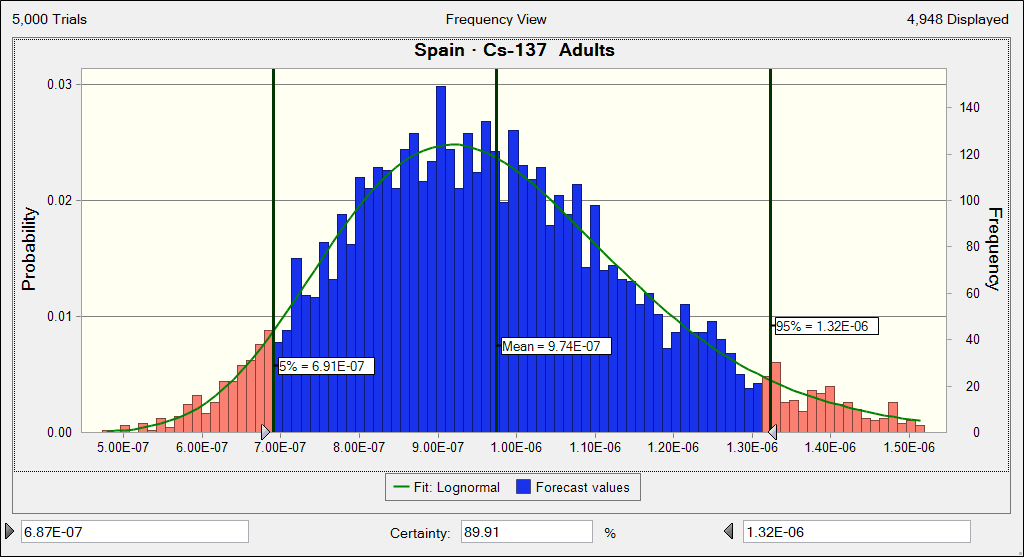


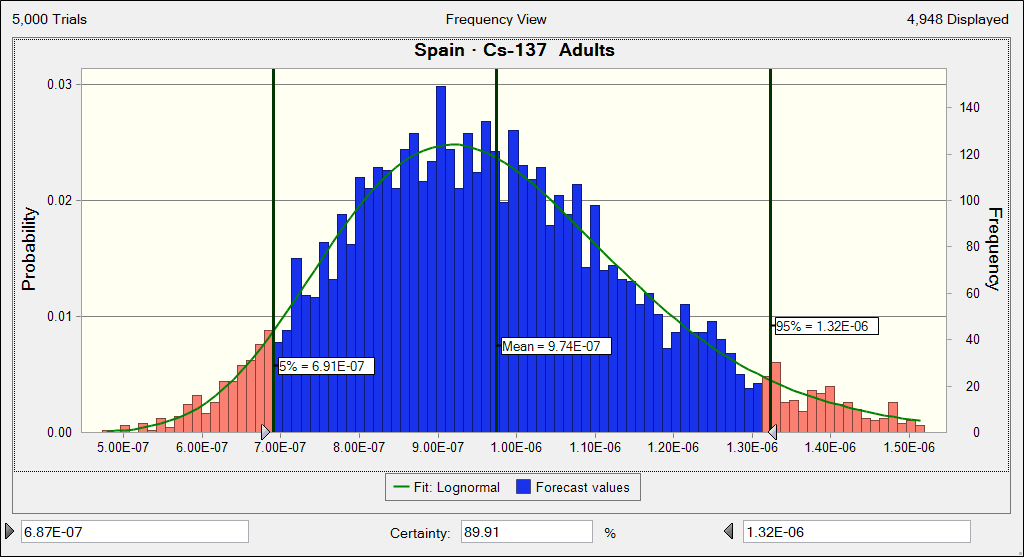


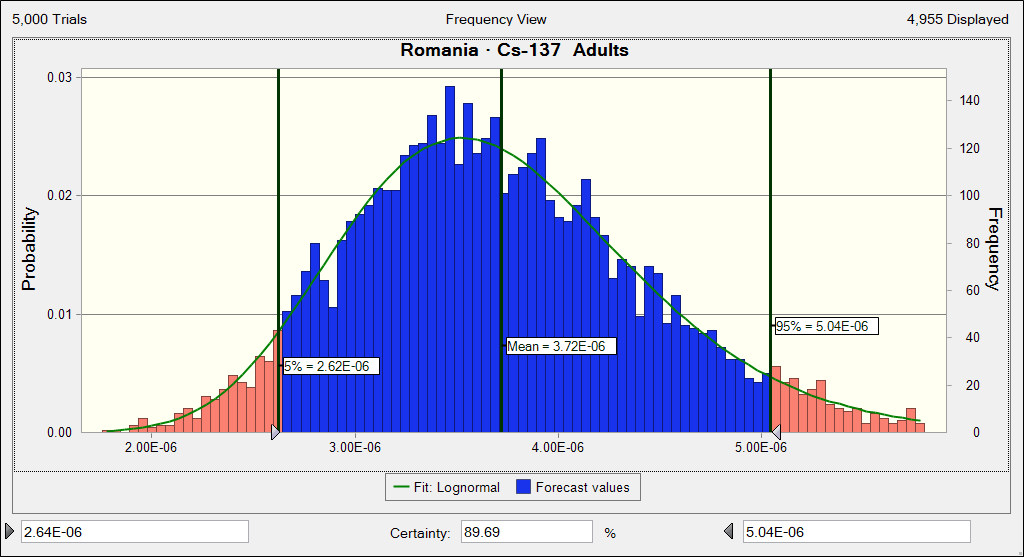


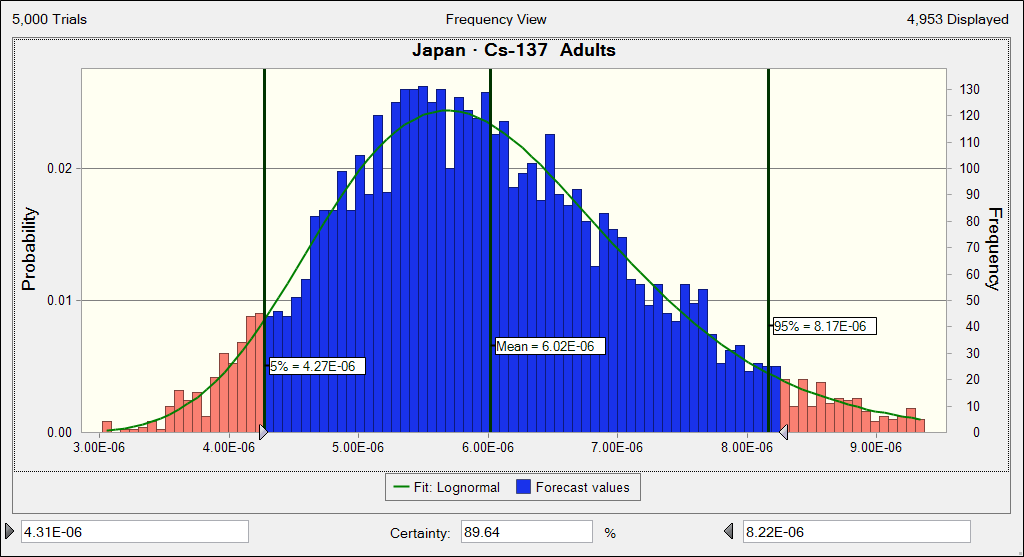


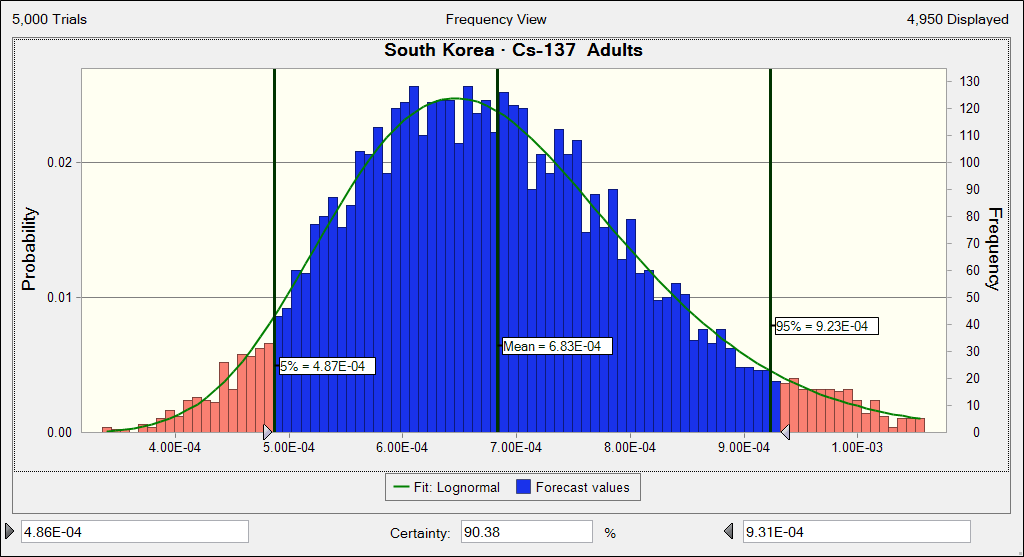


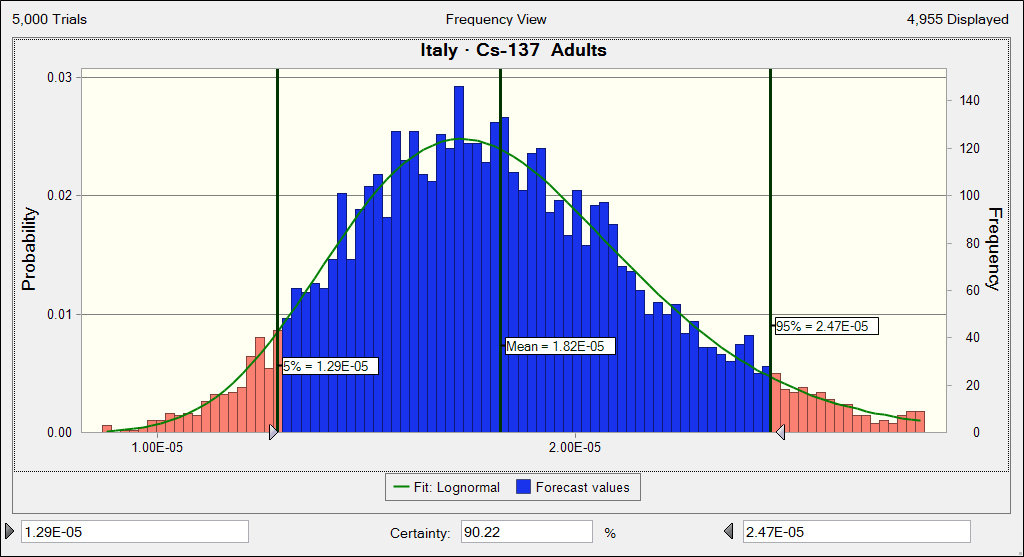


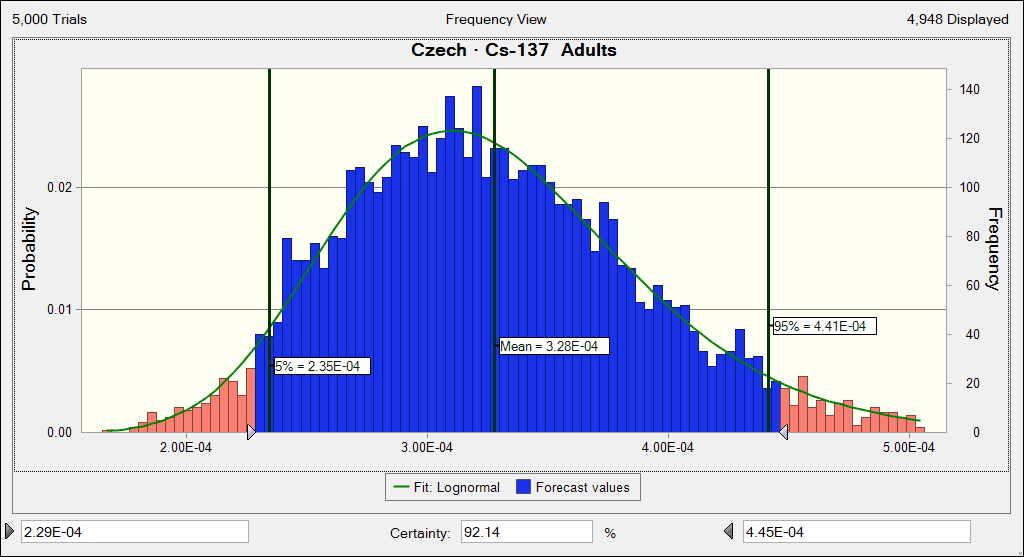


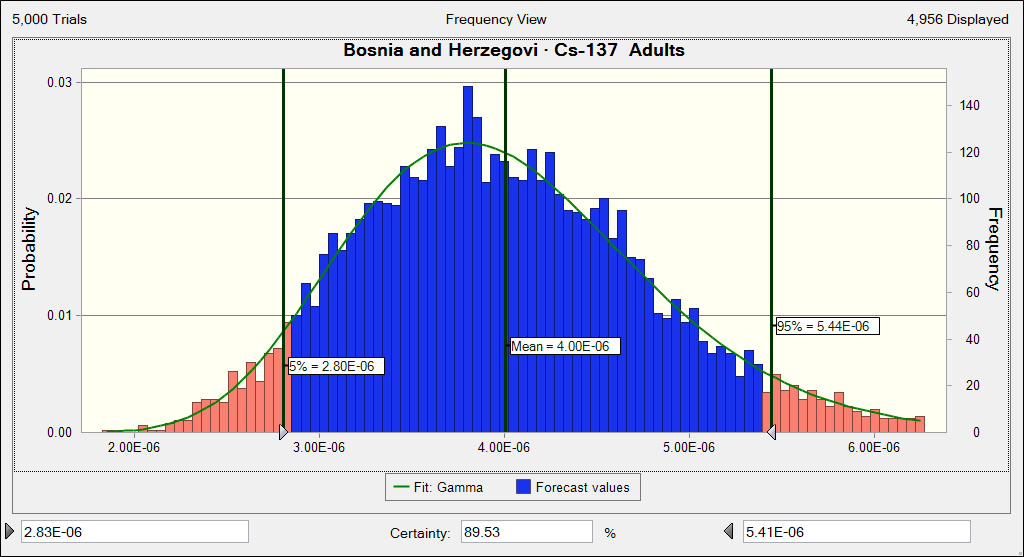


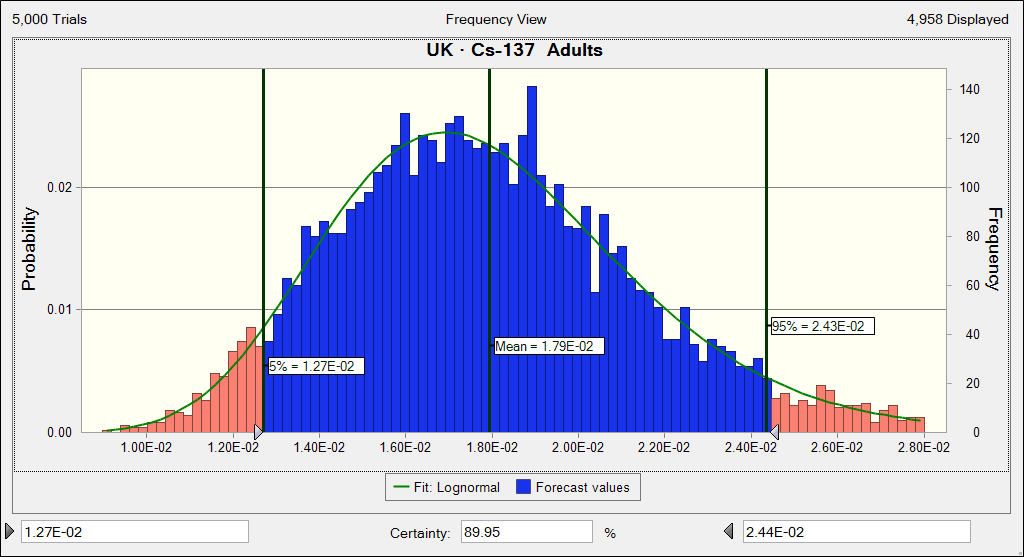


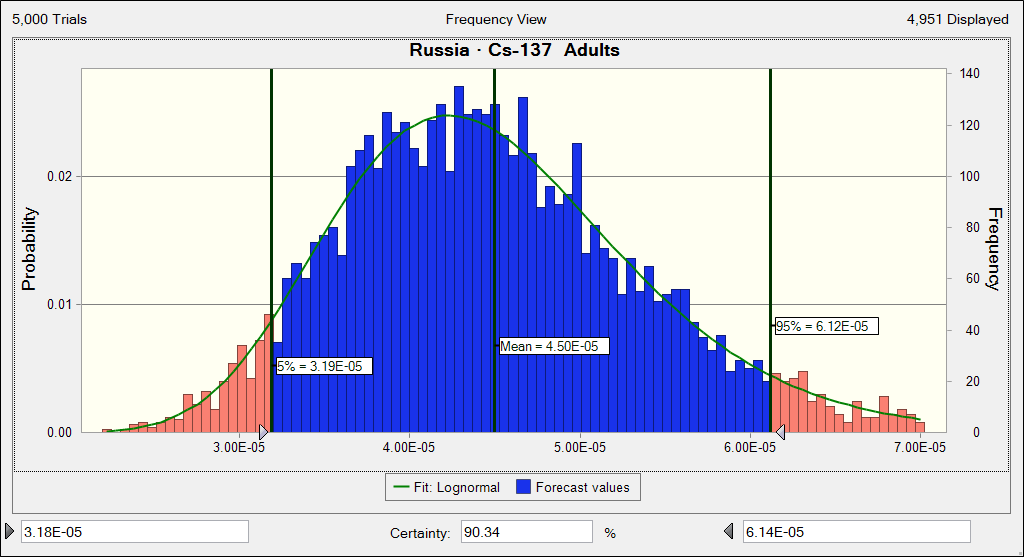


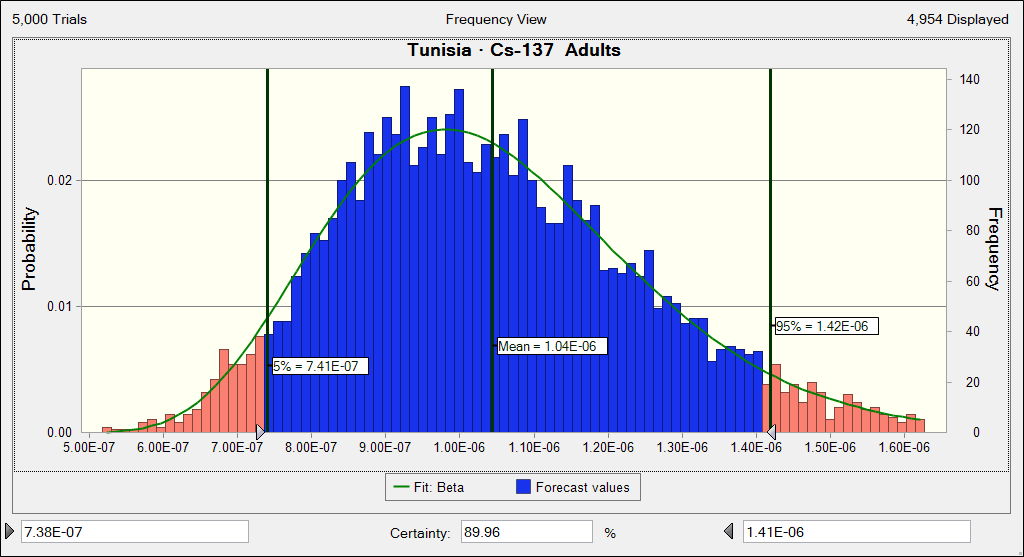


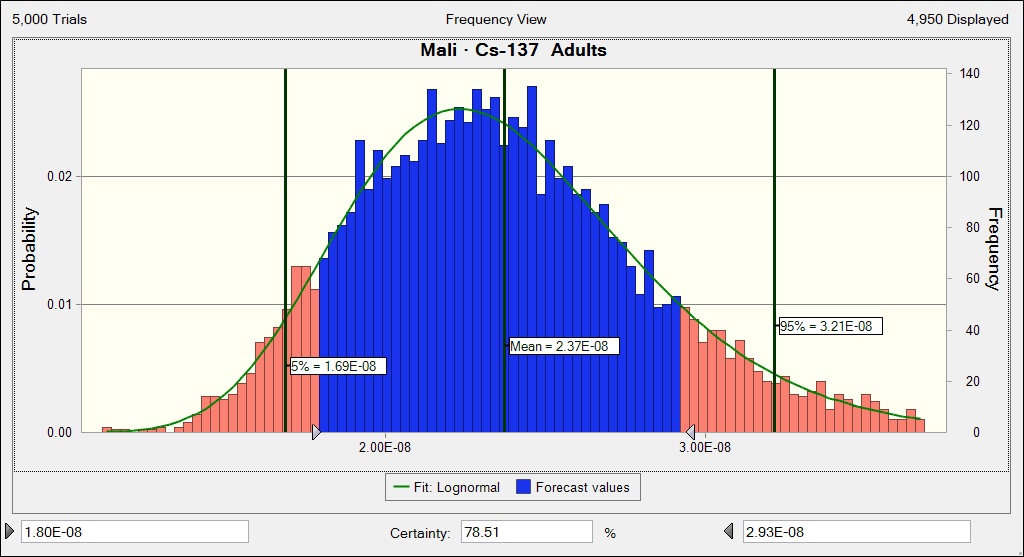


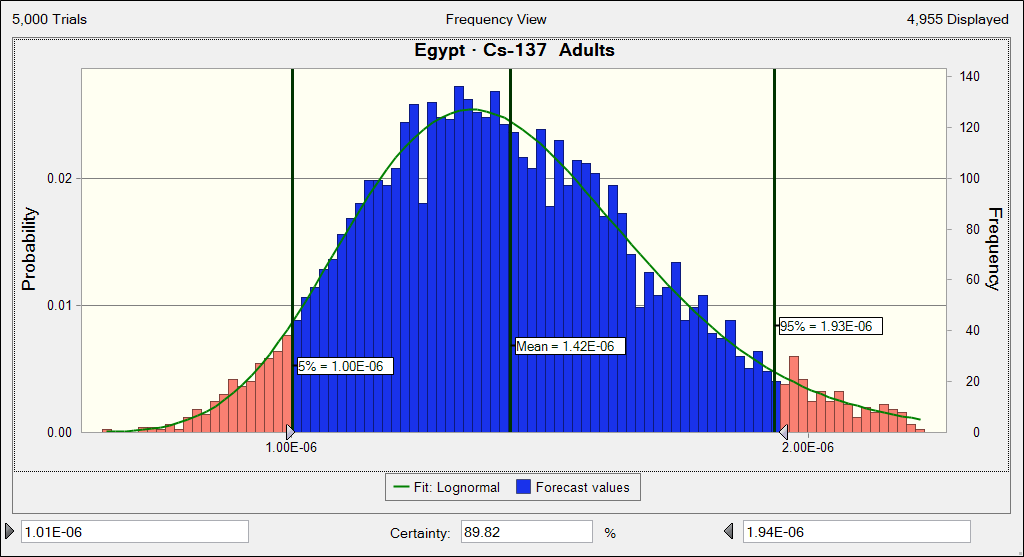


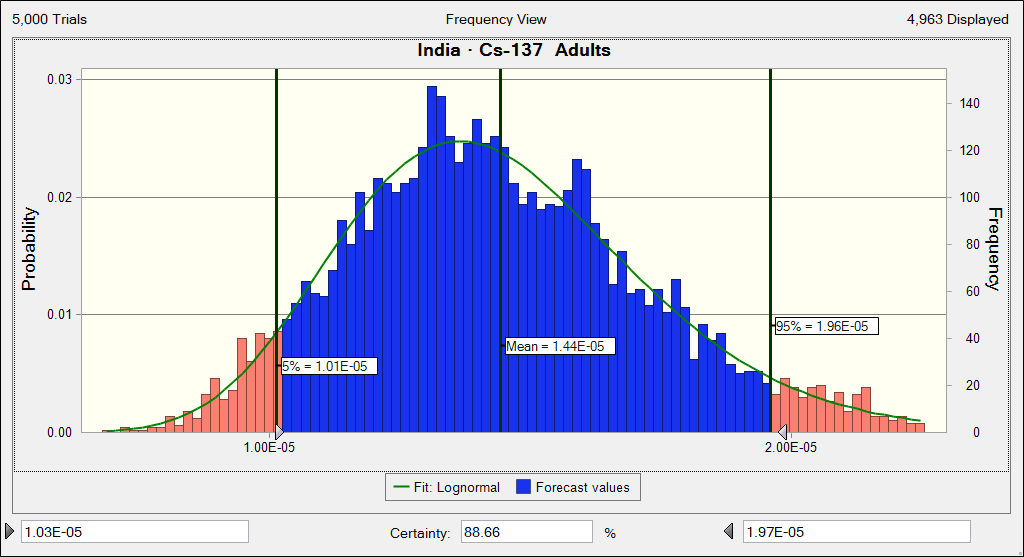


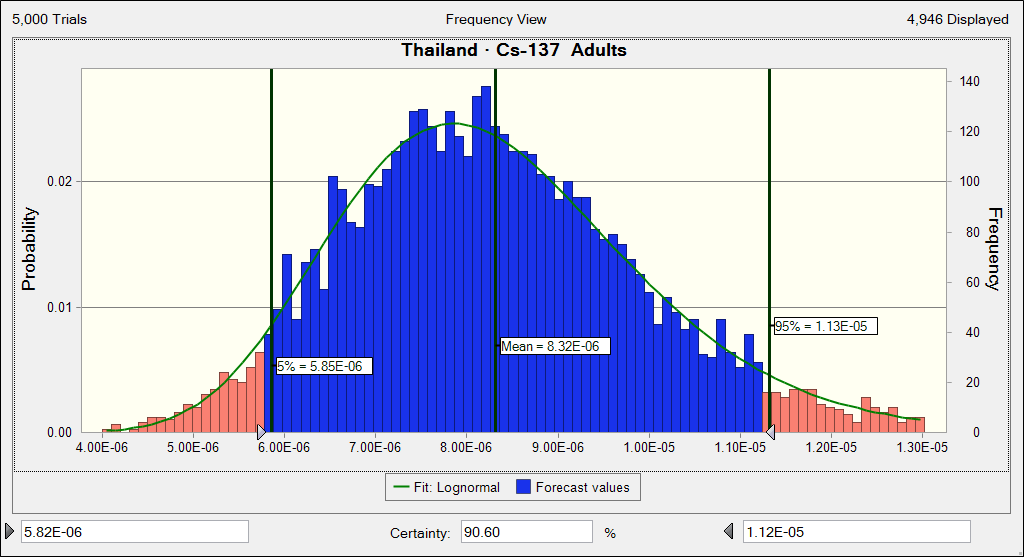


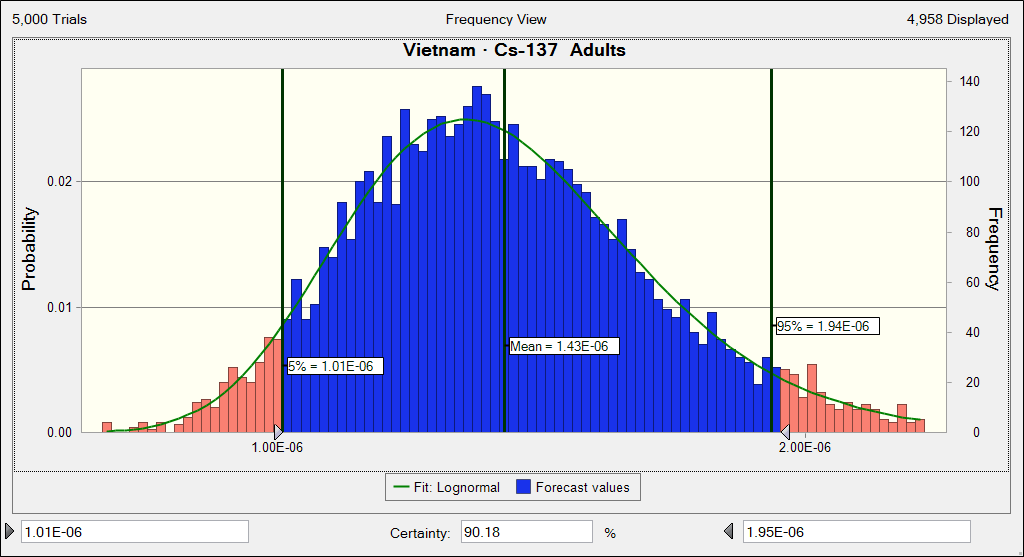


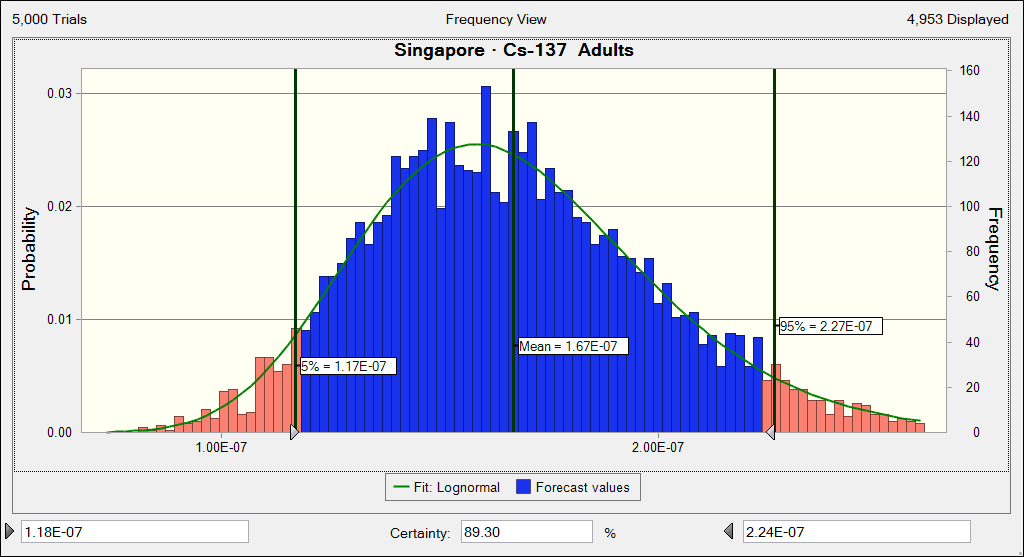


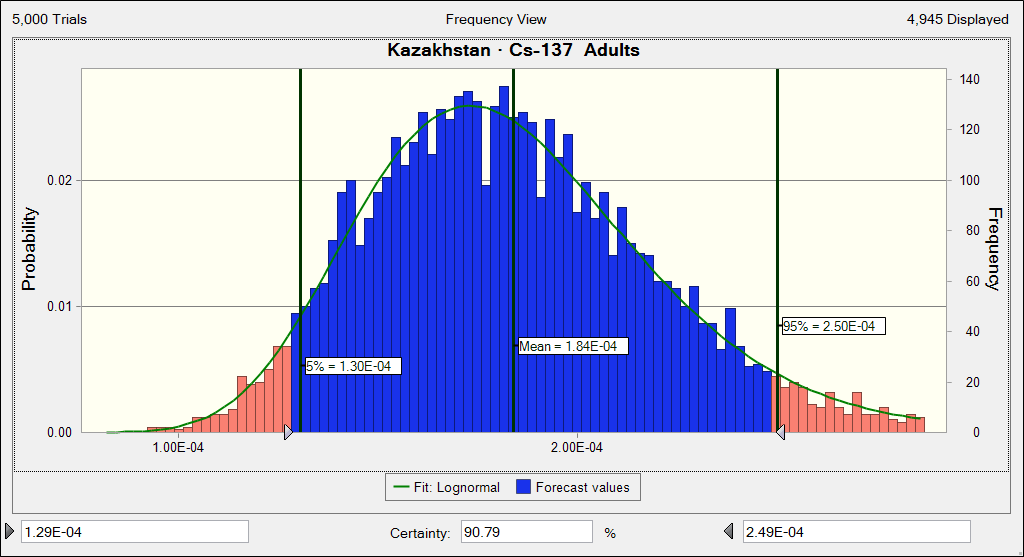


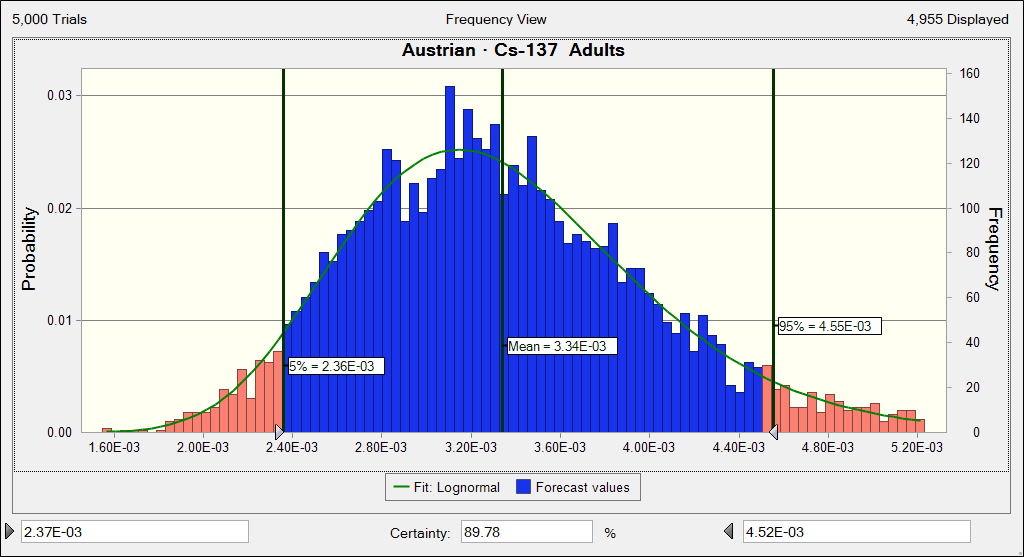


**Supplementary appendix 5.** The MCS model for determine CR of Cs-134 in milk
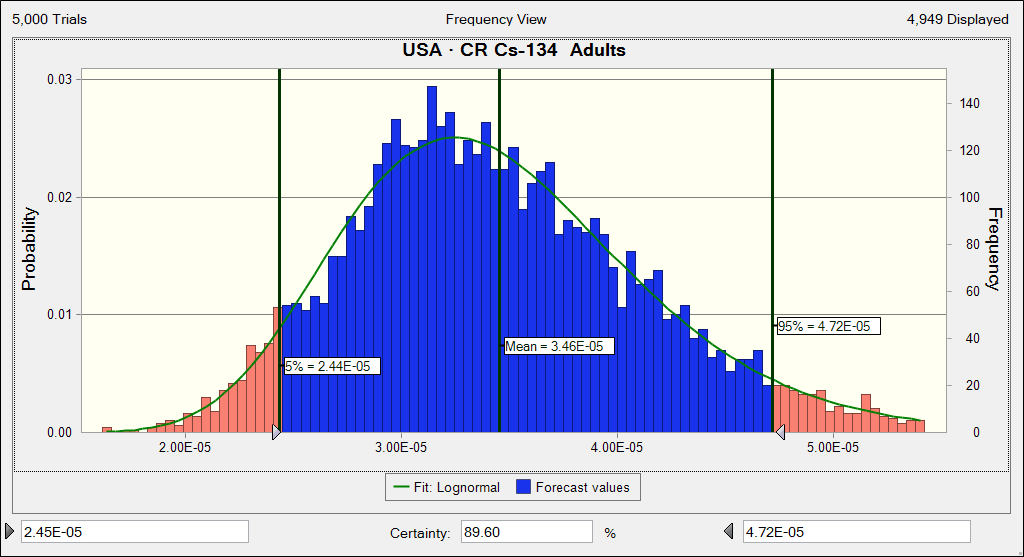


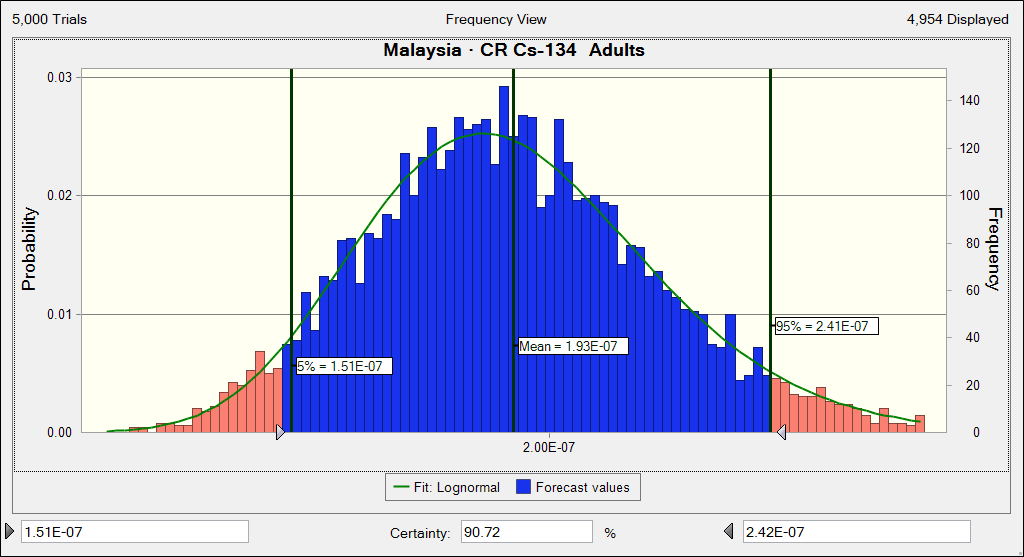


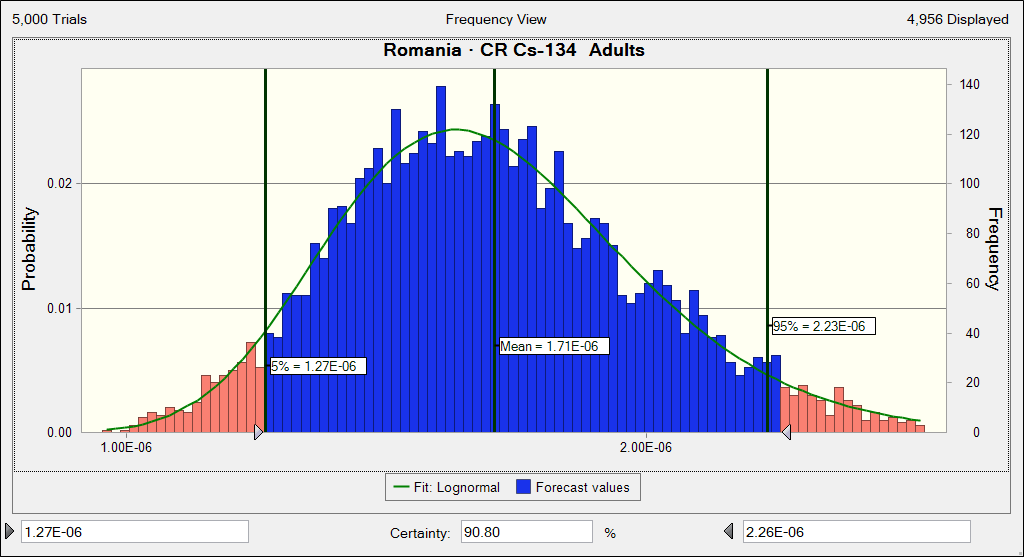


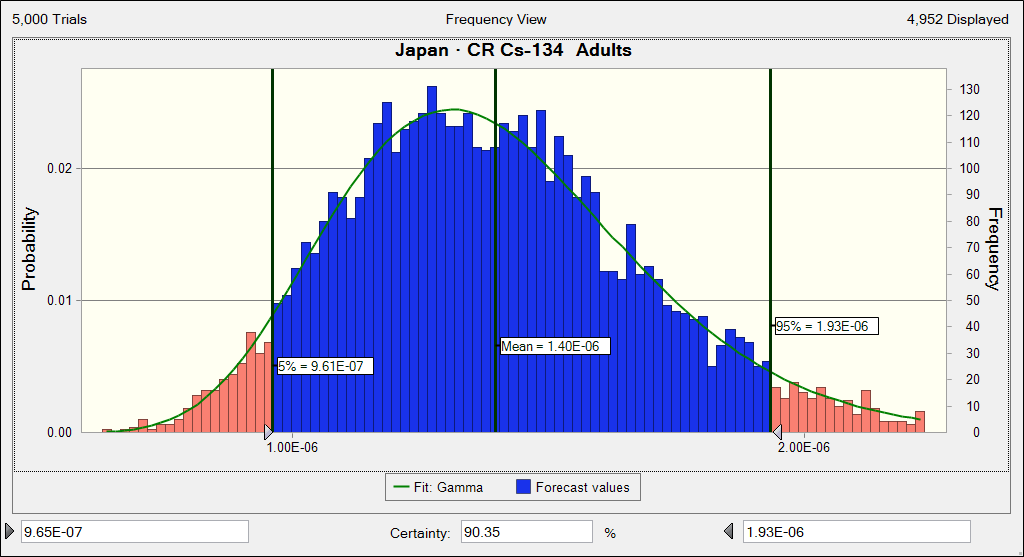


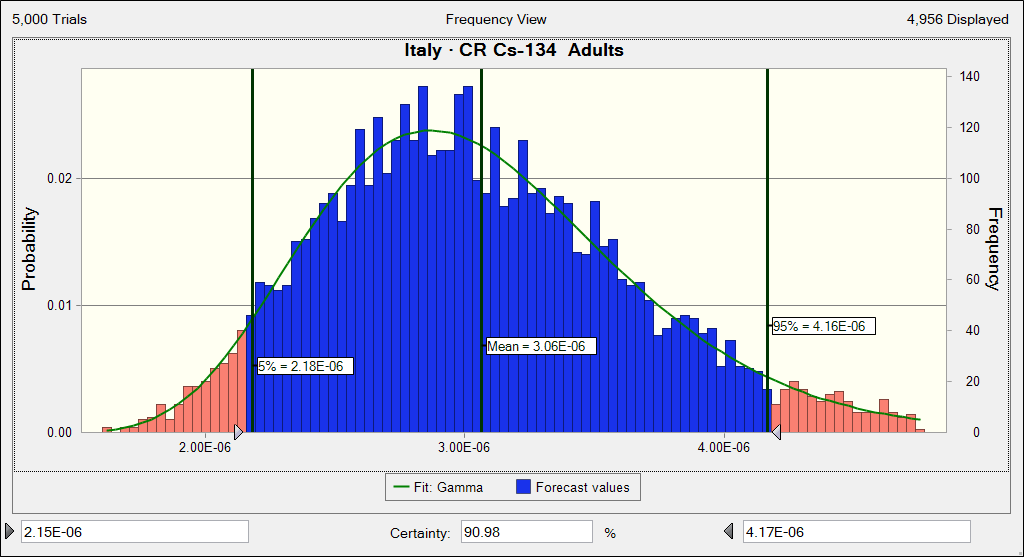


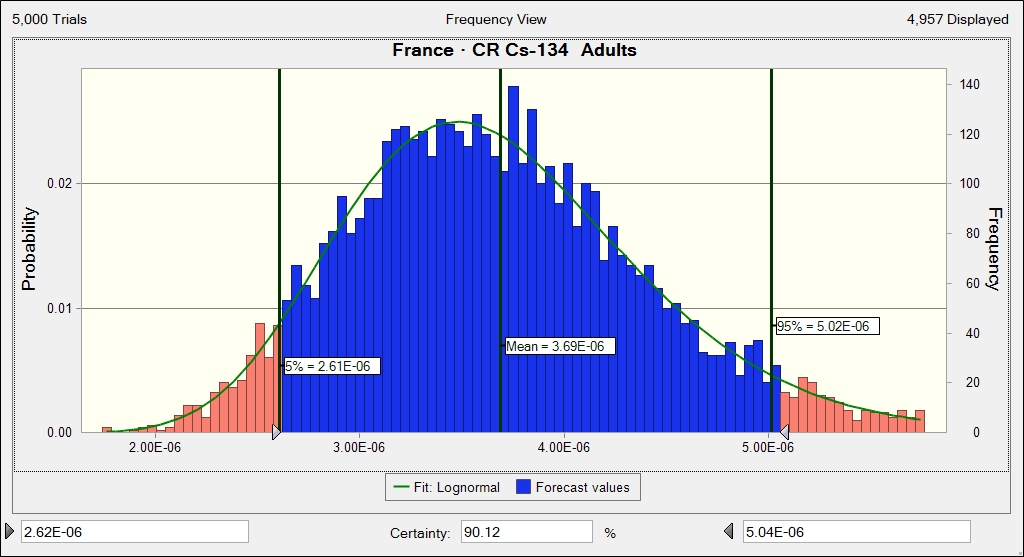


**Supplementary** appendix 6. The MCS model for determine CR of K-40 in milk


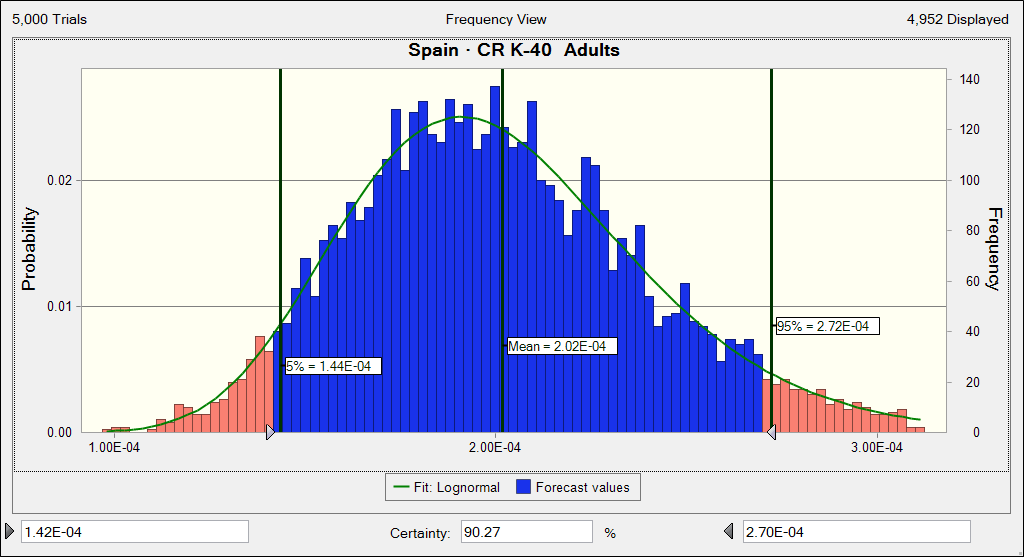


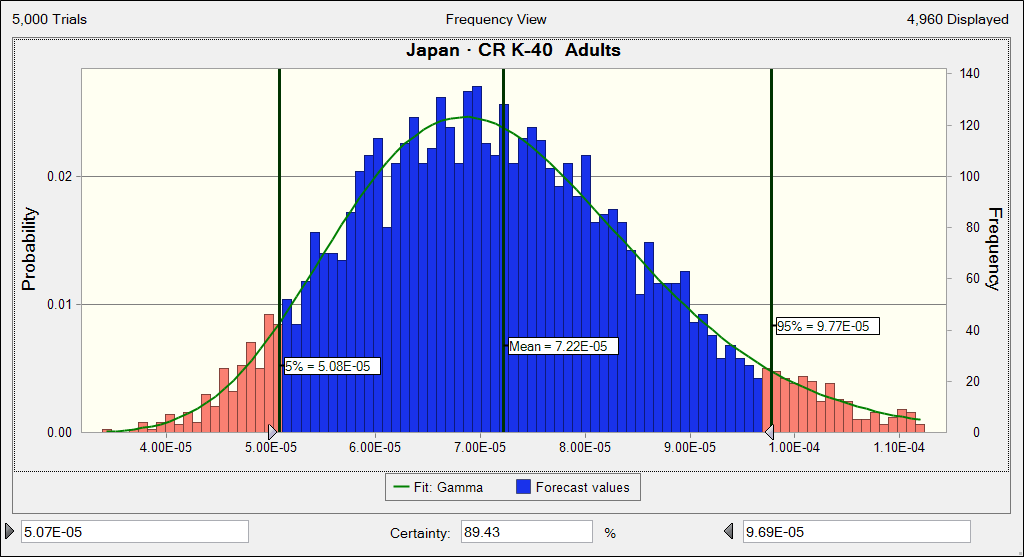


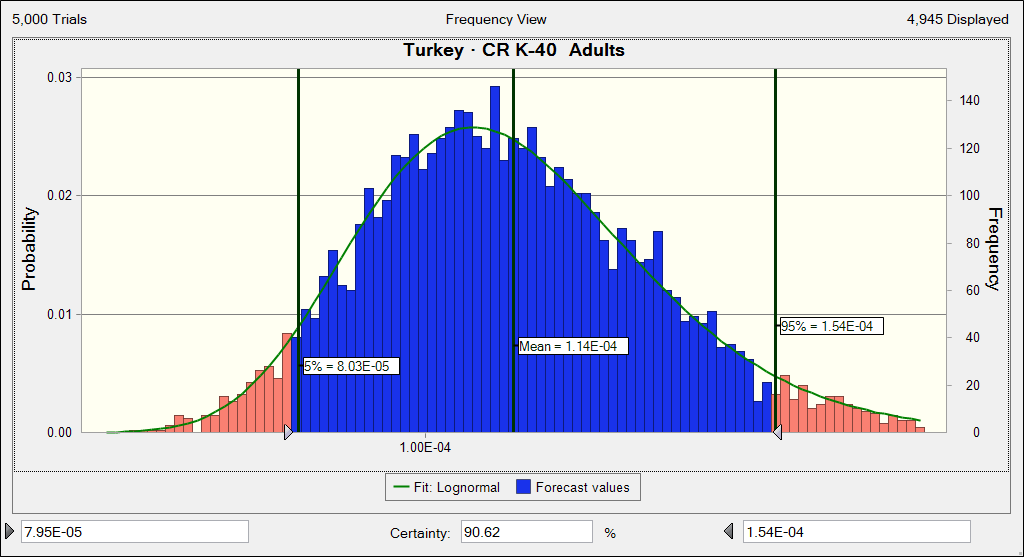


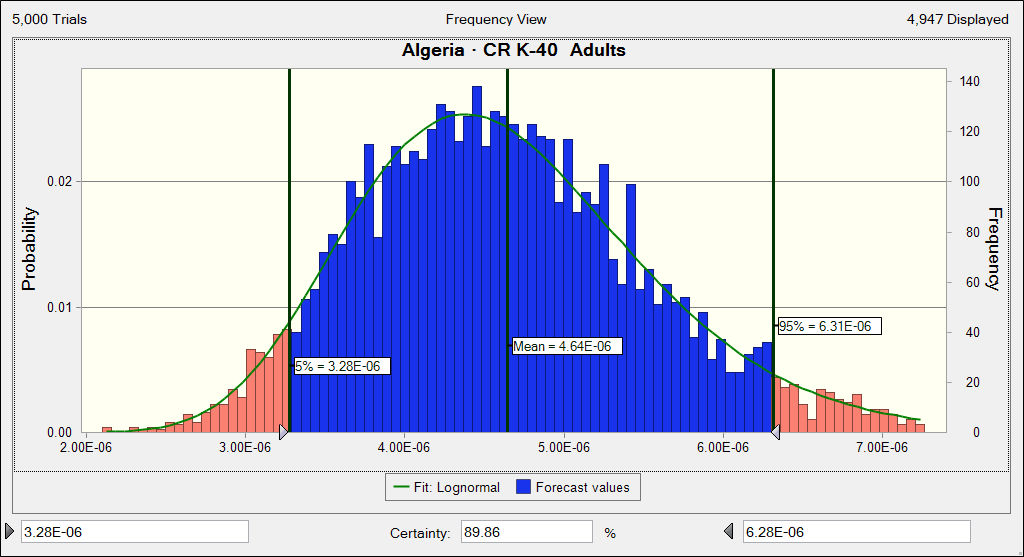


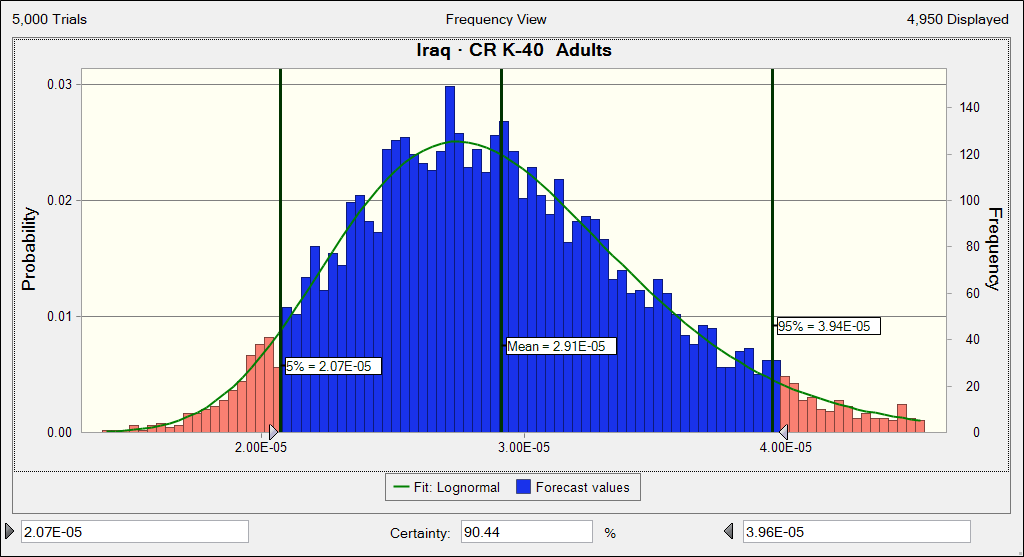


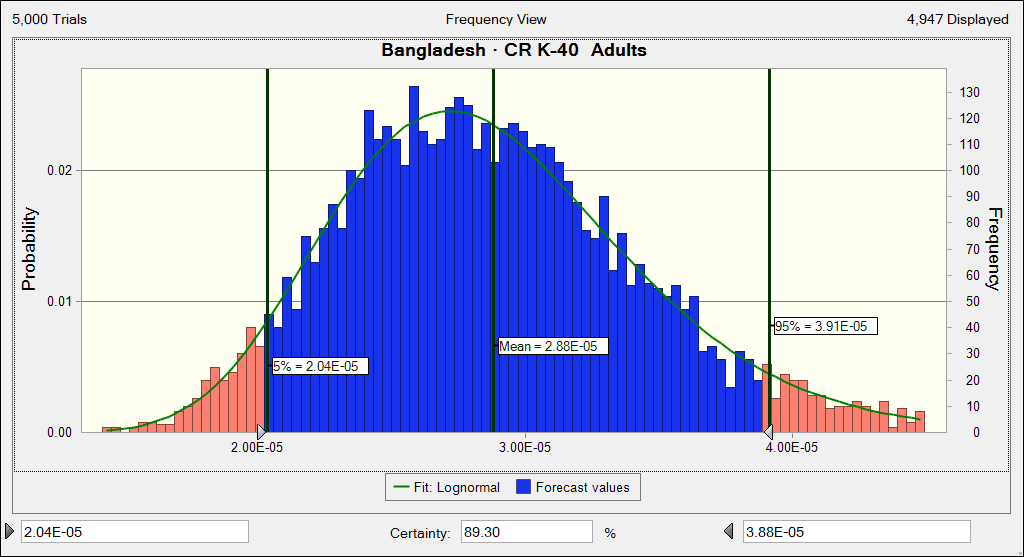


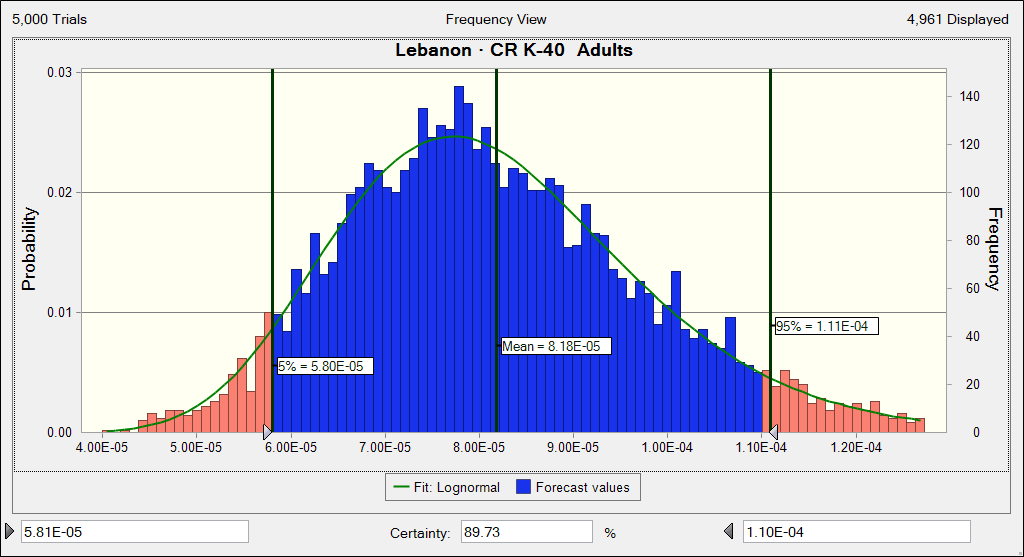


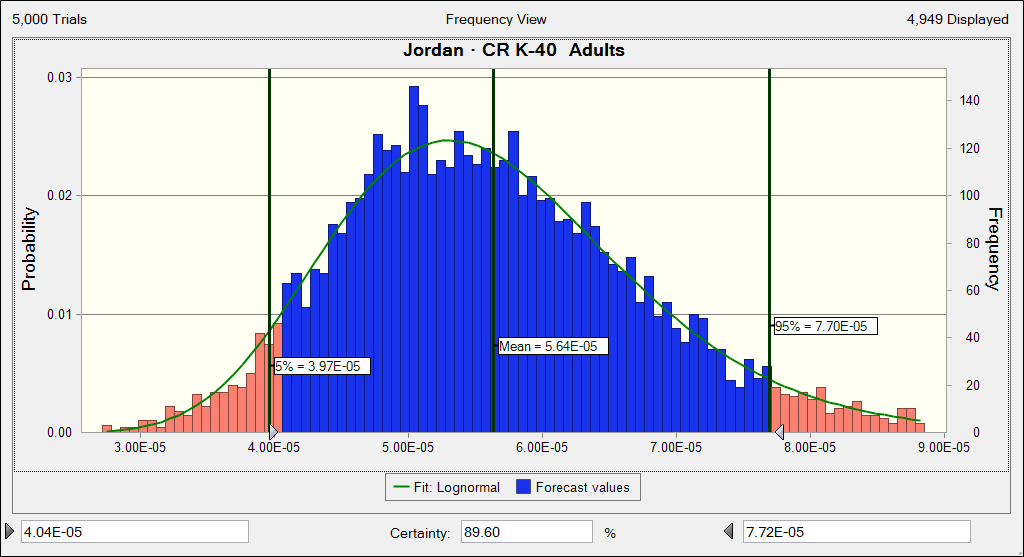


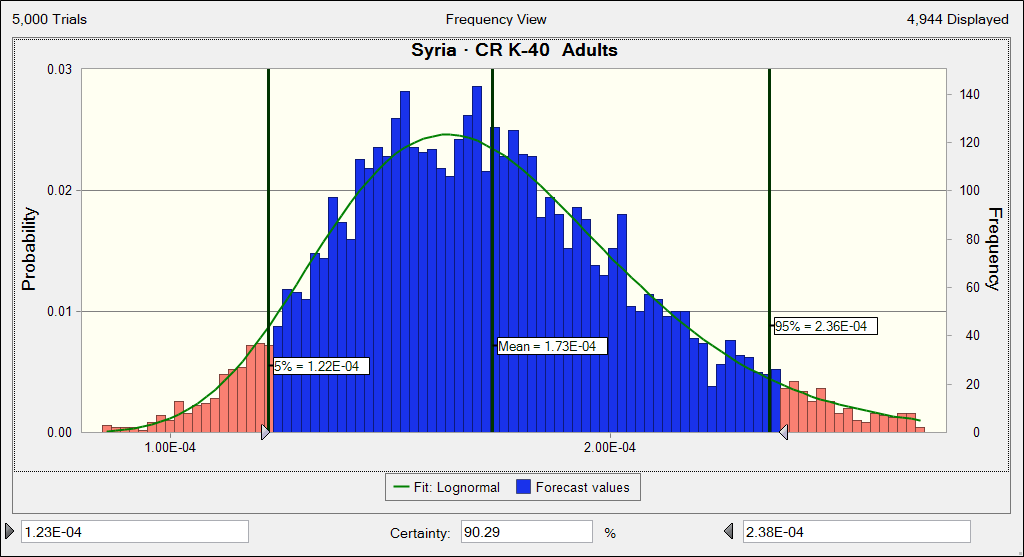


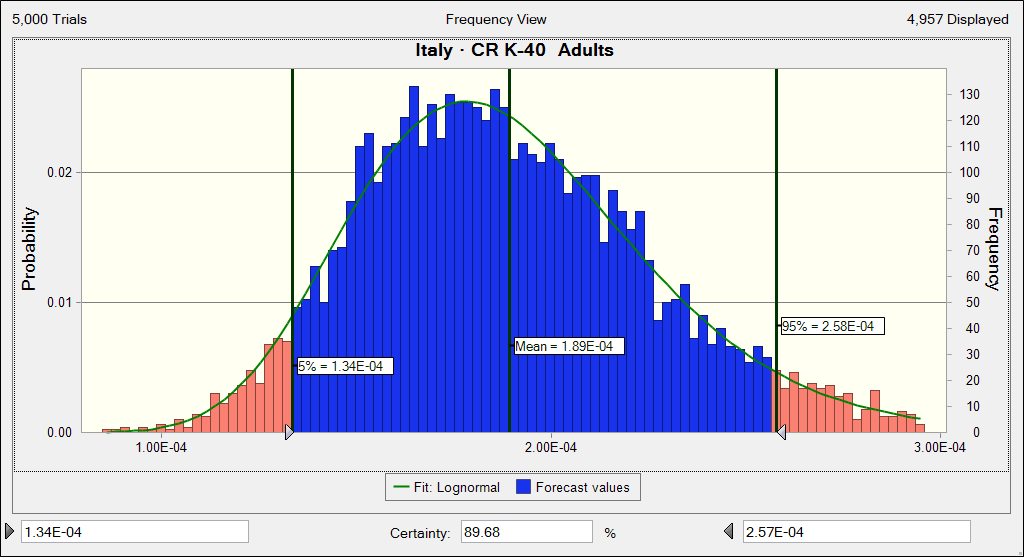


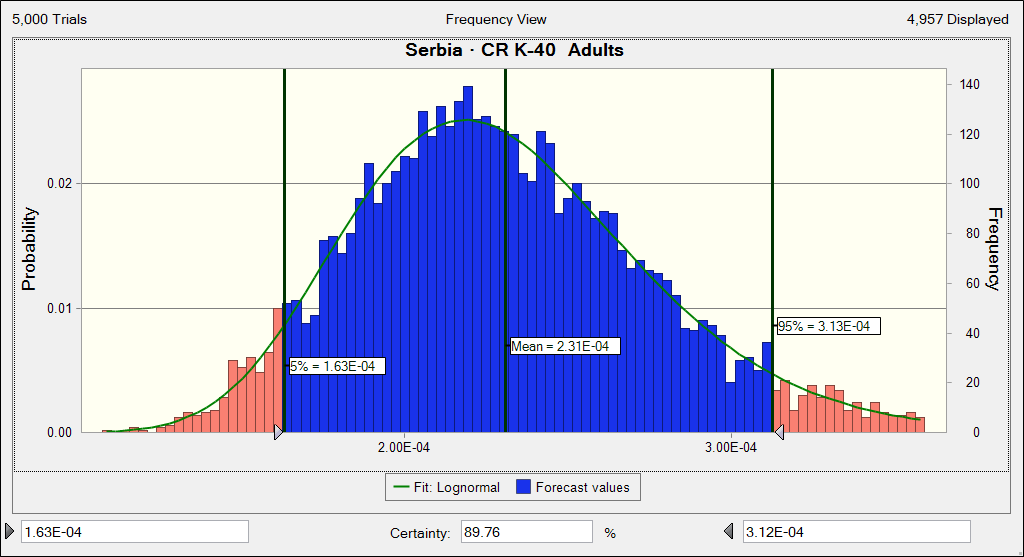


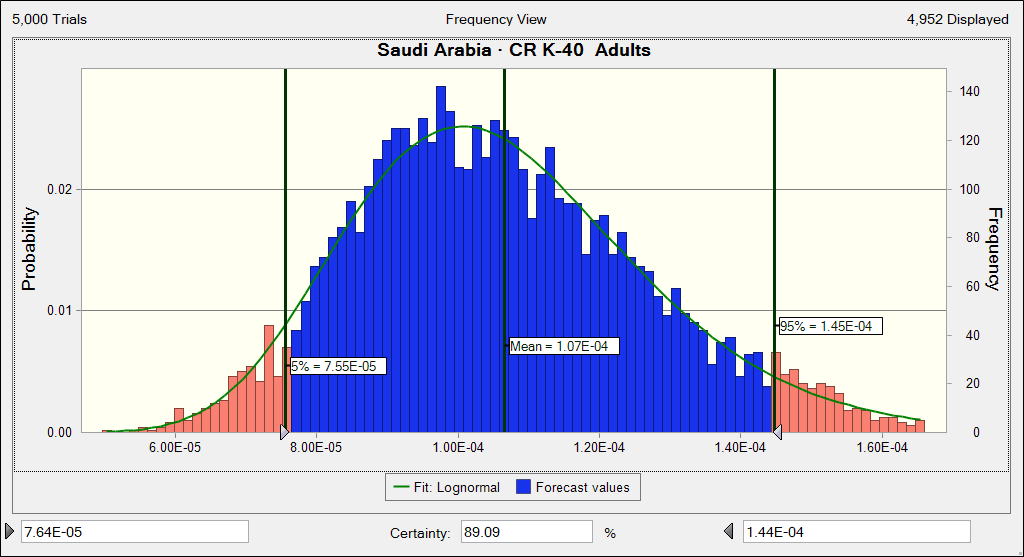


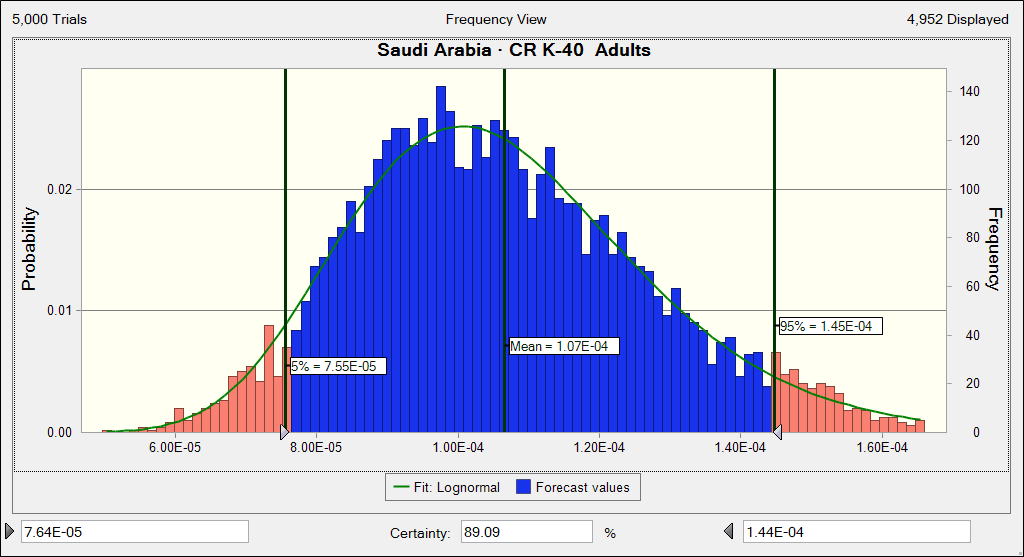


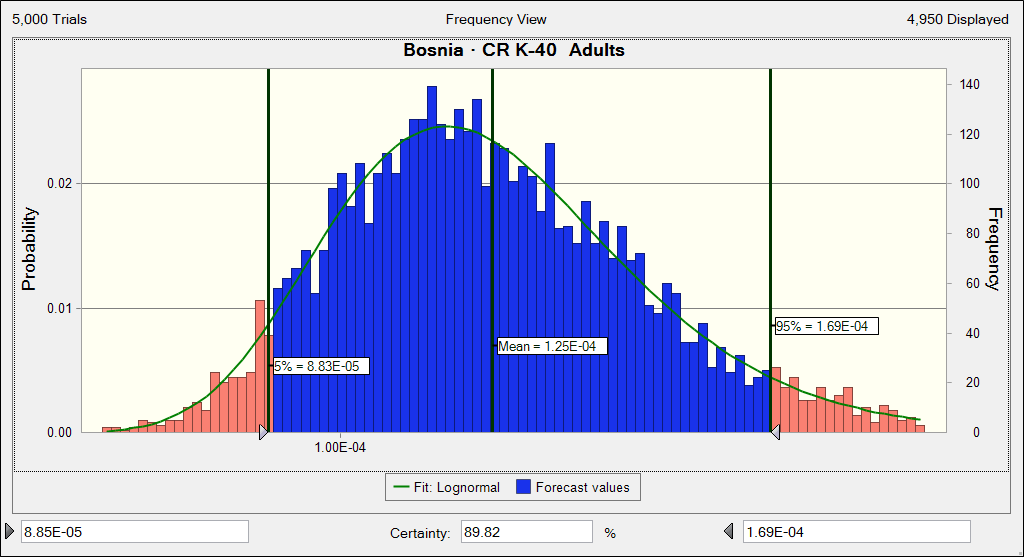


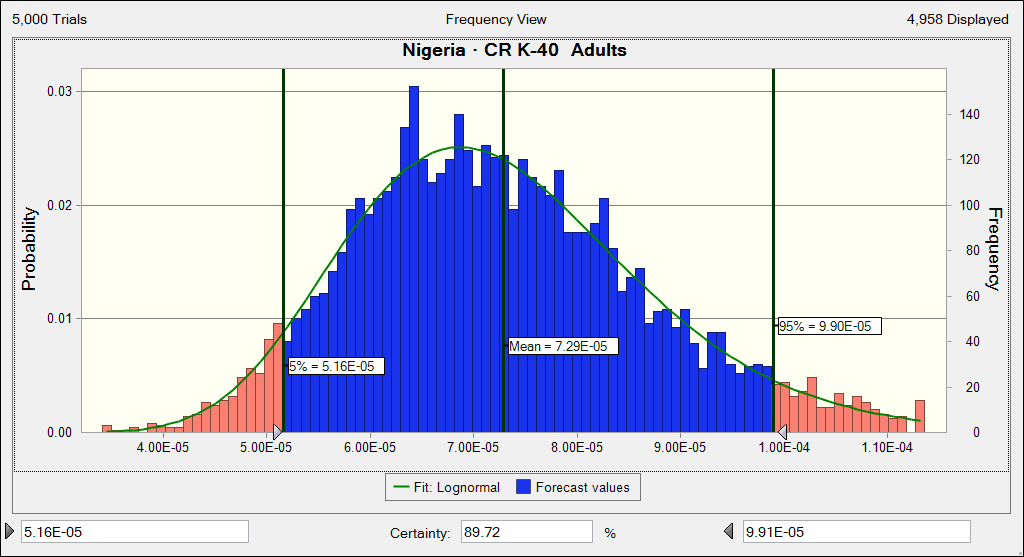


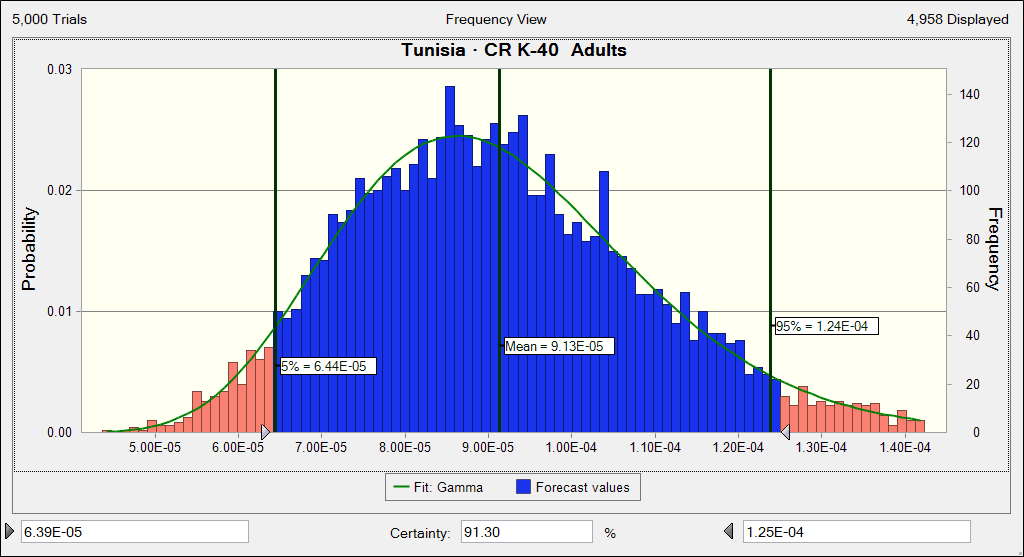


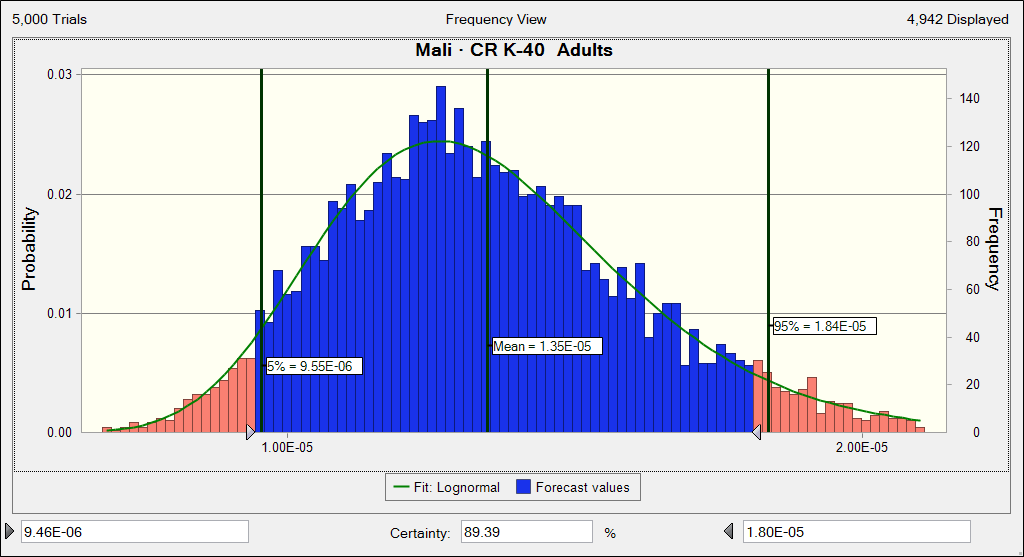


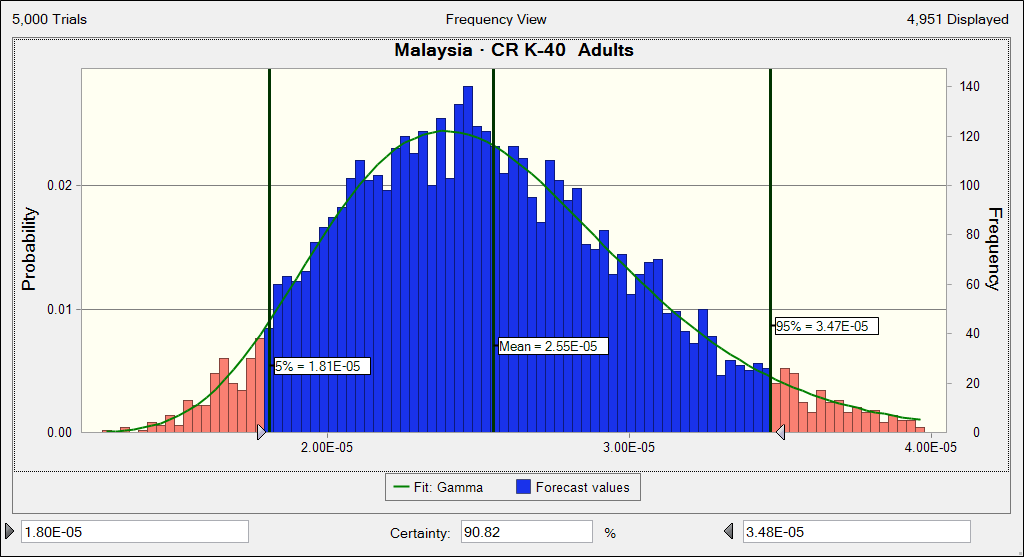


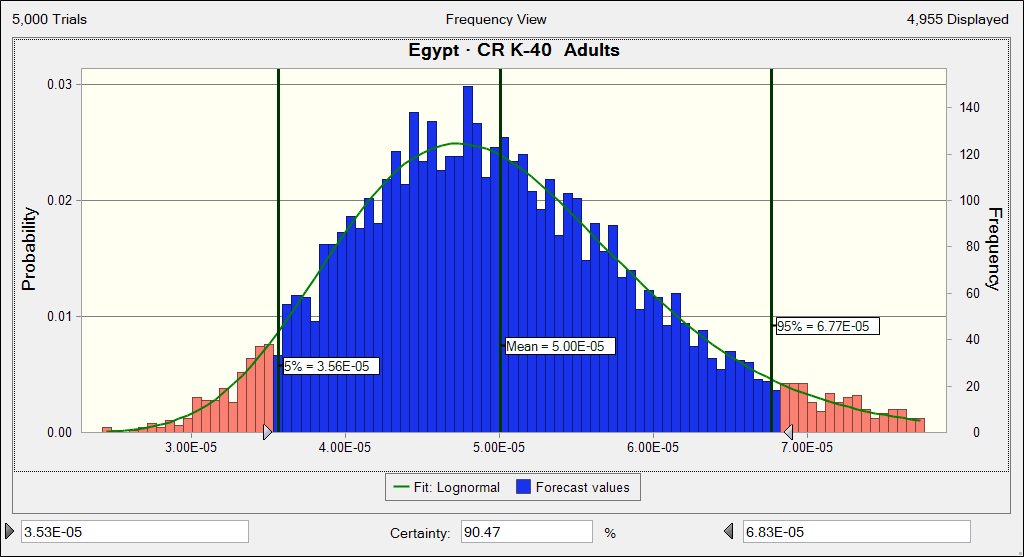


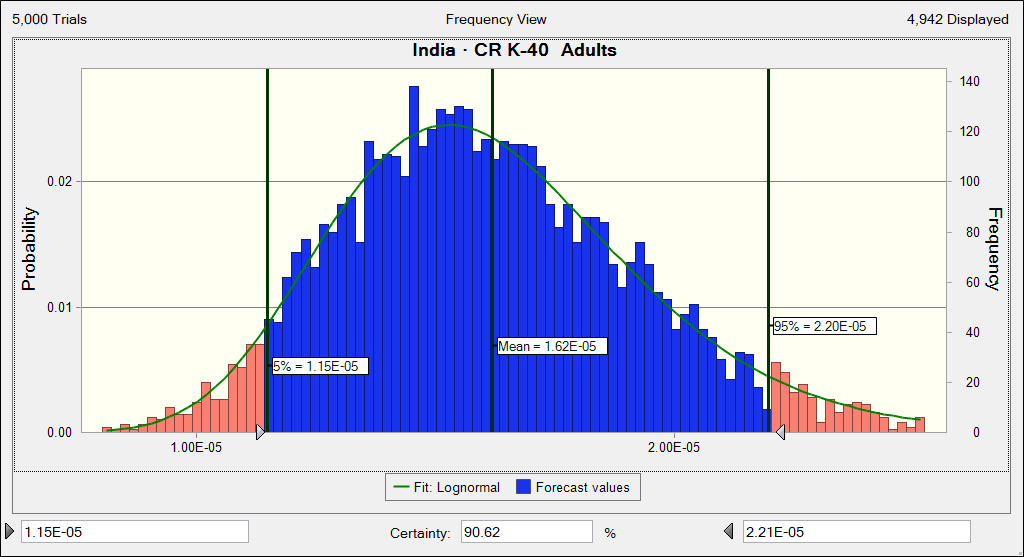


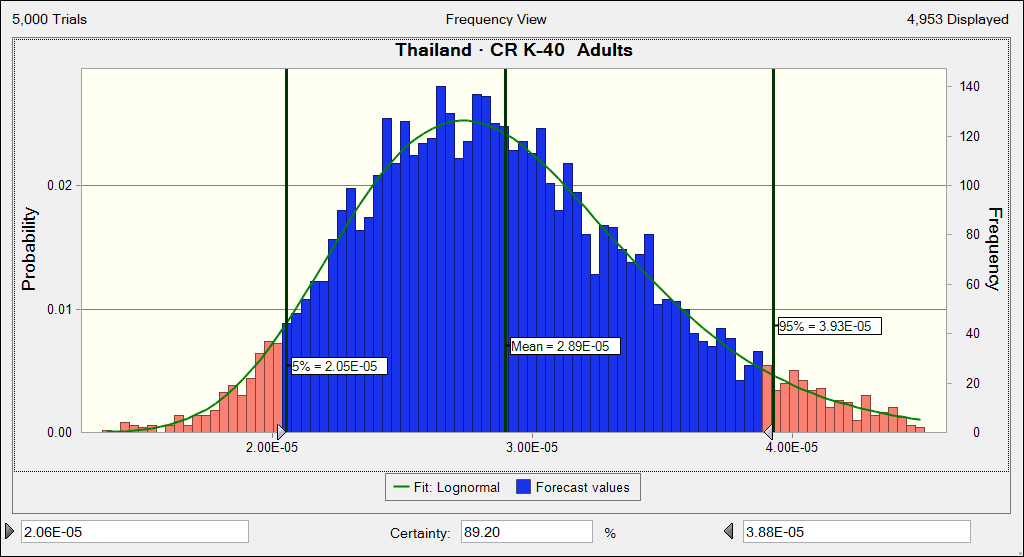


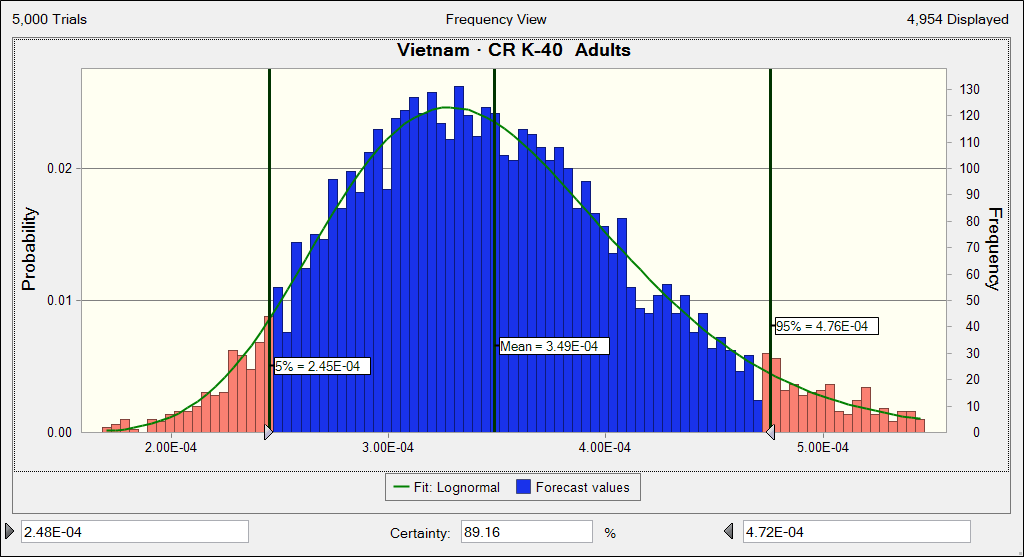


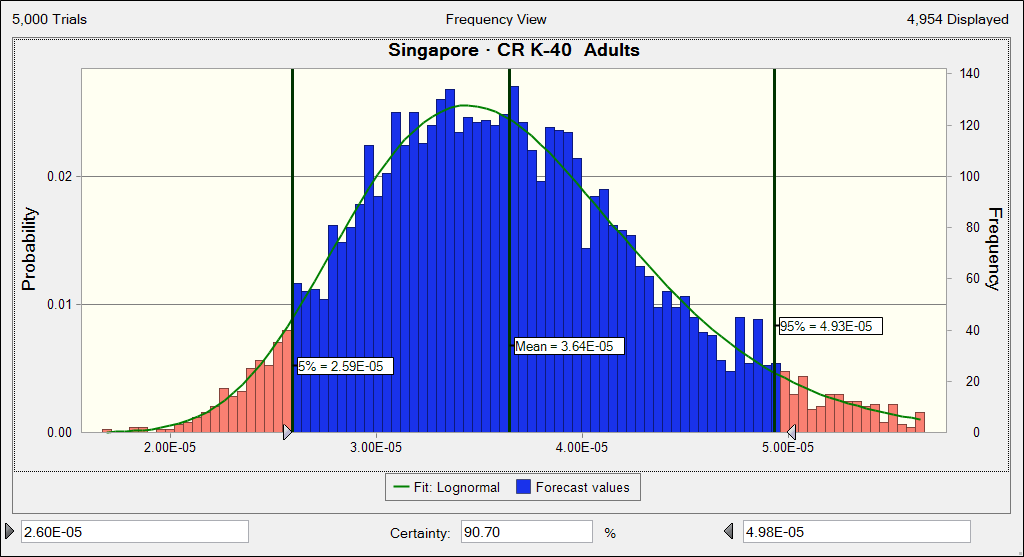


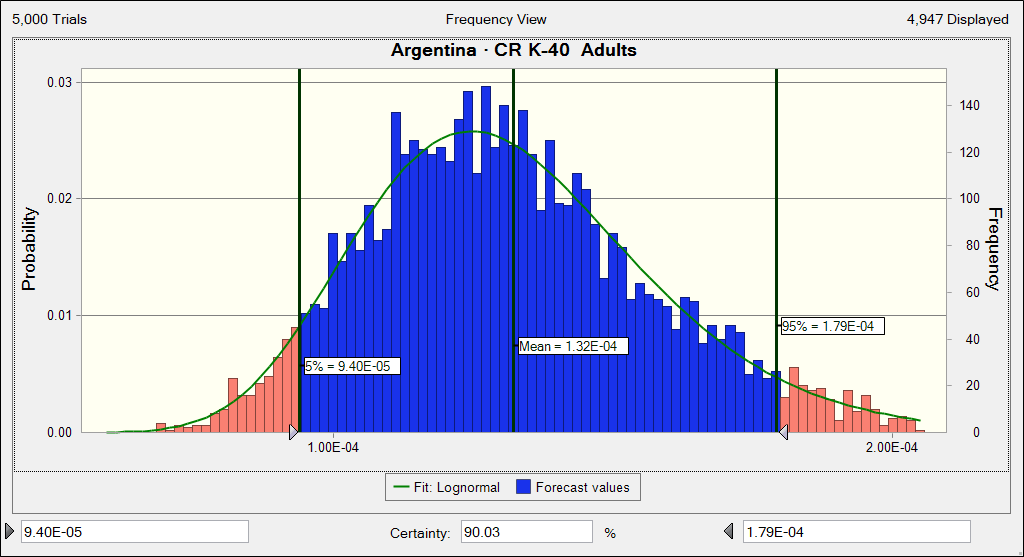


**Supplementary appendix 7.** The MCS model for determine CR of I-131 in milk


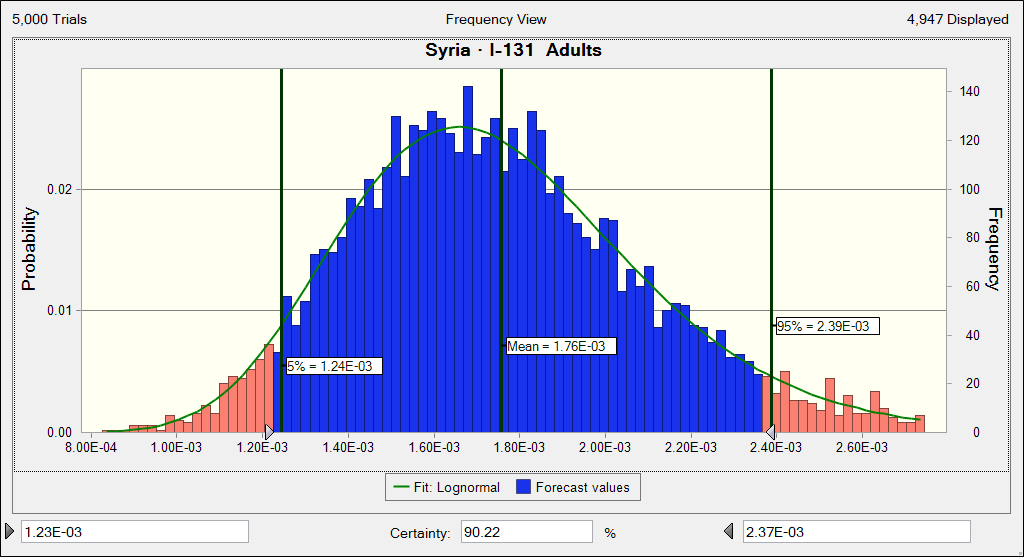


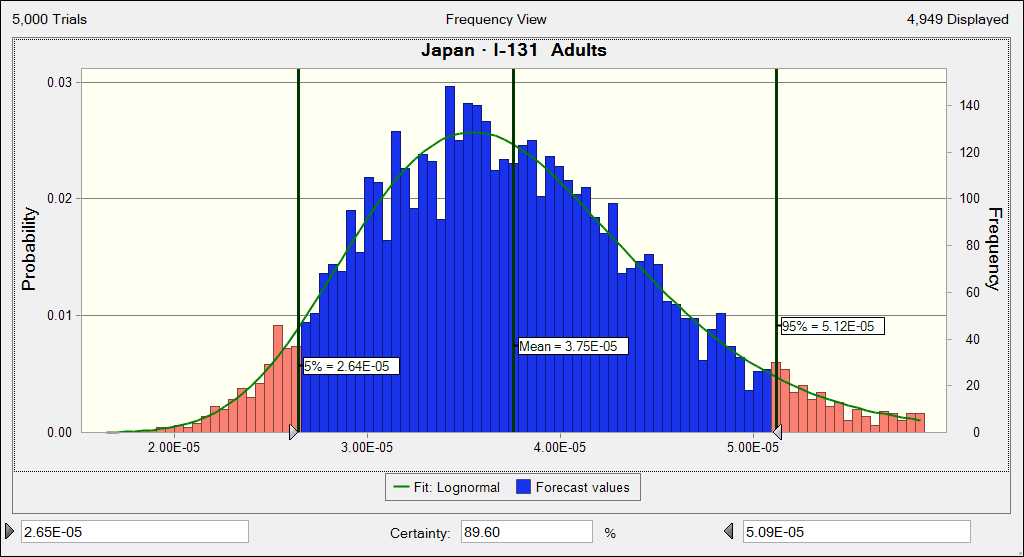


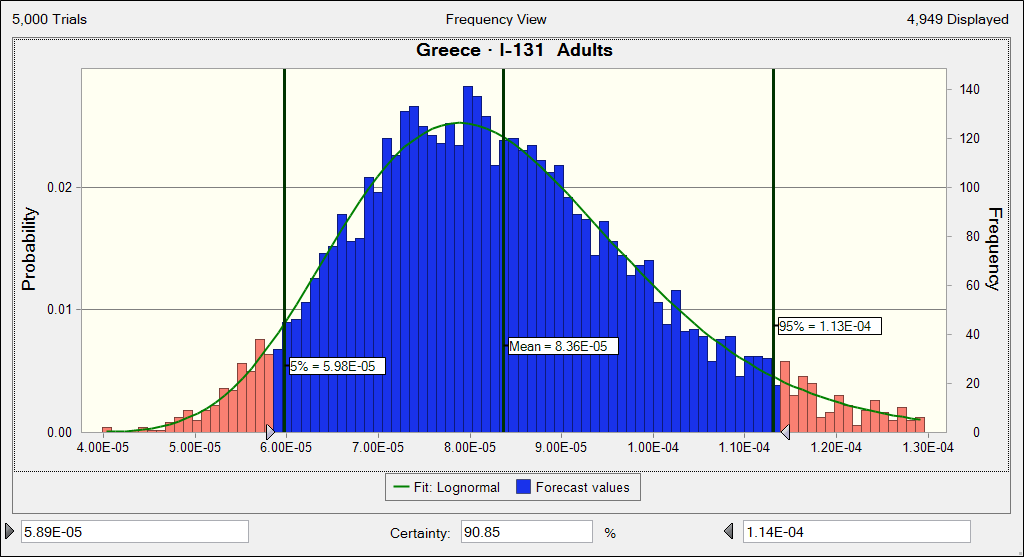


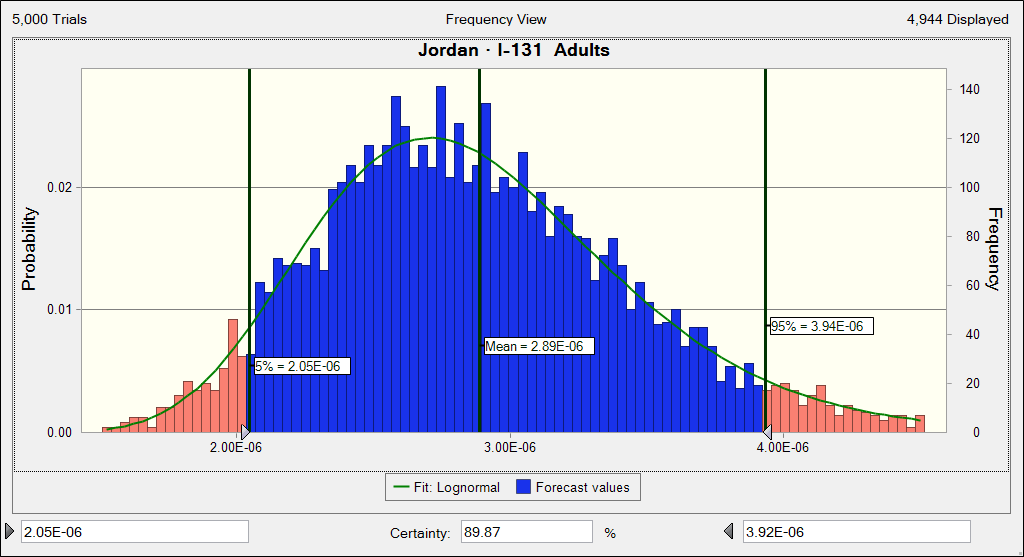


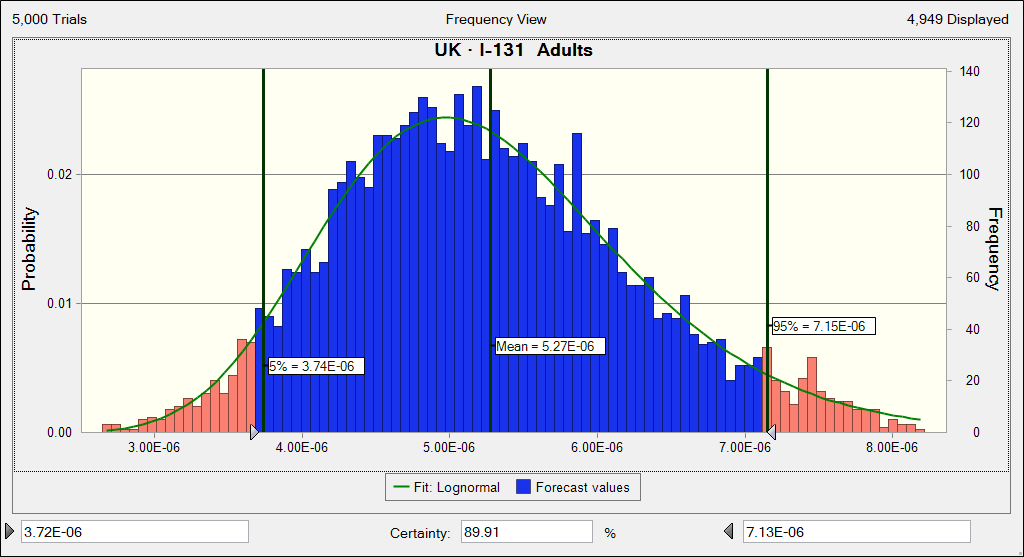


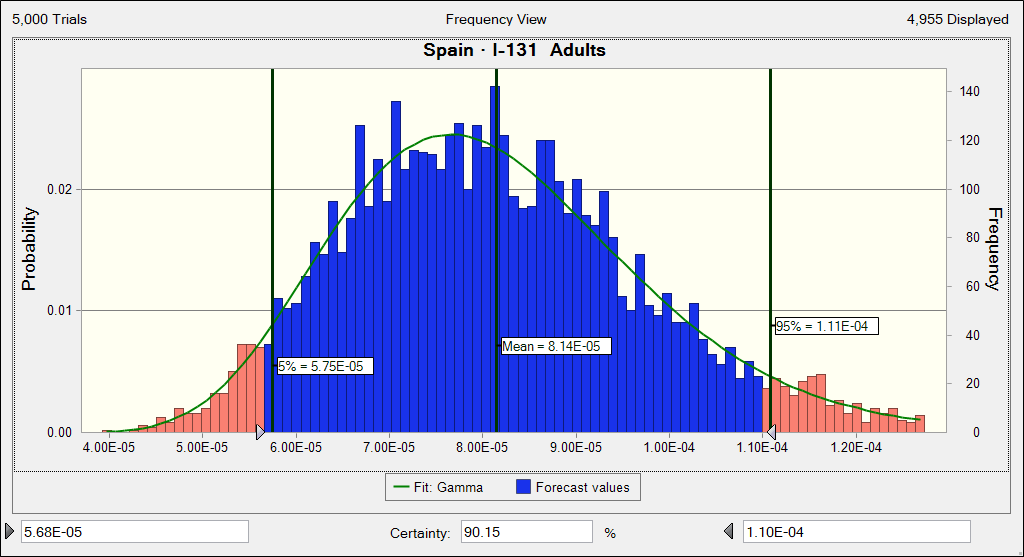


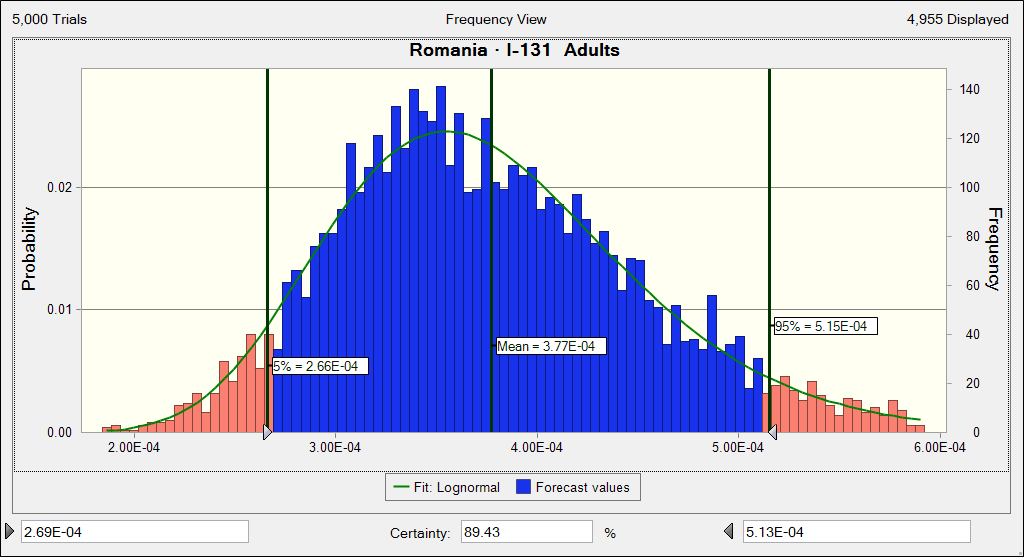


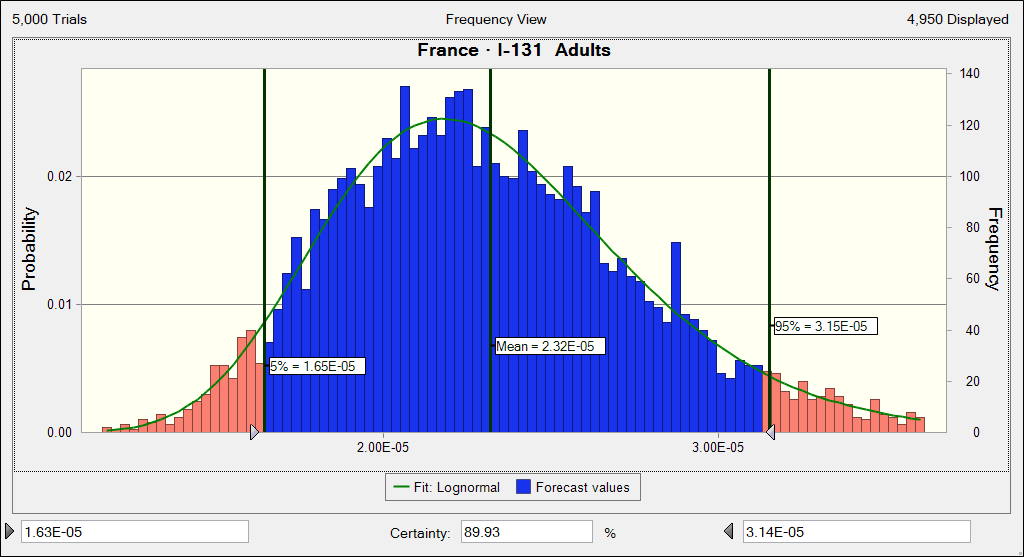


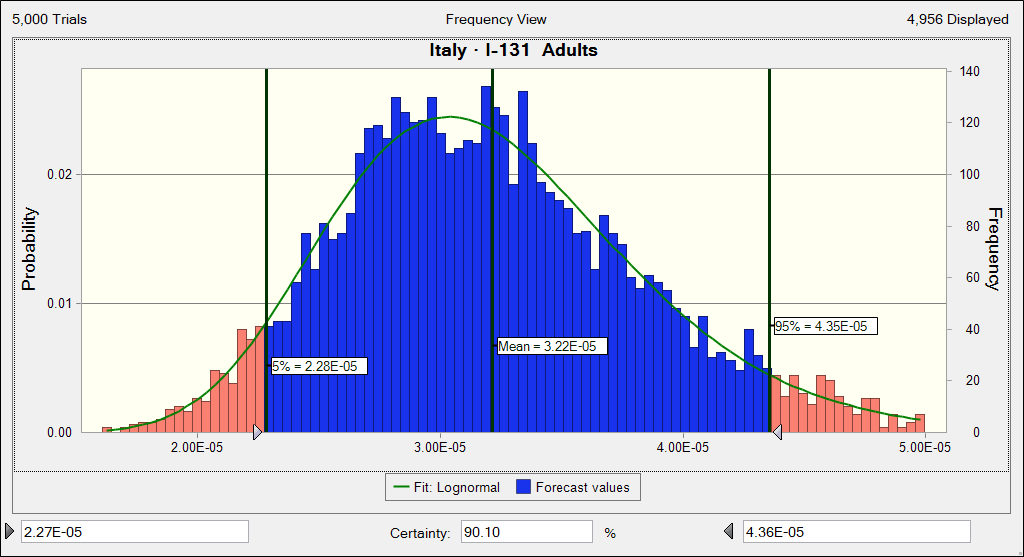


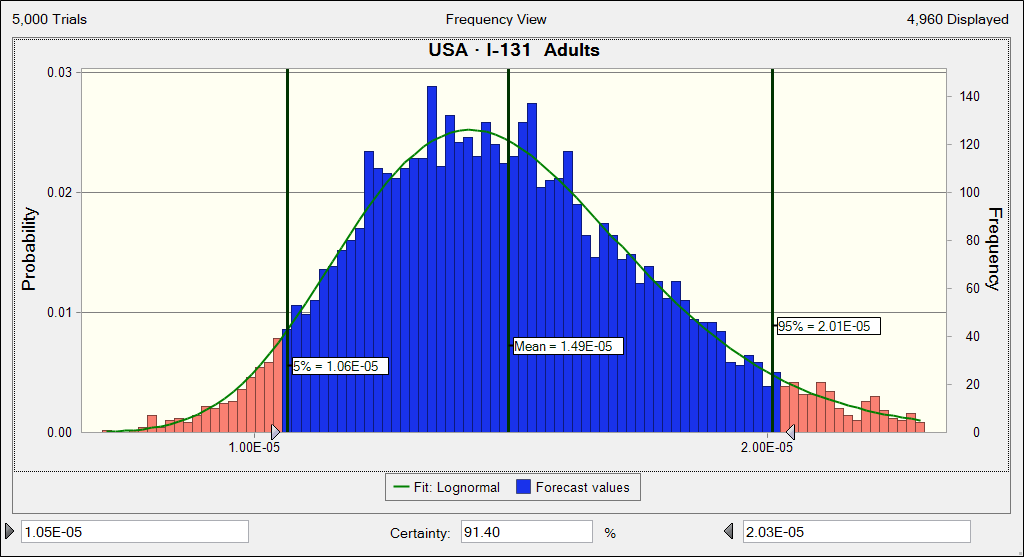


**Supplementary appendix 8.** The MCS model for determine CR of Pb-210 in milk


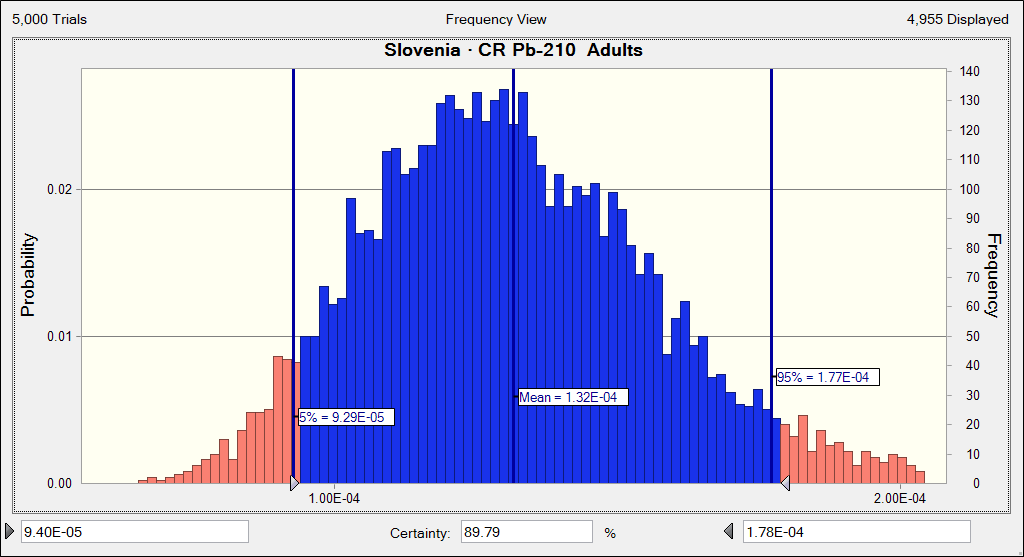


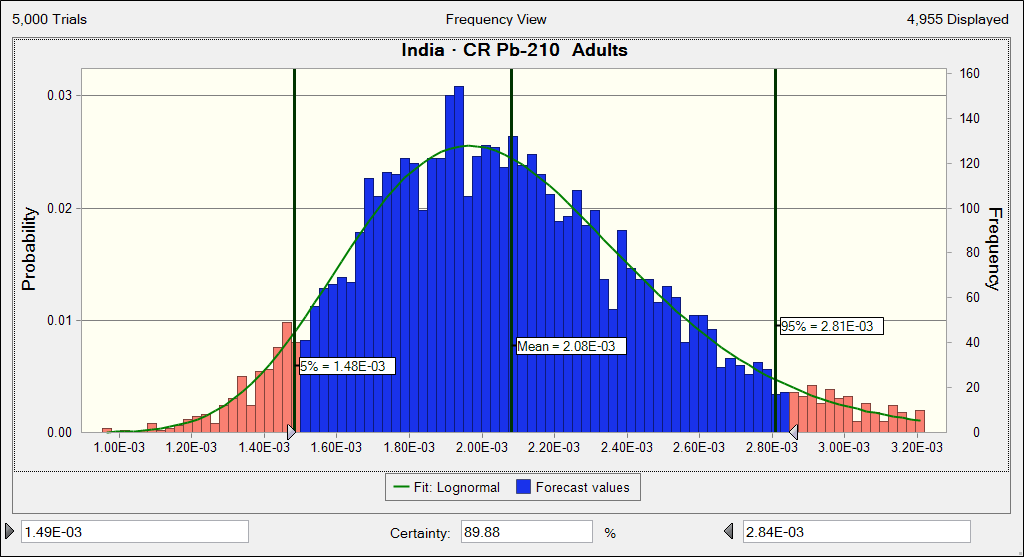


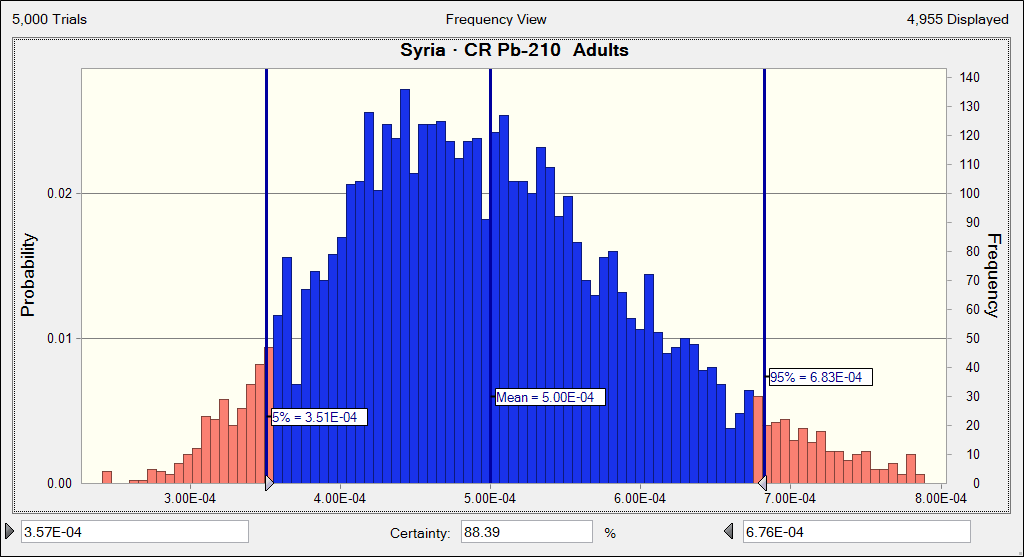


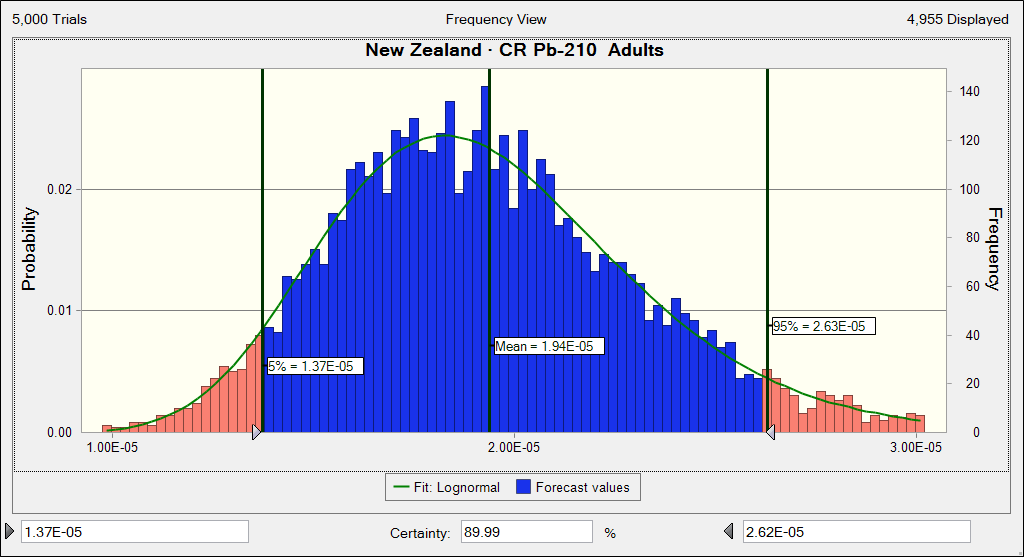


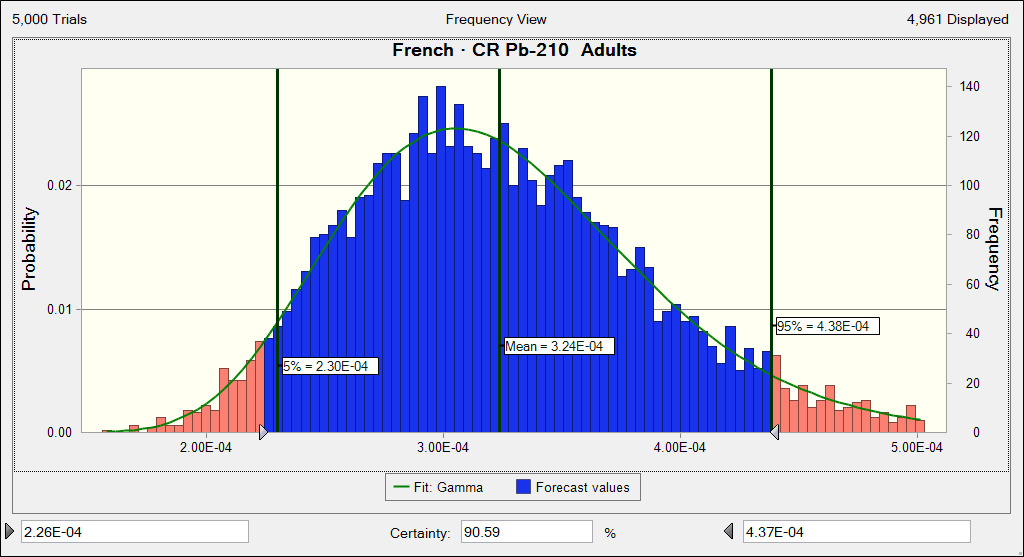


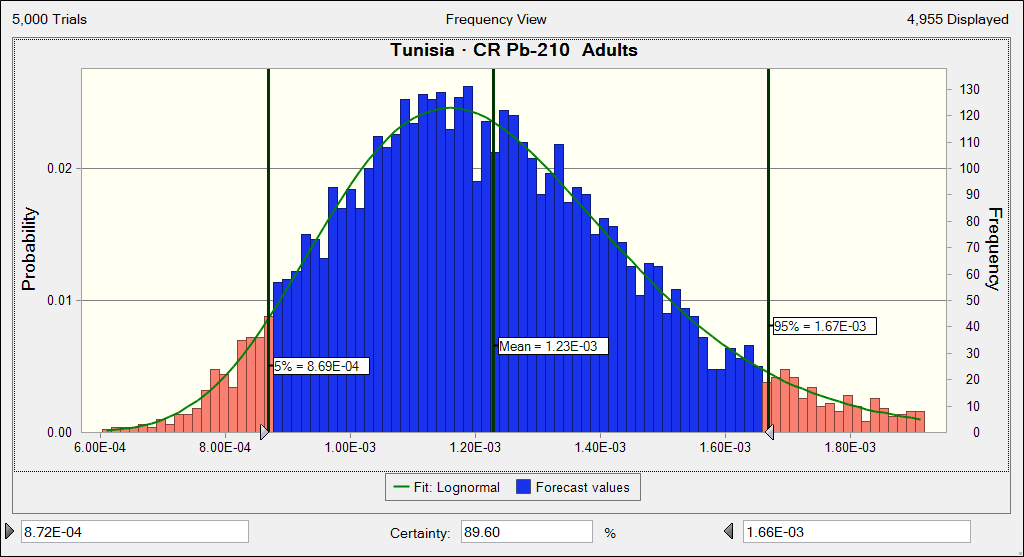


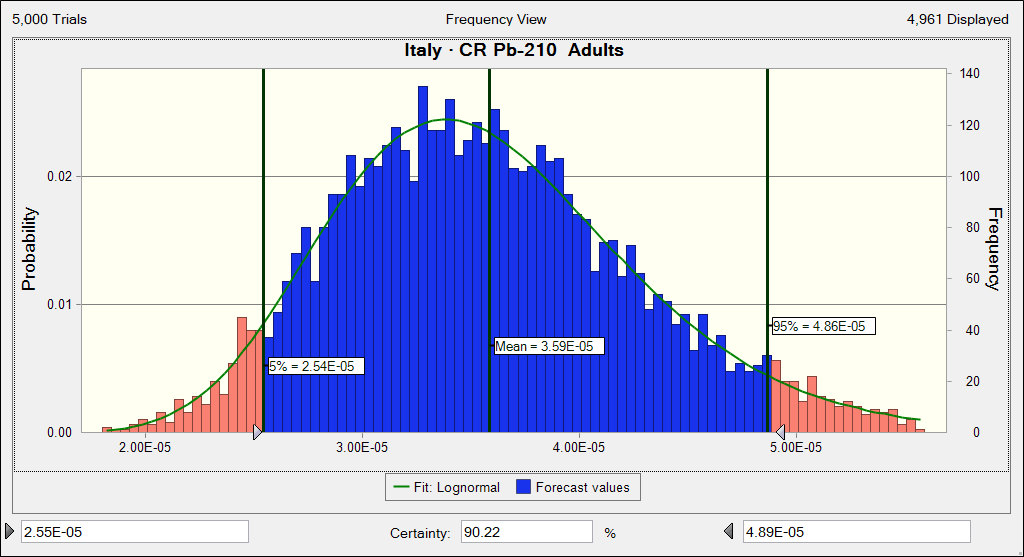


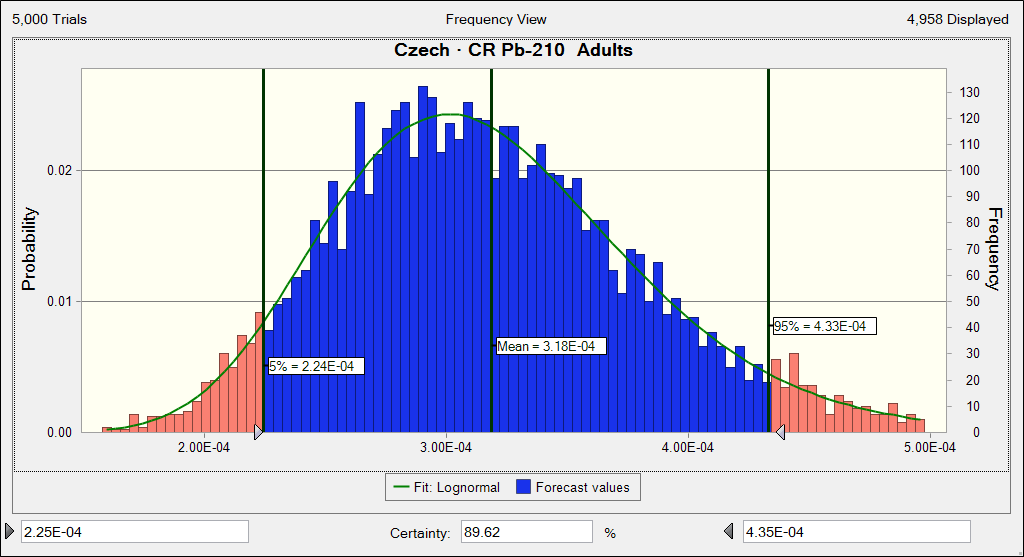


**References**

1. Statista. *Annual consumption of fluid cow milk worldwide in 2024, by country.*[*https://www.statista.com/statistics/272003/global-annual-consumption-of-milk-by-region/?srsltid=AfmBOoqAHiJz8lo2B_BKpvjPcYkCIKoEc0MBW84JL-tcevXf_qkQ5mOz*](https://www.statista.com/statistics/272003/global-annual-consumption-of-milk-by-region/?srsltid=AfmBOoqAHiJz8lo2B_BKpvjPcYkCIKoEc0MBW84JL-tcevXf_qkQ5mOz). 2024.

2. Alimam, W. and A. Auvinen, *Cancer risk due to ingestion of naturally occurring radionuclides through drinking water: A systematic review.* Sci Total Environ, 2025. **968**: p. 178849.

3. USEPA, *United States Environmental Protection Agency. EPA 402-R-99-001Cancer Risk Coefficients for Environmental Exposure to Radionuclides.* 1999.

4. da Silva, R.C., et al., *Radiological evaluation of Ra-226, Ra-228 and K-40 in tea samples: A comparative study of effective dose and cancer risk.* Applied Radiation and Isotopes, 2020. **165**: p. 109326.

1. Lettner, H., et al., *137Cs and 90Sr transfer to milk in Austrian alpine agriculture.* Journal of Environmental Radioactivity, 2007. **98**(1-2): p. 69-84.

2. Gastberger, M., et al., *90Sr and 137Cs in environmental samples from Dolon near the Semipalatinsk nuclear test site.* Health Physics, 2000. **79**(3): p. 257-265.

3. Desimoni, J., et al., *Activity levels of gamma-emitters in Argentinean cow milk.* Journal of Food Composition and Analysis, 2009. **22**(3): p. 250-253.

4. BARTUSKOVÁ, M., et al., *Activity of natural radionuclide 210 Pb in Czech foodstuffs and its annual intake.* Czech Journal of Food Sciences, 2019. **37**(6).

5. Treinen, R.M., *An analysis of the intake of iodine-131 by a dairy herd post-Fukushima and the subsequent excretion in milk.* Journal of Environmental Radioactivity, 2015. **149**: p. 135-143.

6. Ong, J.X., et al., *An assessment of natural and artifical radionuclide content in powdered milk consumed by infants and toddlers in Singapore.* Journal of Radioanalytical and Nuclear Chemistry, 2024. **333**(2): p. 951-959.

7. Meli, M.A., et al., *Assessment of 210Po in Italian diet.* Food chemistry, 2014. **155**: p. 87-90.

8. Duong, V.-H., et al., *Assessment of 232 Th, 226 Ra, 137 Cs, and 40 K concentrations and annual effective dose due to the consumption of Vietnamese fresh milk.* Journal of Radioanalytical and Nuclear Chemistry, 2021. **328**: p. 1399-1404.

9. Poltabtim, W. and K. Saenboonruang, *Assessment of activity concentrations and their associated radiological health risks in commercial infant formulas in Thailand.* Chiang Mai Journal of Science, 2019. **46**(4): p. 778-786.

10. Patra, A., et al., *Assessment of ingestion dose due to radioactivity in selected food matrices and water near Vizag, India.* Journal of Radioanalytical and Nuclear Chemistry, 2014. **300**: p. 903-910.

11. Salahel Din, K., *Assessment of natural and artificial radioactivity in infants’ powdered milk and their associated radiological health risks.* Journal of Radioanalytical and Nuclear Chemistry, 2020. **324**: p. 977-981.

12. Priharti, W., et al., *Assessment of radiation hazard indices arising from natural radionuclides content of powdered milk in Malaysia.* Journal of Radioanalytical and Nuclear Chemistry, 2016. **307**: p. 297-303.

13. Coulibaly, A., D.O. Kpeglo, and E.O. Darko, *Assessment of radiological hazards in some foods products consumed by the Malian population using gamma spectrometry.* Journal of Radiation Protection and Research, 2023. **48**(2): p. 84-89.

14. Machraoui, S., et al., *Assessment of radionuclide transfer factors and transfer coefficients near phosphate industrial areas of South Tunisia.* Environmental Science and Pollution Research, 2019. **26**: p. 28341-28351.

15. Sebezhko, O., et al., *Comparative assessment of radioactive strontium and cesium contents in the feedstuffs and dairy products of Western Siberia.* Indian Journal of ecology, 2017. **44**(3): p. 662-666.

16. Agbalagba, E.O., H.O. Agbalagba, and G.O. Avwiri, *Cost-benefit analysis approach to risk assessment of natural radioactivity in powdered and liquid milk products consumed in Nigeria.* Environmental forensics, 2016. **17**(3): p. 191-202.

17. Kakimov, A., et al., *Cs-137 in milk, vegetation, soil, and water near the former Soviet Union’s Semipalatinsk Nuclear Test Site.* Environmental Science and Pollution Research, 2016. **23**: p. 4931-4937.

18. Labunska, I., et al., *Current radiological situation in areas of Ukraine contaminated by the Chernobyl accident: Part 1. Human dietary exposure to Caesium-137 and possible mitigation measures.* Environment International, 2018. **117**: p. 250-259.

19. Duyssembaev, S., et al., *Determination of Cs-137 concentration in some environmental samples around the Semipalatinsk nuclear test site in the Republic of Kazakhstan.* Annual Research & Review in Biology, 2017. **15**(4): p. 1-8.

20. Muftić, E., et al., *Distribution of radiocaesium (137Cs) and radiopotassium (40K) during the cheese production.* International Journal of Dairy Technology, 2023. **76**(4): p. 1030-1036.

21. Bartusková, M., et al., *Doses from Cs-137 and Sr-90 to Czech population due to milk consumption.* Radioprotection, 2017. **52**(3): p. 171-176.

22. Barsanti, M., et al., *Environmental radioactivity analyses in Italy following the Fukushima Dai-ichi nuclear accident.* Journal of environmental radioactivity, 2012. **114**: p. 126-130.

23. Chae, J.-S., et al., *Estimation of annual effective dose from ingestion of 40 K and 137 Cs in foods frequently consumed in Korea.* Journal of Radioanalytical and Nuclear Chemistry, 2016. **310**: p. 1069-1075.

24. Perrot, F., et al., *Evidence of 131I and 134,137 Cs activities in Bordeaux, France due to the Fukushima nuclear accident.* Journal of environmental radioactivity, 2012. **114**: p. 61-65.

25. Ioannidou, A., et al., *Fukushima fallout at Milano, Italy.* Journal of environmental radioactivity, 2012. **114**: p. 119-125.

26. Shozugawa, K., et al., *High-sensitivity determination of radioactive cesium in Japanese foodstuffs: 3 years after the Fukushima accident.* Journal of Radioanalytical and Nuclear Chemistry, 2016. **307**: p. 2117-2122.

27. Cosma, C., et al., *Indicators of the Fukushima radioactive release in NW Romania.* Journal of environmental radioactivity, 2012. **114**: p. 94-99.

28. Baeza, A., et al., *Influence of the Fukushima Dai-ichi nuclear accident on Spanish environmental radioactivity levels.* Journal of environmental radioactivity, 2012. **114**: p. 138-145.

29. Corcho-Alvarado, J., et al., *Long-term behavior of 90Sr and 137Cs in the environment: case studies in Switzerland.* Journal of environmental radioactivity, 2016. **160**: p. 54-63.

30. Pálsson, S.E., et al., *Long-term transfer of global fallout 137 Cs to cow’s milk in Iceland.* Environmental monitoring and assessment, 2012. **184**: p. 7221-7234.

31. Yii, M.-W., *Measurement of activity concentrations in powdered milk and estimation of the corresponding annual effective dose.* Journal of Radioanalytical and Nuclear Chemistry, 2019. **320**(1): p. 193-199.

32. Renaud, P., et al., *226Ra and 228Ra activities in French foodstuffs.* Radioprotection, 2015. **50**(2): p. 111-115.

33. Uwatse, O.B., et al., *Measurement of natural and artificial radioactivity in infant powdered milk and estimation of the corresponding annual effective dose.* Environmental Engineering Science, 2015. **32**(10): p. 838-846.

34. Jemii, E. and T. Alharbi, *Measurements of natural radioactivity in infant formula and radiological risk assessment.* Journal of radioanalytical and Nuclear Chemistry, 2018. **315**: p. 157-161.

35. Alharshan, G., et al., *Measuring the radioactivity concentration of 40 K and 137 Cs and calculating the annual internal doses from ingesting liquid and powdered milk.* radiochemistry, 2017. **59**: p. 98-103.

36. Mitrović, B., et al., *Natural and anthropogenic radioactivity in the environment of mountain region of Serbia.* Journal of environmental monitoring, 2009. **11**(2): p. 383-388.

37. Mitrović, B., et al., *Natural and anthropogenic radioactivity in the environment of Kopaonik mountain, Serbia.* Environmental Pollution, 2016. **215**: p. 273-279.

38. Pearson, A.J., et al., *Natural and anthropogenic radionuclide activity concentrations in the New Zealand diet.* Journal of environmental radioactivity, 2016. **151**: p. 601-608.

39. Desideri, D., et al., *Natural radionuclides in Italian diet and their annual intake.* Journal of Radioanalytical and Nuclear Chemistry, 2014. **299**: p. 1461-1467.

40. Al-Masri, M., et al., *Natural radionuclides in Syrian diet and their daily intake.* Journal of Radioanalytical and Nuclear Chemistry, 2004. **260**: p. 405-412.

41. MIHAELA, T., K. OTTO, and T. OVIDIU, *Naturally occurring 137Cs, 90Sr and 226Ra radionuclides in raw milk in the Sibiu province of Romania.* International journal of dairy technology, 2012. **65**(4): p. 511-515.

42. Beresford, N.A., et al., *Observations of Fukushima fallout in Great Britain.* Journal of environmental radioactivity, 2012. **114**: p. 48-53.

43. AbuSaleem, K., et al., *Pre-commissioning baseline activity levels in plant leaves and cow-milk samples around the Jordan Research and Training Reactor.* Journal of Radioanalytical and Nuclear Chemistry, 2021. **330**(1): p. 77-82.

44. El Samad, O., et al., *Public exposure to radioactivity levels in the Lebanese environment.* Environmental Science and Pollution Research, 2017. **24**: p. 2010-2018.

45. Manolopoulou, M., et al., *Radiation measurements and radioecological aspects of fallout from the Fukushima nuclear accident.* Journal of Radioanalytical and Nuclear Chemistry, 2012. **292**(1): p. 155-159.

46. Panov, A.V., et al., *Radiation monitoring of foodstuffs and drinking water in the vicinity of the Rooppur Nuclear Power Plant (People’s Republic of Bangladesh).* Journal of Food Composition and Analysis, 2022. **114**: p. 104732.

47. Thakur, P., S. Ballard, and R. Nelson, *Radioactive fallout in the United States due to the Fukushima nuclear plant accident.* Journal of Environmental Monitoring, 2012. **14**(5): p. 1317-1324.

48. Kritidis, P., et al., *Radioactive pollution in Athens, Greece due to the Fukushima nuclear accident.* Journal of environmental radioactivity, 2012. **114**: p. 100-104.

49. Manolopoulou, M., et al., *Radioecological indexes of fallout measurements from the Fukushima nuclear accident.* Ecological indicators, 2013. **25**: p. 197-199.

50. Samad, A.I., A.H. Ahmed, and S.T. Ahmad, *Radiological health assessment of infant milk in Erbil Governorate, Iraq.* Environmental Monitoring and Assessment, 2023. **195**(3): p. 419.

51. Saleh, I.H., et al., *Radiological Study on Soils, Foodstuff and Fertilizers in the Alexandria Region, Egypt.* Turkish Journal of Engineering & Environmental Sciences, 2007. **31**(1).

52. Benaissa, K., et al., *Radionuclide assessment in imported powdered infant milk consumed in algeria and radiation hazard indices.* Radiochemistry, 2020. **62**: p. 673-680.

53. Duyssembaev, S., et al., *Radionuclide content in the soil-water-plant-livestock product system in east Kazakhstan.* Polish Journal of Environmental Studies, 2014. **23**(6): p. 1983-1993.

54. Al-Masri, M., et al., *Radionuclide transfer from feed to camel milk.* Journal of environmental radioactivity, 2014. **132**: p. 8-14.

55. Ioannidou, A., et al., *Radionuclides from Fukushima accident in Thessaloniki, Greece (40 N) and Milano, Italy (45).* Journal of Radioanalytical and Nuclear Chemistry, 2014. **299**: p. 855-860.

56. Giri, S., et al., *Risk assessment due to ingestion of natural radionuclides and heavy metals in the milk samples: a case study from a proposed uranium mining area, Jharkhand.* Environmental monitoring and assessment, 2011. **175**: p. 157-166.

57. Kılıç, Ö., et al., *Seasonal variations of trace elements and radio-activity concentrations in raw cow's milk samples collected from West Thrace region, Turkey.* 2010.

58. Albers, B., et al., *Soil-to-plant and plant-to-cow’s milk transfer of radiocaesium in alpine pastures: significance of seasonal variability.* Chemosphere, 2000. **41**(5): p. 717-723.

59. Rejah, B.K., *Specific activities of natural radionuclides and annual effective dose due to the intake of some types of children powdered milk available in Baghdad Markets.* Baghdad Science Journal, 2017. **14**(3): p. 0619-0619.

60. Kakimov, A., et al., *Specific activity of Cs-137 in milk of Semey region of East Kazakhstan area.* Annual Research & Review in Biology, 2017. **12**(5): p. ARRB. 33391-ARRB. 33391.

61. Terada, H., et al., *Total diet study to assess radioactive Cs and 40K levels in the Japanese population before and after the Fukushima Daiichi Nuclear Power Plant accident.* International journal of environmental research and public health, 2020. **17**(21): p. 8131.

62. Mărgineanu, R., et al., *Traces of radioactive 131I in rainwater and milk samples in Romania.* Environmental Research Letters, 2011. **6**(3): p. 034011.

63. Jia, G. and L. Magro, *Transfer behaviors of 90Sr and 137Cs from soil to grass to cow milk under natural conditions in Central Italy and their exposure risk.* Journal of Radioanalytical and Nuclear Chemistry, 2021. **330**(3): p. 845-856.

64. Koivurova, M., A.-P. Leppänen, and A. Kallio, *Transfer factors and effective half-lives of 134Cs and 137Cs in different environmental sample types obtained from Northern Finland: case Fukushima accident.* Journal of environmental radioactivity, 2015. **146**: p. 73-79.

65. Guillén, J., et al., *Transfer of radionuclides and stable elements to foodstuffs in Mediterranean ecosystems.* Journal of Environmental Radioactivity, 2020. **223**: p. 106379.

66. Trdin, M. and L. Benedik, *Uranium, polonium and thorium in infant formulas (powder milk) and assessment of a cumulative ingestion dose.* Journal of Food Composition and Analysis, 2017. **64**: p. 198-202.

67. Statista. *Annual consumption of fluid cow milk worldwide in 2024, by country.*[*https://www.statista.com/statistics/272003/global-annual-consumption-of-milk-by-region/?srsltid=AfmBOoqAHiJz8lo2B_BKpvjPcYkCIKoEc0MBW84JL-tcevXf_qkQ5mOz*](https://www.statista.com/statistics/272003/global-annual-consumption-of-milk-by-region/?srsltid=AfmBOoqAHiJz8lo2B_BKpvjPcYkCIKoEc0MBW84JL-tcevXf_qkQ5mOz). 2024.

68. Alimam, W. and A. Auvinen, *Cancer risk due to ingestion of naturally occurring radionuclides through drinking water: A systematic review.* Sci Total Environ, 2025. **968**: p. 178849.

69. USEPA, *United States Environmental Protection Agency. EPA 402-R-99-001Cancer Risk Coefficients for Environmental Exposure to Radionuclides.* 1999.

70. da Silva, R.C., et al., *Radiological evaluation of Ra-226, Ra-228 and K-40 in tea samples: A comparative study of effective dose and cancer risk.* Applied Radiation and Isotopes, 2020. **165**: p. 109326.

1. Standard deviation [↑](#footnote-ref-1)
